# Evolutionary origins of the prolonged extant squamate radiation

**DOI:** 10.1038/s41467-022-34217-5

**Published:** 2022-11-29

**Authors:** Chase D. Brownstein, Dalton L. Meyer, Matteo Fabbri, Bhart-Anjan S. Bhullar, Jacques A. Gauthier

**Affiliations:** 1 Department of Ecology and Evolutionary Biology, Yale University, New Haven, CT USA; 2 Stamford Museum and Nature Center, Stamford, CT USA; 3 Department of Earth and Planetary Sciences, Yale University, New Haven, CT USA; 4 Negaunee Integrative Research Center, Field Museum of Natural History, Chicago, IL USA; 5 Yale Peabody Museum, Yale University, New Haven, CT USA

**Keywords:** Palaeontology, Phylogenetics, Taxonomy

## Abstract

Squamata is the most diverse clade of terrestrial vertebrates. Although the origin of pan-squamates lies in the Triassic, the oldest undisputed members of extant clades known from nearly complete, uncrushed material come from the Cretaceous. Here, we describe three-dimensionally preserved partial skulls of two new crown lizards from the Late Jurassic of North America. Both species are placed at the base of the skink, girdled, and night lizard clade Pan-Scincoidea, which consistently occupies a position deep inside the squamate crown in both morphological and molecular phylogenies. The new lizards show that several features uniting pan-scincoids with another major lizard clade, the pan-lacertoids, in trees using morphology were convergently acquired as predicted by molecular analyses. Further, the palate of one new lizard bears a handful of ancestral saurian characteristics lost in nearly all extant squamates, revealing an underappreciated degree of complex morphological evolution in the early squamate crown. We find strong evidence for close relationships between the two new species and Cretaceous taxa from Eurasia. Together, these results suggest that early crown squamates had a wide geographic distribution and experienced complicated morphological evolution even while the Rhynchocephalia, now solely represented by the tuatara, was the dominant clade of lepidosaurs.

## Abstract (web summary)

Here, the authors present two well preserved fossil lizard skulls from the Late Jurassic of North America. These fossils, placed at the base of the clade Pan-Scincoidea, suggest that squamates had a wide geographic distribution and preserve characteristics that show the complex early evolutionary history of squamate anatomy.

## Introduction

The evolution of pan-squamates spans more than 250 million years of Earth history^1–8^ Although there are more than 11,000 extant lizard species^9^, the fossil record for the first half of squamate evolution remains meager. Putative snakes^10^, gekkotans^11^, and scincoids^12^ have been reported from the Jurassic, but these have inconsistent placements within and outside the crown in different analyses^4–6,10,13^. Even the most complete crushed skeletons are variously recovered within or on the stem of different major squamate crown clades^4–6,14,15^ or outside the crown altogether^16^. These factors have obscured the origins of the anatomical disparity found in extant squamates^15,17–23^ and the timing of their initial radiation, which took place when rhynchocephalians were far more abundant, speciose, geographically widespread, and ecologically disparate^24–27^.

Here, we use high-resolution computed tomography (CT) scans to describe two lizards represented by articulated, three-dimensional skulls from the Late Jurassic (~145 Ma) Morrison Formation of western North America. One of these was preliminarily described by Evans and Chure^28^ and is the most complete Jurassic squamate from the Americas. Our results show that both new species are deeply nested within the squamate crown near Cretaceous species from Eurasia. As the oldest crown squamates known from three-dimensional cranial material, they support the Late Triassic to Middle Jurassic molecular divergence times for most major extant squamate clades^8^. The skulls of the Morrison lizards also suggest that several apomorphies of Scincomorpha, a classic clade consisting of scincoids and lacertoids that is consistently supported in morphological phylogenies^15,29^, were independently acquired in Pan-Scincoidea (skinks, girdled, and night lizards) and Lacertoidea (amphisbaenians, spectacled, whiptail, and wall lizards). Further, one of the new species possesses ancestral saurian features of the vomer that are lost in nearly all extant squamates, implying an unexpected degree of complex morphological evolution within the squamate crown. The Morrison lizards establish that the squamate backbone clades largely developed their characteristic cranial anatomies during the period when Rhynchocephalia—the extant sister clade to crown Squamata—predominated. Our results help to pinpoint the timing of the turnover events that eventually gave rise to the hyper-diverse extant squamate fauna and reduced rhynchocephalian diversity to a single surviving species, *Sphenodon punctatus*^25,30^.

## Results

### Systematic paleontology of †*Eoscincus ornatus*

Squamata N. M. Oppel 1811 (de Queiroz, K. and Gauthier, J. A. 2020)

Pan-Scincoidea new taxon (see Supplementary Note 7)

†*Eoscincus ornatus* gen. et sp. nov.

#### Holotype

DINO 14864, skull and mandibles lacking the braincase, postorbital skull roof, and quadrate.

#### Locality and horizon

Dinosaur National Monument (DNM) site 412, Utah; Brushy Basin Member (Tithonian, Late Jurassic), Morrison Formation (150–145 Ma^28^).

#### Etymology

Greek *eo* (ηως, dawn) + Latin *scincus* (skink), referencing the age and phylogenetic position of the taxon. Latin *ornatus*, “ornamented” in reference to the vermiculate rugosities on the facial bones.

#### Diagnosis

†*Eoscincus ornatus* is distinguished from other pan-scincoids by the following apomorphies: apex of the ascending ramus of maxilla extends dorsally to more than half the anteroposterior length of the maxilla (shared with specimens referred to †*Paramacellodus*); two rows of teeth on posterior vomer; vomerine teeth larger than palatine and pterygoid teeth; three vomerine foramina; absence of a concavity for the external vomeronasal fenestra on the anterior vomer; splenial extends anteriorly for more than 75% of the length of the tooth row (present in teiids, some lacertids, and †*Parmeosaurus scutatus*, and may be a plesiomorphy). Unlike †*Microteras borealis*, †*E. ornatus* possesses slightly heterodont, unicuspid maxillary dentition.

### Description

**Fig. 1:**
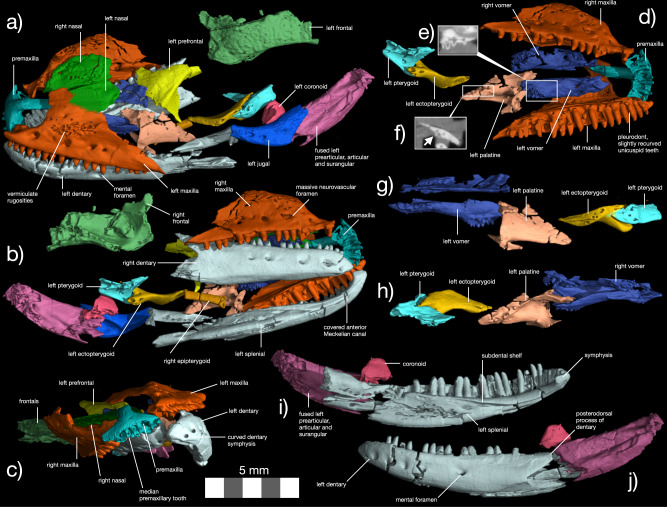
Anatomy of †*Eoscincus ornatus* new taxon. Skull in (**a**) left dorsolateral, (**b**) right ventrolateral, and (**c**) anterior views. Segmented cranium in ventral view (**d**) showing articulated palate, with slices of the vomer in (**e**) lateral view and palatine in (**f**) anterior view revealing the structure of the palatal teeth (indicated by white arrow in (**f**)). Palate in (**g**) left lateral and (**h**) right lateral views. Left mandible in (**i**) medial and (**j**) lateral views. Premaxillae—aquamarine, maxilla—red, nasal—blue, frontal—light green, prefrontal—yellow, vomer—cerulean blue, palatine—pinkish brown, pterygoid—light blue, ectopterygoid—orange, epipterygoid—dark orange, compound bone—dark purple, coronoid—dark pink, dentary and angular—light gray, jugal—blue, unidentified bone (?palpebral)—gray.

The holotype of †*Eoscincus ornatus* includes a complete rostrum and palate, a partial skull roof, and both mandibles (Fig. 1, Figs. S1–5). Pan-scincoid apomorphies of †*E. ornatus* include a coronoid process of the dentary that overlaps the external dentary ramus of the coronoid, a maxilla that contributes to the orbital margin, vermiculate dermal rugosities on the lateral surfaces of the maxillae and frontals, and the protuberance of a posteroventral process on the vomer (Fig. 1a, b, Figs. S1–4)^15,29,31–33^. Other typical pan-scincoid features found in †*E. ornatus* include paired frontals, a prominent subdental shelf, and tooth crown anatomy^15,33–36^. An extended description is in the Supplementary Text and includes comparisons with key Mesozoic and Cenozoic pan-scincoid and paramacellodid taxa.

The premaxilla has a long internasal process that wedges between the nasals and overlaps them narrowly on the midline so that the nasals are nearly in contact ventrally (Fig. 1a–c). The ascending ramus of the maxilla of †*Eoscincus ornatus* is tall and anteroposteriorly extensive^5,15^, exceeding those of most other crown and stem-squamates in size (Fig. 1a–c)^15^. The articulations of the maxilla with the prefrontal, lacrimal, and jugal show the pan-scincoid condition in which the jugal and lacrimal are obscured, allowing the maxilla to form a portion of the anterior margin of the orbit in lateral view^15^. A small quadratojugal process extends posteriorly from the jugal. This process is heavily reduced in scincids generally, and often absent in burrowers^15,31–33^, but it is expanded in cordylids and some xantusiids^15^. The orbit is large despite the probable adult stage of the holotype (see ‘Comments on Ontogeny’ below). The prefrontal bears a long frontal process and a deep recess for the olfactory chamber. The frontals are paired, but neither their posterior sutures nor subolfactory processes are preserved. As in some lacertoid lizards, the splenial extends to the anterior fourth of the dentary and largely conceals the Meckelian canal from view (Fig. 1i), though the splenial is shorter in scincoids^15^. The posterodorsal process of the dentary is dorsally expanded to overlap the lateral ramus of the triradiate coronoid above the surangular (Fig. 1j). Ventral to the coronoid process, the dentary contacts the angular and the compound bone, i.e., the fused surangular, prearticular, and articular common among full-grown lizards^15^.

The palate is preserved in semi-articulation (Fig. 1d–h). The long, paired vomers constitute the anterior third of this region of the skull. A poorly developed Jacobson’s process delimits the posterior margin of the vomeronasal chamber housing Jacobson’s organ, and the row of three vomerine foramina is oriented mediolaterally rather than anteroposteriorly as in anguids^37,38^. The vomeronasal fossa is deep, indicating a large vomeronasal organ, but there is no clear concavity on the anterolateral margin of the vomer to accommodate the external vomeronasal fenestra. Although some iguanians, mosasaurs, gekkotans, *Varanus*, and xantusiids possess reduced margins for the vomeronasal fenestra on the vomer^15^, in none of these clades does this concavity disappear entirely. Although the vomer appears to have floored a lizard-like vomeronasal organ, †*Eoscincus ornatus* is unique among squamates in exhibiting this ancestral saurian condition—absence of a notch for the vomeronasal canal—like that retained in *Sphenodon punctatus* and extinct rhynchocephalians^39,40^.

At the posterior end of the vomer, two rows of large vomerine teeth descend from a ventrally divergent platform. This platform is in the same position as the posteroventral flange of the vomer in extant scincids (Fig. 1d, g, h). Vomerine teeth are rare and multiple vomerine tooth rows are unknown in other squamates; some individuals of the serpentiform anguid *Pseudopus apodus* bear a single row of small, recurved teeth on the vomer, but others are edentulous^17,37^. Multiple rows of vomerine teeth are present ancestrally in reptiles and retained in basal rhynchocephalians such as †*Gephyrosaurus bridensis* and †*Clevosaurus* spp., although the vomers are normally edentulous in extant *Sphenodon punctatus* (apart from a few vomerine teeth reported in some specimens^41^). Having smaller teeth on the palatine and pterygoid, and larger ones on the vomer, is unique among saurians to our knowledge^42^ and instead resembles the condition in some amphibians^43^.

Posteriorly, the vomer articulates with the elongated vomerine process of the palatine, and apparently terminates posterior to the level of the palatine-maxilla contact, which is a condition absent in iguanians^15^. The palatine articulates with the maxilla laterally, and the posteromedial section of the left palatine forms the medial border of the suborbital fenestra. Unlike the case in iguanians, in which the choanal fossa is confined to the anterior margin of the palatine, the choanal fossa runs for nearly half the length of the bone as in gekkotans and anguimorphs, but not as far posteriorly as in scincoids and lacertoids^15,29,33,44,45^. The ectopterygoid is gently bowed as in anguimorphs, snakes, scincoids, lacertoids, and gekkotans, rather than sharply angled as in iguanians^15^, rhynchocephalians^15^, and in the earliest stem squamate, the mid-Triassic †*Megachirella wachtleri*^5^. The ectopterygoid tapers to a single point anteriorly, unlike the bifid condition in Anguidae^37^ and Serpentes^15,46^. The pterygoid, preserved in articulation with the ectopterygoid, bears several small teeth. The ectopterygoid process of the pterygoid is slender and the palatine process of the pterygoid is widened.

### Systematic paleontology of †*Microteras borealis*

Squamata N. M. Oppel 1811 (de Queiroz, K. and Gauthier, J. A. 2020)

Pan-Scincoidea new taxon (See Supplementary Note 7) †*Microteras borealis* gen. et sp. nov.

#### Holotype

YPM VP 4718, closely associated maxilla, braincase, and cranial fragments belonging to the same block.

#### Locality and horizon

Como Bluff Quarry 9, Wyoming; Brushy Basin Member (Tithonian, Late Jurassic), Morrison Formation.

#### Etymology

Greek μῑκρόν (small) + τέρᾰς (marvel). Greek βορέας (north).

#### Diagnosis

**Fig. 2:**
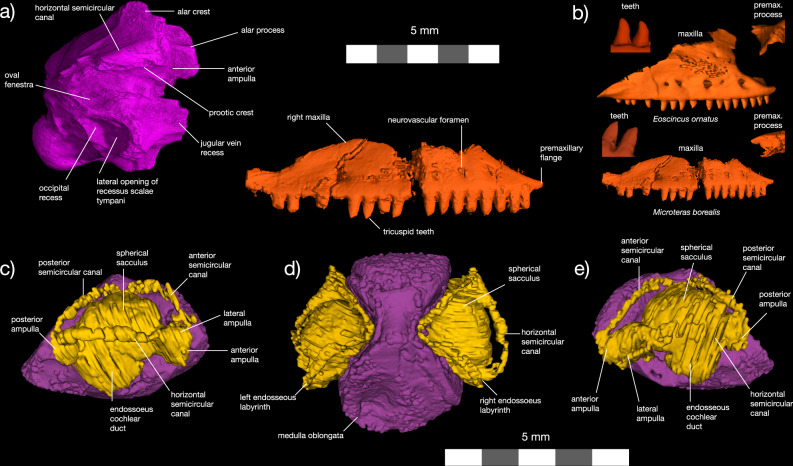
Anatomy of †*Microteras borealis* new taxon. Cranial material in (**a**) lateral view. Maxillae (**b**) of †*Microteras borealis* and †*Eoscincus ornatus* (left maxilla, reflected) compared, with insets showing their distinctive dentitions (teeth) and premaxillary process morphology (premax. process). †*Microteras borealis* possesses symmetrical, weakly tricuspid maxillary teeth, whereas the maxillary teeth of †*Eoscincus ornatus* are unicuspid and weakly recurved. Also note the medially divergent septomaxillary flange in †*Eoscincus ornatus*. Endocast in (**c**) right lateral, (**d**) left lateral, and (**e**) dorsal views. Maxilla—red, braincase—light purple, brain endocast—dark gray, inner ear—yellowish green.

†*Microteras borealis* is distinguished from other Mesozoic pan-scincoids by the following: 20+ maxillary tooth positions (19 in †*Eoscincus ornatus*); spatulate, tricuspid, homodont maxillary dentition with crowns that do not change size across tooth row (slightly heterodont, unicuspid, posteriorly recurved teeth that reduce in size posteriorly in †*E. ornatus*); septomaxillary and maxillary processes of maxilla oriented parallel to one another anteriorly (divergent in †*E. ornatus*) (Fig. 2a, b). The geographic separation of †*E. ornatus* and †*M. borealis* also supports the hypothesis that these taxa are distinct.

### Description

The holotype of †*Microteras borealis* consists of an associated maxilla and braincase (Fig. 2a). The maxilla possesses pleurodont teeth as in other lepidosaurs^39^, the dentition is homodont, and the tooth-crowns are tricuspid. This distinguishes †*M. borealis* from †*Eoscincus ornatus*, which possesses slightly heterodont, unicuspid maxillary teeth. The separated prefrontal and jugal articular facets visible on the supradental shelf of the maxilla enable identification of the holotype as a pan-scincoid^15^. †*M. borealis* also possesses much smaller maxillary neurovascular foramina than †*E. ornatus*, although size of the maxillary neurovascular foramina varies among individual extant lizard species. The maxilla of †*M. borealis* bears an anteroposteriorly long ascending ramus as in most scincoids, gekkotans, lacertoids, and anguimorphs, but unlike the anteroposteriorly shorter ramus in rhynchocephalians and iguanians^15,41^. The posterodorsal portion of the ascending ramus is not preserved.

The braincase is completely fused and lacks only the anteriormost section. Important features include a well-developed, though incomplete, alar process on the prootic (Fig. 2a). The presence of an alar process supports the placement of †*M. borealis* within Squamata apart from Iguania, which lacks this apomorphy^29^. As in lizards ancestrally, including scincoids, the braincase of †*Microteras borealis* bears prominent basal tubera (Fig. 2a)^15,47^. The shortened medulla agrees with the plesiomorphic condition for Squamata (Fig. 2c–e). The enlarged spherical sacculus, slender semicircular canals, and widened oval foramen resemble the inner ears of fossorial skinks, snakes, amphisbaenians, dibamids, anguids, and caecilians^18–20,48^. Some marine squamates possess similarly large sacculi^20^, but †*M. borealis* lived in a terrestrial environment.

### Comments on ontogeny

Following the recommendation of Griffin et al.^49^, we are providing a separate section outlining our ontogenetic assessment of both †*Eoscincus ornatus* and †*Microteras borealis*. All characters appear at some point during ontogeny, so it is important to understand ontogenetic stage to distinguish between the absence of some feature due to ontogenetic status from absence altogether during ontogeny. †*E. ornatus* lacks most of the elements that are useful for assessing squamate ontogenetic stages, such as the braincase, limbs, and girdles^50^. However, the posterior mandibular elements are preserved and the prearticular and surangular are fused, a feature common among the (mostly) full-grown squamates sampled by Gauthier et al.^15^. This fusion has been found in a complete growth series of at least one squamate species (*Aspidoscelis tigris*) to be a near-terminal ontogenetic event highly indicative of an asymptotic growth stage^51^. For this reason, we consider DINO 14684 to be a skeletally mature specimen of †*E. ornatus*.

†*Microteras borealis* preserves a braincase that is fully fused. Complete fusion of the braincase is a strong indicator of skeletal maturity in non-iguanian squamates^50^. As indicated by Petermann et al.^51^ and Petermann and Gauthier^52^, although size and age are not particularly closely coupled in squamates, several skeletal fusions are much more closely associated with near-terminal (asymptotic) body size. Complete braincase co-ossification is one of these. For this reason, we consider YPM VP 4718 to be a skeletally mature specimen of †*M. borealis*.

### Phylogenetic placement of the Morrison squamates

**Fig. 3:**
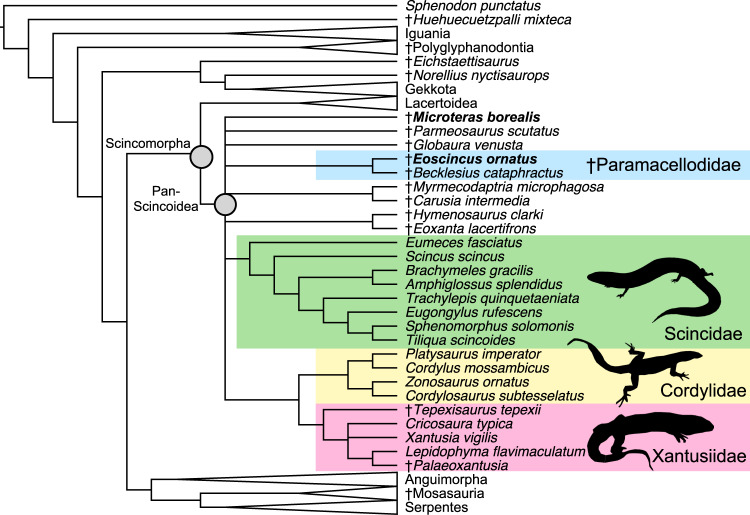
Parsimony phylogenetic analysis of the oldest definite crown squamates. Strict consensus topology generated in parsimony analysis of the morphological dataset in TNT v. 1.5. showing the positions of the two new species within Scincomorpha and Pan-Scincoidea.

To assess the phylogenetic relationships of the new species, we used an updated version of the most comprehensive—in terms of species and apomorphies sampled**—**published morphological dataset for squamates^15^ as modified by Longrich et al.^53^, with the addition of three extinct and one extant species of Pan-Lacertidae, and one species of †Paramacellodidae. The resulting strict consensus topology of 5040 trees (length = 3627, consistency index = 0.254, retention index = 0.746) generated in the parsimony analysis placed †*Eoscincus ornatus* and †*Microteras borealis* within Pan-Scincoidea (Fig. 3) and the former within †Paramacellodidae alongside the European species †*Becklesius cataphractus*. †*E. ornatus* and †*M. borealis* share with pan-scincoids a maxilla that contributes to the orbital margin (149:0), and a coronoid process of the dentary that extends dorsally to overlap the coronoid bone anterolaterally (367:1).

The strict consensus topology of 46080 trees (length = 3575, consistency index = 0.257, retention index = 0.749) found from the parsimony analysis in which we constrained the backbone clades according to the molecular topology^8^ and let individual OTUs move within clades placed †*Eoscincus ornatus* and †*Microteras borealis* in a polytomy of scincoids, lacertoids, and polyglyphanodonts. This result reflects the incompleteness of †*M. borealis*, as exclusion (most parsimonious trees = 12960, length = 3619, consistency index = 0.254, retention index = 0.745) of that species resulted in the North American †*E. ornatus* and the European species †*Becklesius cataphractus* forming a clade, the †Paramacellodidae, at the base of Pan-Scincoidea. Examination of equally parsimonious trees in the analysis including both new species (Supplementary Note 4, Supplementary Methods, Supplementary Note 6) also revealed a degree of instability in the interrelationships of some lacertoids and scincoids, which may also contribute to the polytomy in the consensus. All of these trees are figured in the Supplementary Figures and Supplementary Data File 6.

**Fig. 4:**
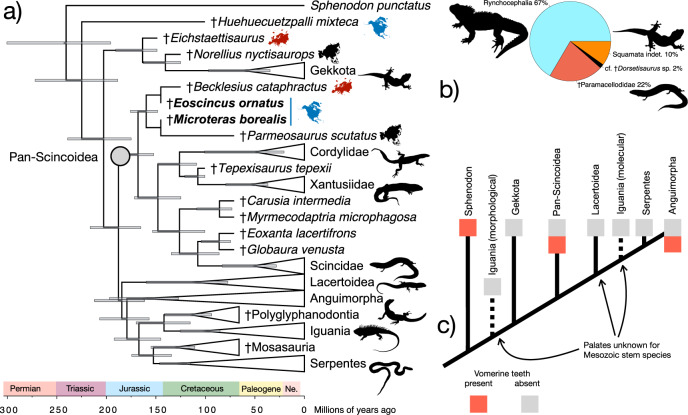
Bayesian phylogenetic analysis, biogeography, and abundance of the oldest definite crown squamates. **a** Time-calibrated maximum-clade credibility tree generated in BEAST 2.6.6. using morphological data with a backbone constraint, showing ages and distributions of early crown squamates. Blue bars represent the 95% confidence intervals for divergence dates, continent silhouettes represent distributions of key early squamates. New species bolded. **b** Relative abundance of squamates and rhynchocephalians at Como Bluff Quarry 9, the type locality of †*Microteras borealis*, based on YPM collections. **c** Distribution of vomerine teeth along lepidosaur backbone phylogeny, illustrating the possibility that the putative apomorphic nature of this feature in †*Eoscincus ornatus* may be due to insufficient sampling.

Supporting apomorphies of Pan-Scincoidea in the analysis constrained by the Burbrink et al.^8^ molecular topology include a jugal broadly separated from the prefrontal (144:0), a weak jugal median ridge (157:0), 10-20 dentary teeth (421:2), the absence of cusped posterior teeth (434:0, this is, however, present in †*Microteras borealis*), and a lightly sculptured skull roof (572:1). Bootstrap support for Pan-Scincoidea including the new species is low across all analyses (10–37%), as might be expected in incomplete, relatively unmodified, and ancient lizard fossils. Nevertheless, Bayesian analysis also placed both †*M. borealis* and †*Eoscincus ornatus* in very similar positions at the base of Pan-Scincoidea (Fig. 4a), which is estimated to have originated in the mid-Jurassic (Oxfordian) 159.8 Ma (95% CI: 148.3–175.7 Ma). Pan-Scincoidea was supported by a high posterior value of 0.98, whereas †Paramacellodidae was supported by a low value of 0.48 and crown Scincoidea was supported by a low value of 0.68. The consistent placement of the new species in Pan-Scincoidea in both Bayesian and parsimony-based phylogenetic analyses strongly supports their inclusion in this clade in spite of the low bootstrap supports found in parsimony analysis when the scant remains of †*M. borealis* are included. In sum, †*Eoscincus ornatus* and †*Microteras borealis* are known from sufficiently diagnostic material to merit their confident placement within the squamate crown (77-91% bootstrap support for crown Squamata in different analyses), share several apomorphies with pan-scincoids^15^, and are placed in Pan-Scincoidea in both analyses using morphology only and morphology with an enforced molecular constraint. Our Bayesian time-calibrated phylogeny based exclusively on the morphological dataset confirms that most squamate backbone clades diverged by the mid-Jurassic as estimated using molecular data^1,8^.

## Discussion

### Calibrating the squamate radiation in space and time

As the best-characterized pan-scincoids from the Jurassic, †*Eoscincus ornatus* and †*Microteras borealis* illuminate the early biogeography of lizards. The consistent placement of †*E. ornatus* in a clade with the Early Cretaceous European species †*Becklesius cataphractus* in Paramacellodidae provides phylogenetic evidence for a Jurassic trans-Atlantic distribution of pan-scincoids, an old hypothesis^54,55^ that was until now based on fragmentary material. Although the Early Cretaceous South American species †*Neokotus sanfranciscanus* was considered a paramacellodid by Bittencourt et al.^56^, preliminary analyses of our dataset including codings based on the fragmentary material attributed to †*N. sanfranciscanus* failed to resolve that taxon in Pan-Scincoidea (Supplementary Note 4). The distribution of North American †*E. ornatus* and †*M. borealis*, the European †*B. cataphractus*, and the Mongolian pan-scincoids †*Parmeosaurus scutatus*, †*Eoxanta lacertifrons*, †*Hymenosaurus clarki*, and †*Globaura venusta*, indicates that pan-scincoids dispersed across the Northern Hemisphere as the rifting of North America and Eurasia-Africa took place during the Late Triassic and Early-Middle Jurassic (Fig. 4a). Similar biogeographic patterns are found in contemporaneous dinosaurs^57–59^, turtles^60^, and mammaliaforms^61^, substantiating the early expansion of the Atlantic as a key driver of terrestrial vertebrate biogeography.

†*Eoscincus ornatus* and †*Microteras borealis* suggest that the initial crown squamate morphological diversification took place while the lizard sister clade, the rhynchocephalians, were the dominant small reptiles across the globe^24–26,62,63^. Both new species existed at a critical juncture in lepidosaur evolution just before the hypothesized Jurassic-Cretaceous turnovers that devastated the diversity and abundance of rhynchocephalians and paved the way for elevated squamate species richness and abundance^26,34,35,53,62–67^. At the type locality of †*M. borealis*, for example, rhynchocephalians comprise two thirds of the lepidosaur assemblage (Fig. 4b). Yet, Cretaceous lepidosaur faunas in the Northern Hemisphere are almost entirely devoid of rhynchocephalians^68^, and instead are dominated by lizards^26,34,53,68^. Consequently, the new species contribute to a growing understanding of episodes of morphological innovation in reptiles^27^. They validate that rhynchocephalians outpaced early crown squamates in richness and abundance during the first half of the Mesozoic, even as the major modern lizard clades diversified. Although squamates may have been rarer than their rhynchocephalian counterparts in the ecosystems of the Jurassic, they began early to manifest the pronounced phenotypic hallmarks of the lizard backbone clades that survived to the present day.

### The early evolution of the squamate skull

Squamate phylogenetics presents one of the greatest mysteries in vertebrate evolution: analyses of large, taxon-rich morphological^15,29,53^ and molecular^1,2,6,8,29,69,70^ datasets produce markedly different pictures of early squamate evolution^71,72^. This has profoundly obscured our understanding of the ancestral squamate skeleton and evolutionary patterns of diversification and disparification^15,29,53,72,73^.

Nevertheless, †*Eoscincus ornatus* and †*Microteras borealis* have unambiguous affinities to one of the few backbone clades with similar placement deep in the squamate crown regardless of data type: Pan-Scincoidea^1,2,6,8,29,69,70^. †*E. ornatus* and †*M. borealis* possess several hallmark squamate apomorphies, such as a widely open and deep Meckelian fossa, a large coronoid sitting atop the surangular, loss of contact between the pterygoid and vomer, fused premaxillae with a median tooth, a fused braincase and fused surangular-articular-prearticular in adults, embryonic fusion of exoccipital and opisthotic, and an alar process on the prootic^5,15^. †*E. ornatus* is anomalous in sharing two traits with Triassic stem-lepidosaurs that are lost in all extant squamates and (convergently) in *Sphenodon punctatus*: multiple vomerine tooth rows and the absence of any notch on the vomer for the external vomeronasal fenestra^5,15,17,39,40,42^.

At the same time, these conditions in †*E. ornatus* are not exactly plesiomorphic. For example, although tooth rows extend the length of the vomer in saurians ancestrally, in †*E. ornatus* they are confined to a small, ventrally deflected platform at the palatine junction, reminiscent of the shape of this suture in scincids^15^. Vomerine teeth in †*E. ornatus* are equally unusual for being larger than other palatal teeth, unlike any saurian in that respect^42^. Likewise, although unique among squamates in lacking a clear vomeronasal notch, it still has the Jacobson’s process dorsally that bounds the distinctive squamate vomeronasal organ (=Jacobson’s organ). Thus, these features, far from being retained plesiomorphies, are apomorphic evolutionary reversals diagnostic of †*E. ornatus* in this analysis. That still leaves the question of the vomerine teeth, whose presence is also inferred to be an apomorphic reversal in †*E ornatus*. However, we suspect this may be a sampling artifact, as aside from †*E. ornatus* there are no other described pre-Cretaceous squamates for which a well-preserved palate is known and younger stem-group members of several squamate backbone clades also possess vomerine teeth (Supplementary Note 4; Fig. 4c).

The new scincoids also illuminate how fossils can fill critical gaps in morphological changes first suggested by molecular phylogenetics. For example, crown lacertoids and scincoids, placed together in Scincomorpha in most morphological phylogenies, share two apomorphies in palatine morphology: loss of teeth on the palatine and a prominent choanal fossa running the length of the element^15^. The Late Jurassic stem-scincoid †*Eoscincus ornatus*, however, still has teeth on the palatine and a shorter choanal fossa, indicating that these apomorphies arose convergently in lacertoids on one hand and scincoids on the other. Although only two discordant apomorphies are involved, and Scincomorpha still emerges in analyses where only morphological data are used, that reduces the number of supporting apomorphies by 12-15% (depending on optimization). This discovery is unexpected based on morphological trees, but it is just what the molecular hypothesis would predict: because scincoids and lacertoids represent successive divergences from the lizard backbone, the morphological apomorphies they share will eventually prove convergent^1,2,6,8,29,69,70^. As illustrated here, early fossils can be expected to play a vital role in resolving such conflicts. It is not entirely satisfying to invoke morphological homoplasy to explain every disagreement with molecular trees^71^; conflicting data must ultimately be explained in their own right^5^. The new Jurassic species indicate that the way forward on this front may emerge as the earlier half of lizard evolution becomes better understood. It may be noteworthy that the sort of mosaicism early in the diversification of crown squamates exemplified by the new Morrison lizards has also been invoked to explain complex patterns of evolution during the early radiations of crown mammals^74^ and near-crown birds^75–77^. When considered alongside †*M. borealis*, which possessed tricuspid teeth and an inner ear morphology like that of some extant burrowing squamates^18–20^, †*E. ornatus* substantiates an underappreciated degree of morphological variability among early crown lizards.

## Methods

### Permits

No permits were required for this study. All specimens examined are held in public repositories.

### Abbreviations

DINO: Dinosaur National Monument YPM: Yale Peabody Museum of Natural History.

### Computed Tomography

Given the small size and fragility of the skulls we report, we opted to use high-resolution computed tomography to investigate the anatomy of the specimens in detail. DINO 14684, the holotype of †*Eoscincus ornatus*, was scanned at the University of Texas at Austin, whereas YPM VP 4718, the holotype of †*Microteras borealis*, was scanned at Yale University. Individual cranial elements were digitally segmented and prepared in 3DSlicer^78^ and VGStudio MAX 3.5. All 3D stl files of segmented bones generated are included in the Supplementary Data Files 1, 2.

### Phylogenetic Dataset

To test the relationships of †*Eoscincus ornatus* and †*Microteras borealis* within Squamata, we added them to an updated version of the morphological dataset of Gauthier et al.^15^ that included K/Pg-boundary species from the Western Interior of North America assembled by Longrich et al.^53^. Some species were deleted from this dataset for the purposes of the current analysis, including many iguanians and caenophidian snakes, but in each case retaining enough taxa to confidently bracket ancestral states for the squamate backbone clades. We also deleted wildcard fossorial species (apart from snakes) that have for centuries obscured lizard morphological systematics and to which the new taxa show no close phylogenetic affinities (see Supplementary Methods). We added recently published codings for four species in Pan-Lacertidae, three extinct (†*Eolacerta robusta*, †*Stefanikia siderea*, †*Cryptolacerta hassiaca*) and one extant (†*Gallotia galloti*) using published computed tomography scans and osteologies^79–82^, and we included one of the best known paramacellodids, †*Becklesius cataphractus*, based on the literature^33^. We limited coding to single articulated/closely associated specimens (holotypes, when available) for the newly added fossil species. This stricture is intended to reduce coding errors introduced by potentially erroneous assignments of disassociated remains to the hypodigm of a single species, which has become an issue for some Jurassic squamate faunas^10^.

Additionally, we addressed several errors in the original Gauthier et al.^15^ matrix identified by Simões et al.^11^ and Simões et al.^83^. We modified codings for xantusiids and cordylids from state 1 to state 2 to correct the coding error in accordance with Simões et al.^83^ for character 367 (large dentary-coronoid process overlaps anterolateral face of coronoid bone). The anatomy of the dentary–coronoid articulation on the lateral side of the mandible has been a pivotal character for scincoids since Estes^84^. We also recoded species for character 179 (presence of a quadrate suprastapedial process) to state 1, as all other taxa in the matrix lacking a suprastapedial process were coded as state 1 in Gauthier et al.^15^ despite this being the opposite of what was specified in the character description^11^. Because we verified the original observations, we did not change codings for character 101 in lacertids, 367 and 369 in acrodontan iguanians, 372 in rhynchocephalians, and 437 in polyglyphanodonts (*contra* Simões et al.^83^). We agree with Simões et al.^11^ that character 381, reduction of the posterior extent of the angular, cannot be coded for species in which this bone is absent, and previously corrected the mistaken codings in Longrich et al.^53^. Finally, for character 383, degree of medial exposure of the angular bone on the mandible, because *Bipes* retains a partly fused angular, these codings were not changed.

### Parsimony analyses

We first ran multiple phylogenetic analyses using different versions of our morphological dataset using parsimony in the program TNT v. 1.5^85^. The general protocol was the same for each run: *Sphenodon punctatus* was specified as an outgroup, an initial Wagner search with default parameters for ratchet, tree fuse, drift, and sectorial search was conducted with space for 1000 replicates. Finally, a round of traditional bisection-reconnection (TBR) branch swapping with more than 100,000 replicates was run to explore tree islands more fully. We ran phylogenetic analyses on the dataset both with and without a loose molecular constraint for the backbone topology found by a recent phylogenomic analysis^8^. Tests using ordered characters following Gauthier et al.^15^ produced the same topologies as unordered analyses. We found that including four non-fossorial wildcard taxa (†*Eichstaettisaurus*, †*Norellius nyctisaurops*, †*Carusia intermedia*, and †*Myrmecodaptria microphagosa*) in the analysis using the molecular constraint caused the molecular constraint to collapse, limiting our ability to test how forcing morphological characters to conform to the phylogenomic hypothesis of squamate interrelationships modified the position of †*Eoscincus ornatus* and †*Microteras borealis*. Pruning these four wildcards from the dataset allowed the molecular constraint to remain intact. We also ran a subsequent analysis excluding †*M. borealis* to test the effect of removing this additional wildcard on the placement of †*E. ornatus*. All input datasets and resulting trees are in the Supplementary Figures, Supplementary Data File 3.

### Bayesian analyses

We ran a Bayesian analysis of the morphological dataset and associated age dates taken from the literature in the program BEAST 2.6.6.^86^ under the fossilized birth-death model^87^. Two independent runs of the analysis were conducted over 1 × 10^8^ generations with a 1 × 10^7^ generation pre-burn-in using the relaxed log-normal clock model with standard values of 1.0 for the mean and 0.333 for the standard deviation. A monophyletic MRCA prior was added for Squamata to set *Sphenodon punctatus* as the outgroup as in the parsimony analyses. The Markov-variable (Mk_v_) model of character state evolution presented by Lewis^88^ was employed, and characters were partitioned into four sets based on the number of possible states as follows: 385 two-state, 146 three-state, 64 four-state, and 41 five-state characters. The resulting log file was checked using Tracer v. 1.7.2^89^ to ensure convergence. The set of posterior tree topologies produced from both analyses were then combined using LogCombiner v. 2.6.6 and summarized in a maximum-clade-credibility tree using TreeAnnotator v. 2.6.4 with median node heights.

We performed a second Bayesian analysis under the same parameters and additional priors that reflect the hypothesis for major extant squamate clade relationships found using molecular data^1–8^. Major divergences found in Burbrink et al.^8^ including Episquamata, Laterata, and Toxicofera, were input as monophyletic MCRA priors, with fossil taxa placed in these broad bins based on their positions relative to extant clades in the first run. This allowed for a rigorous test of the relationships of the new taxa in Pan-Scincoidea. All xml files used for the Bayesian analyses, as well as resulting tree, log, and state files, are included in Supplementary Data Files 4, 5.

### Lepidosaur abundance

To provide a preliminary appraisal of the relative abundance of the primary lepidosaur clades in the ecosystems inhabited by the two new species, we queried the YPM database for taxa assigned to rhynchocephalians and squamates from the Como Bluff Quarry (the type locality for †*Microteras borealis*). We could not perform a similar assessment for DNM site 412, as the holotype of †*Eoscincus ornatus* is the only lepidosaur specimen reported from that locality. The number of specimens assignable to squamates and rhynchocephalians from Como Bluff Quarry 9 are summarized in the pie chart in Fig. 3. That two thirds of the lepidosaurs from this locality are rhynchocephalians is consistent with previous studies documenting high rhynchocephalian diversity and relative abundance in Triassic-Jurassic lepidosaur faunas^24–27,90^, in stark contrast to the overwhelming dominance of squamates, and paucity of rhynchocephalians^68^, at middle latitudes in the Northern Hemisphere from the Cretaceous to Recent.

### Nomenclatural acts

This published work and the nomenclatural acts it contains have been registered in ZooBank, the proposed online registration system for the International Code of Zoological Nomenclature (ICZN). The ZooBank LSIDs (Life Science Identifiers) can be resolved and the associated information viewed through any standard web browser by appending the LSID to the prefix “http://zoobank.org/”. The LSIDs for this publication are: urn:lsid:zoobank.org:pub:3797A82E-A2F6-4E9A-87CB-9B59DB20A2A3 (publication), urn:lsid:zoobank.org:act:FE358DA4-F1CE-4DD9-A71E-C9756B147065 (†*Eoscincus*), urn:lsid:zoobank.org:act:9848A4D2-EF9B-4353-A262-199AE13E6F62 (†*Eoscincus ornatus*), urn:lsid:zoobank.org:act:11B3B6F9-5AB8-46E9-9BA7-EEE7545AF147 (†*Microteras*), urn:lsid:zoobank.org:act:280D07D0-A11F-4C67-BD85-032D06FC21EF (†*Microteras borealis*).

### Reporting summary

Further information on research design is available in the Nature Portfolio Reporting Summary linked to this article.

## Supplementary information

Supplementary Information

Description of Additional Supplementary Files

Supplementary Data 1

Supplementary Data 2

Supplementary Data 3

Supplementary Data 4

Supplementary Data 5

Supplementary Data 6

Reporting Summary

## Data Availability

All data from this paper are available in the main text and Supplementary Information files of this article. Segmented scans are available in Supplementary Data 1, 2, data for the phylogenetic analyses is available in Supplementary Data 3–5, and additional anatomical description is present in the Supplementary Text, along with locality and age data for species included in our phylogenetic analyses. All specimens are deposited in public repositories in the US, and no permits were required to scan them. Access to the holotype of †*Eoscincus ornatus* was provided to the Assembling the Tree of Life study team by Dinosaur National Park collections staff, and access to the Yale Peabody Museum collections was facilitated by J.A.G.

## Code availability

No new code was used for this paper. We direct the reader to the TNT wiki (http://phylo.wikidot.com/tntwiki) and BEAST 2 (https://www.beast2.org/) websites for information on how to conduct the phylogenetic analyses presented in this paper.
