# Supplementary material for: Evolutionary origins of the prolonged extant squamate radiation
Source: Nat Commun. 2022 Nov 29;13:7087. doi: 10.1038/s41467-022-34217-5 (PMC9708687; doi:10.1038/s41467-022-34217-5)
Supplement: Supplementary file 1 — Supplementary Information [file 41467_2022_34217_MOESM1_ESM.pdf]

Supplementary Text To: Evolutionary origins of the prolonged extant squamate radiation

Chase D. Brownstein, [1,2*] Dalton Meyer, [3] Matteo Fabbri, [4] Bhart-Anjan S. Bhullar, [3] Jacques A. Gauthier [3]

[1]Department of Ecology and Evolutionary Biology, Yale University, New Haven CT

[2]Stamford Museum and Nature Center, Stamford, CT; email: chasethedinosaur@gmail.com

[3]Department of Earth and Planetary Sciences, Yale University, New Haven, CT

[4]Negaunee Integrative Research Center, Field Museum of Natural History, Chicago, IL

[5]Yale Peabody Museum, Yale University, New Haven CT

Contents:

I. Supplementary Note 1: Abbreviations.

II. Supplementary Note 2: Computed Tomography Scan Information.

III. Supplementary Figures.

IV. Supplementary Note 3: Geological Setting.

V. Supplementary Note 4: Extended Description.

VI. Supplementary Methods.

VII. Supplementary Discussion.

VIII. Supplementary References.

IX. Supplementary Note 6: Extended Apomorphy list.

X. Supplementary Note 7: Extended Taxonomic Notes.

I. Abbreviations.

AMNH FARB—American Museum of Natural History Fossil Amphibian, Reptile, and Bird Collection, New York, USA.

DINO—Dinosaur National Monument Collection, Vernal, Utah, USA.

IGM—Mongolian Institute of Geology, Ulaanbataar, Mongolia.

NHMUK: Natural History Museum, London, United Kingdom.

YPM VP—Vertebrate Paleontology Collections, Yale Peabody Museum, New Haven, Connecticut, USA.

II. Computed Tomography Scan Information.

The holotype of †*Eoscincus ornatus* DINO 14864 (partial skull including rostrum, skull roof, palate, and mandibles) was scanned by Matthew W. Colbert in September 2007 and segmented by Jessica A. Maisano at the University of Texas at Austin and Chase Brownstein at Yale University. Parameters were: 180 kV, 0.13 µA, no filter, 1400 projections, 2 frames per projection.

The holotype of †*Microteras borealis* YPM VP 4718 (maxilla and braincase) was scanned by Dalton Meyer in April 2021 at Yale University and segmented by Dalton Meyer and Chase Brownstein at Yale University. Parameters were: 82 kV, 0.079 µA (braincase) and 0.78 mA (maxilla), no filter, 3141 projections, 2 frames per projection.

III. Supplementary Figures.

These figures can also be found in higher resolution in Supplementary Data 6 where they are numbered in the bottom right.

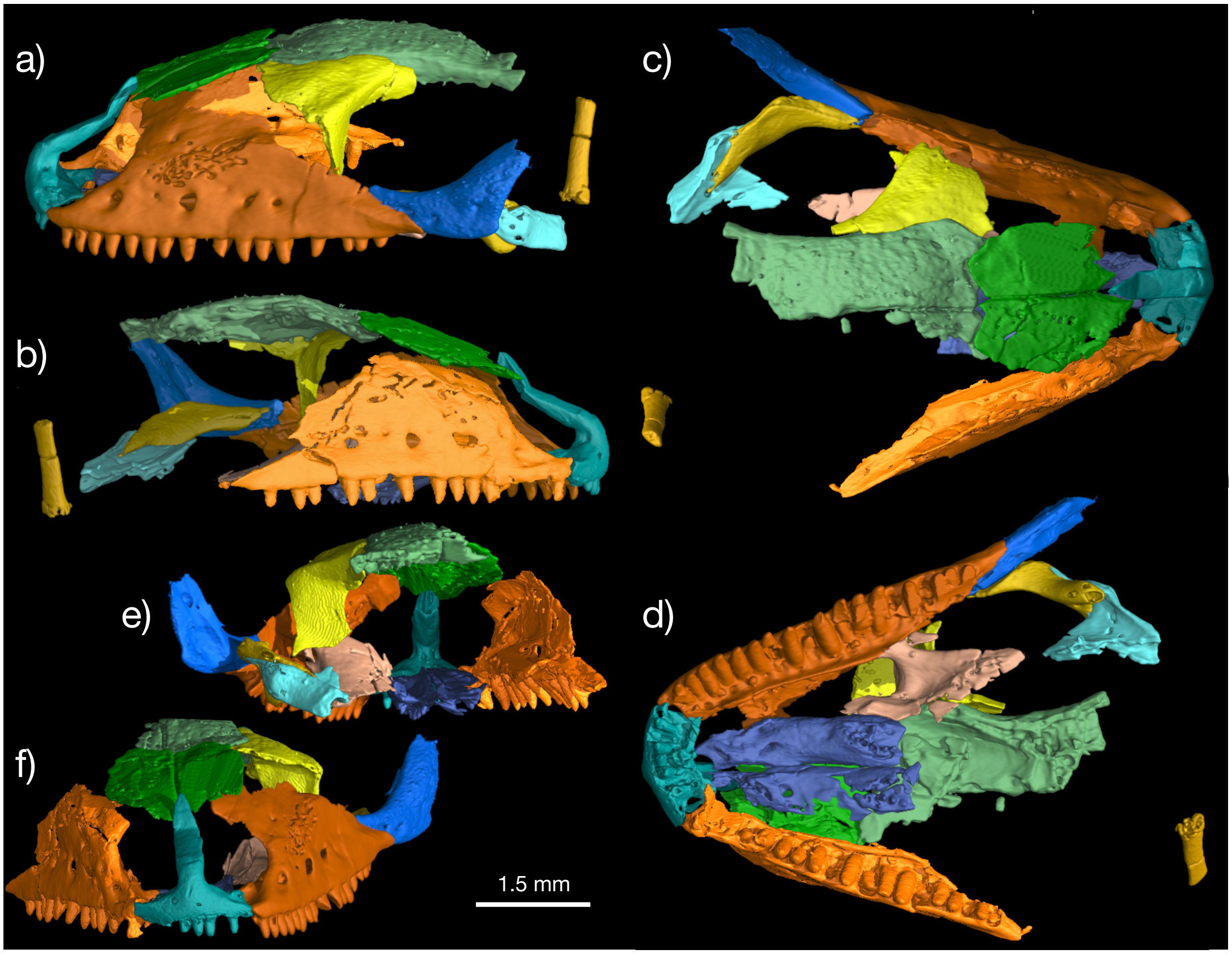

Supplementary Figure 1. †*Eoscincus ornatus* (holotype DINO 14864). Reconstructed skull in (A) left lateral, (B) right lateral, (C) dorsal, (D) ventral, (E) posterior, and (F) anterior views. Colors are the same as in the main text.

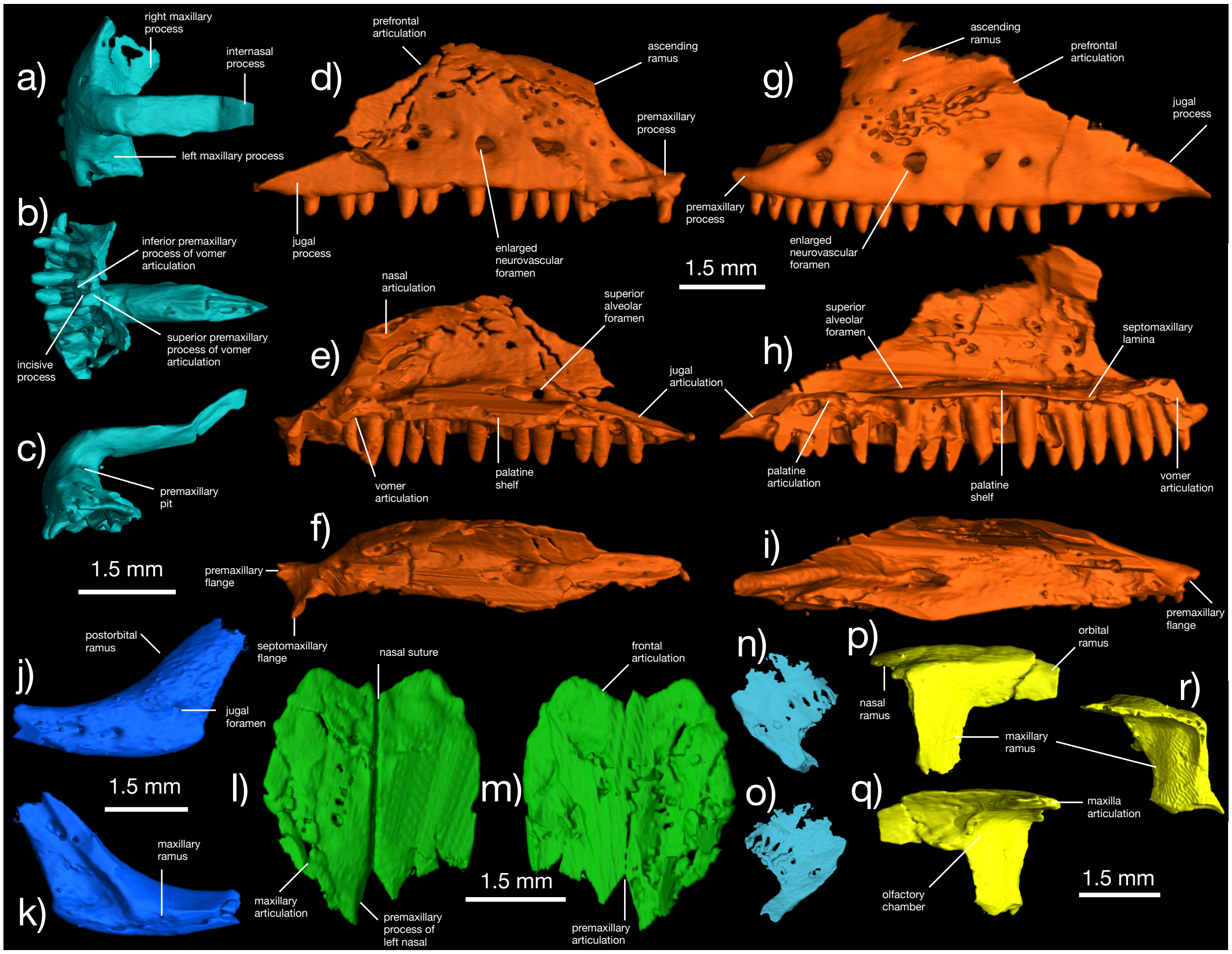

Supplementary Figure 2. †*Eoscincus ornatus* (holotype DINO 14864), facial bones. Premaxilla in (A) dorsal, (B) ventral, and (C) left lateral views. Right maxilla in (D) lateral, (E) medial, and (F) dorsal views. Left maxilla in (G) lateral, (H) medial, and (I) dorsal views. Left jugal in (J) lateral and (K) medial views. Nasals in (L) dorsal and (M) ventral views. Left ?palpebral in (N) dorsal and (O) ventral views. Left prefrontal in (P) lateral, (Q) medial, and (R) anterior views. Colors are the same as in the main text.

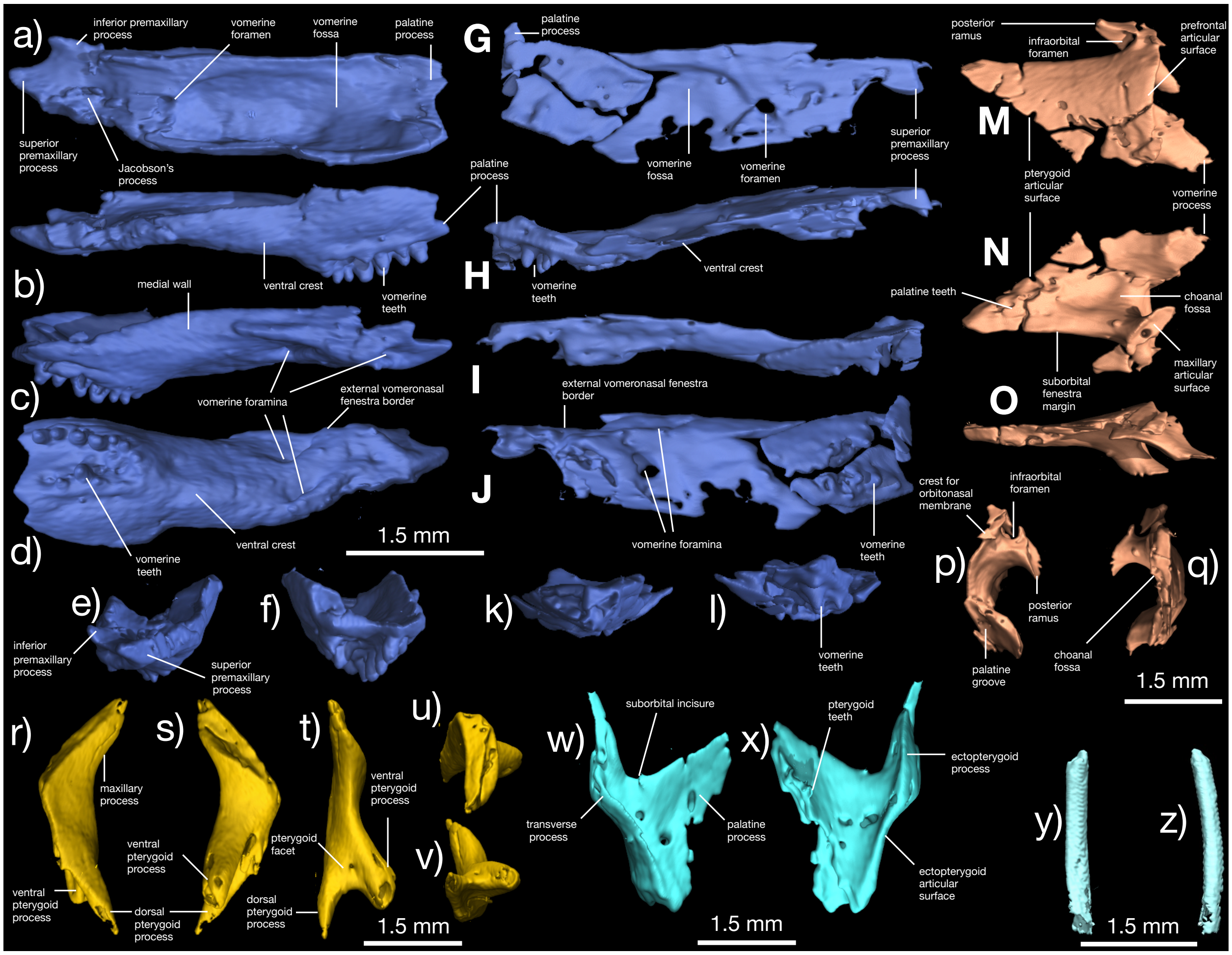

Supplementary Figure 3. †*Eoscincus ornatus* (holotype DINO 14864), palate. Left vomer in (A) dorsal, (B) lateral, (C) medial, (D) ventral, (E) anterior, and (F) posterior views. Right vomer in (G) dorsal, (H) lateral, (I) medial, (J) ventral, (K) anterior, and (L) posterior views. Left palatine in (M) dorsal, (N) ventral, (O) lateral, (P) anterior, and (Q) posterior views. Left ectopterygoid in (R) dorsal, (S) ventral, (T) medial, (U) anterior, and (V) posterior views. Left anterior pterygoid in (W) dorsal and (X) ventral views. First ceratobranchial fragment? in (Y) ventral? and (Z) lateral? views. Colors are the same as in the main text.

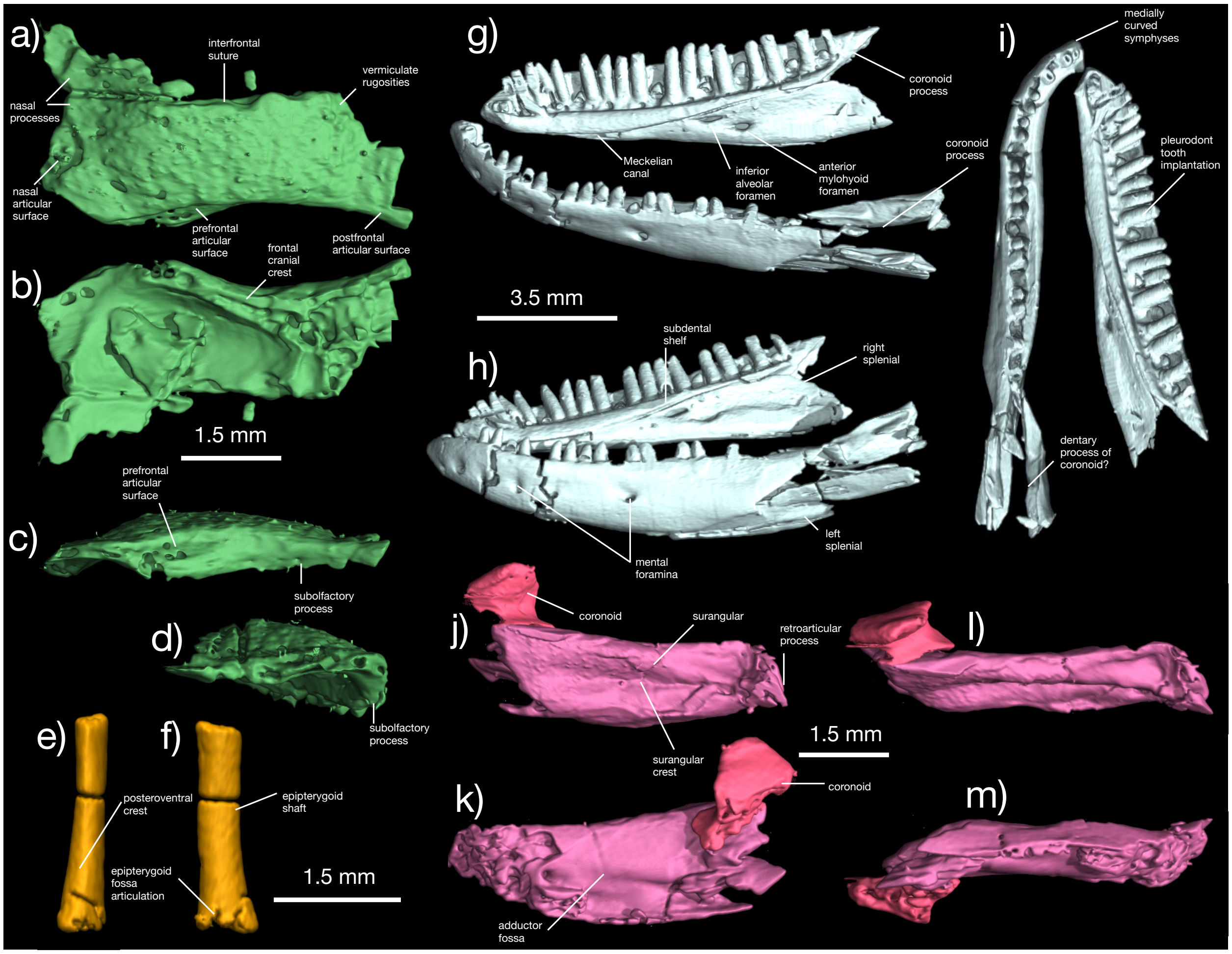

Supplementary Figure 4. †*Eoscincus ornatus* (holotype DINO 14864), skull roof, postorbital skull, and mandibles. Frontals in (A) dorsal, (B) ventral, (C) left lateral, and (D) anterior views. Right epipterygoid in (E) lateral and (F) medial views. Dentaries in (G) left dorsolateral, (H) left lateral, and (I) dorsal views. Left coronoid and fused postdentary elements in (J) lateral, (K) medial, (L) dorsal, and (M) ventral views. Colors are the same as in the main text.

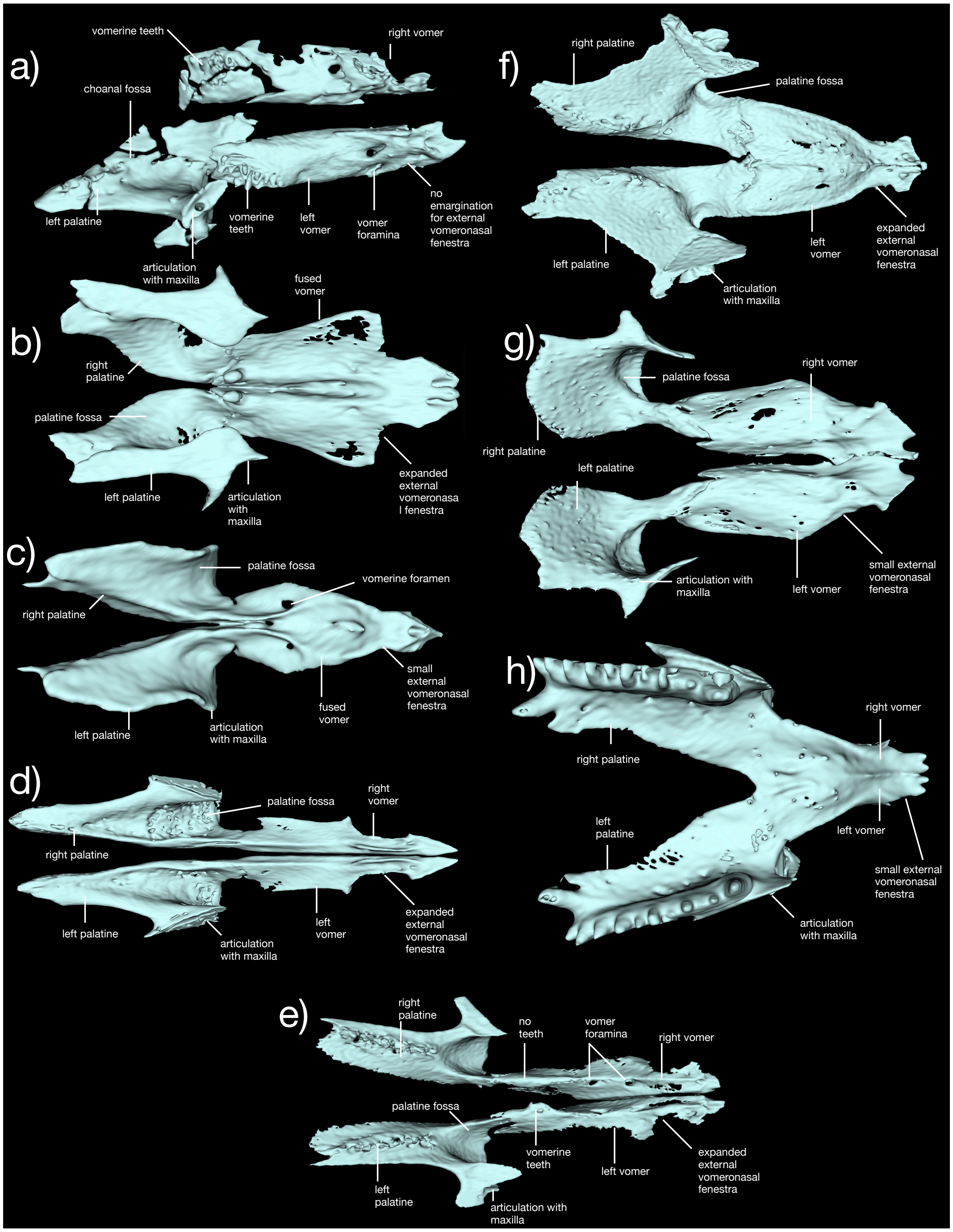

Supplementary Figure 5. Comparison of the palates of select squamate clades. Palatines and vomers of (A) †*Eoscincus ornatus* (holotype DINO 14864; Pan-Scincoidea), (B) *Eumeces fasciatus* (Scincoidea, Scincidae), (C) *Lepidophyma flavimaculatum* (Scincoidea, Xantusiidae), (D) *Tupinambis teguixin* (Lacertoidea, Teiidae), (E) *Pseudopus apodus* (Anguimorpha, Anguidae), (F) *Gambelia wislizenii* (Iguania, Crotaphytinae), (G) *Coleonyx variegatus* (Gekkota, Eublepharidae), and (H) *Sphenodon punctatus* (Rhynchocephalia).

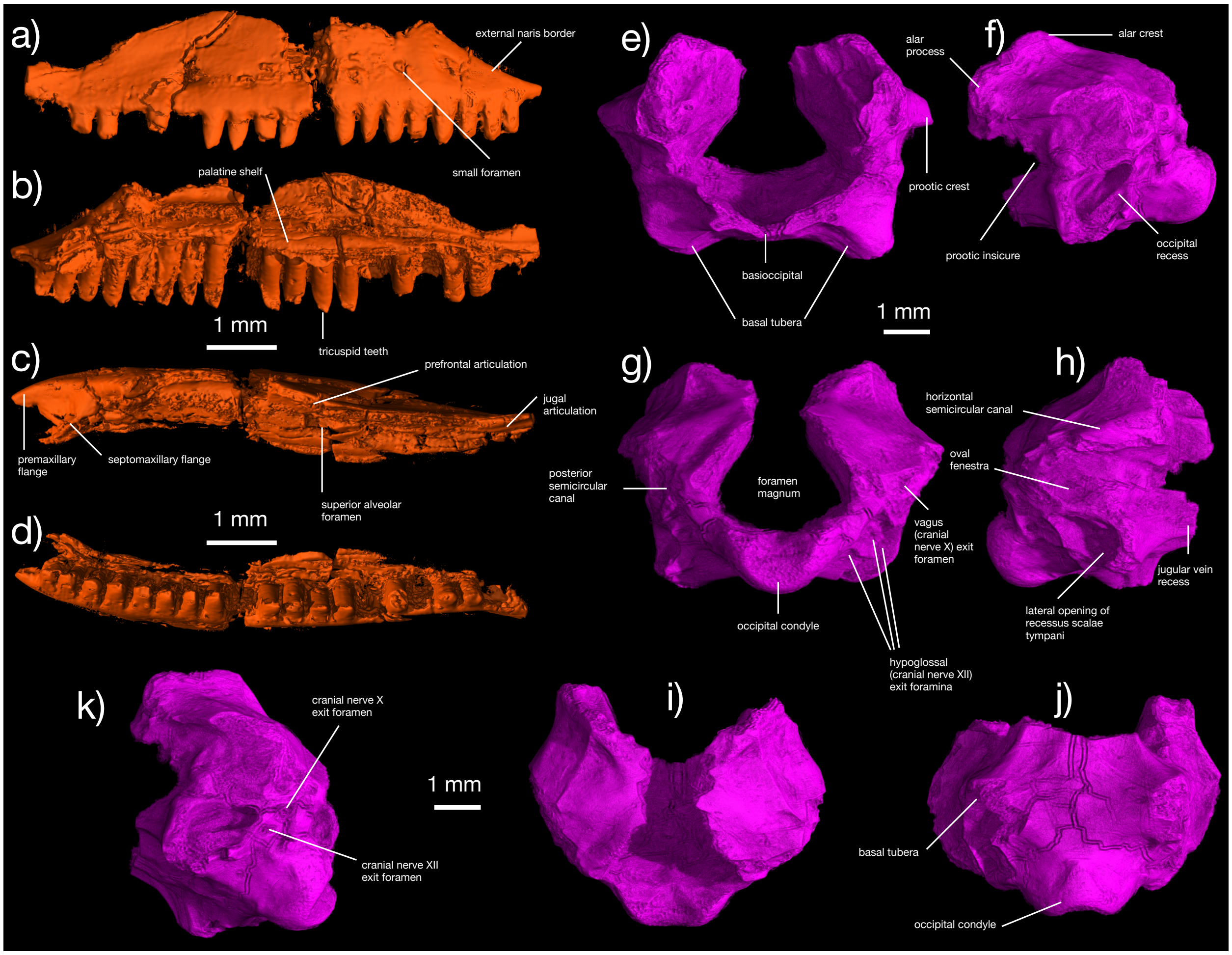

Supplementary Figure 6. †*Microteras borealis* (holotype YPM 4718), maxilla and braincase. Right maxilla in (A) lateral, (B) medial, (C) dorsal, and (D) ventral views. Braincase in (E) anterior, (F) left lateral, (G) posterior, (H) right lateral, (I) dorsal, (J) ventral, and (K) left posterolateral views. Colors are the same as in the main text.

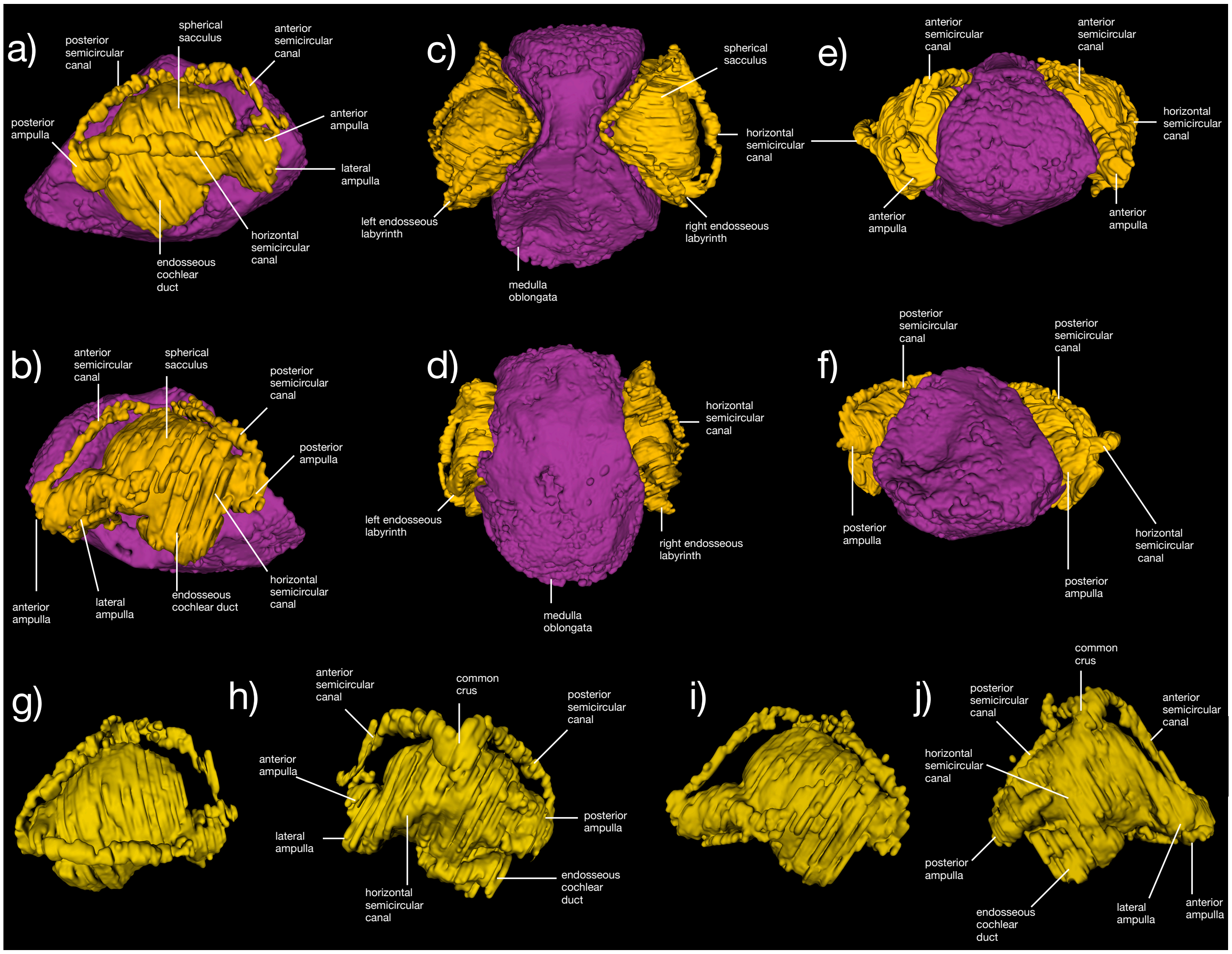

Supplementary Figure 7. †*Microteras borealis* (holotype YPM 4718), endocast. Medulla and inner ears in right lateral (A), left lateral (B), dorsal (C), ventral (D), anterior (E), and posterior (F) views. Right inner ear in (G) lateral and (H) medial views. Left inner ear in (I) lateral and (J) medial views. Colors are the same as in the main text.

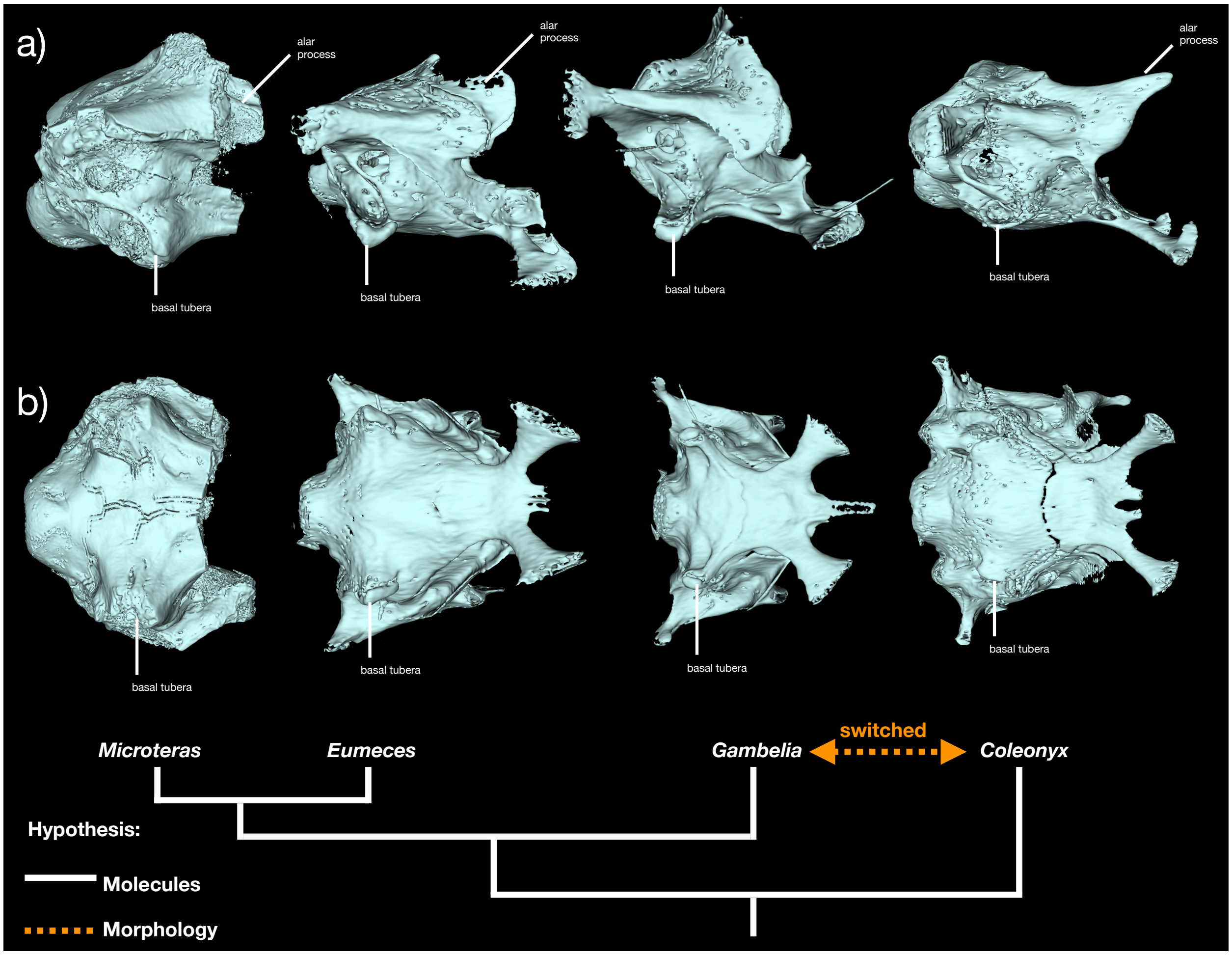

Supplementary Figure 8. Comparative braincase anatomy of *Microteras borealis* (holotype YPM 4718). Select squamate braincases in (A) right lateral and (B) ventral views.

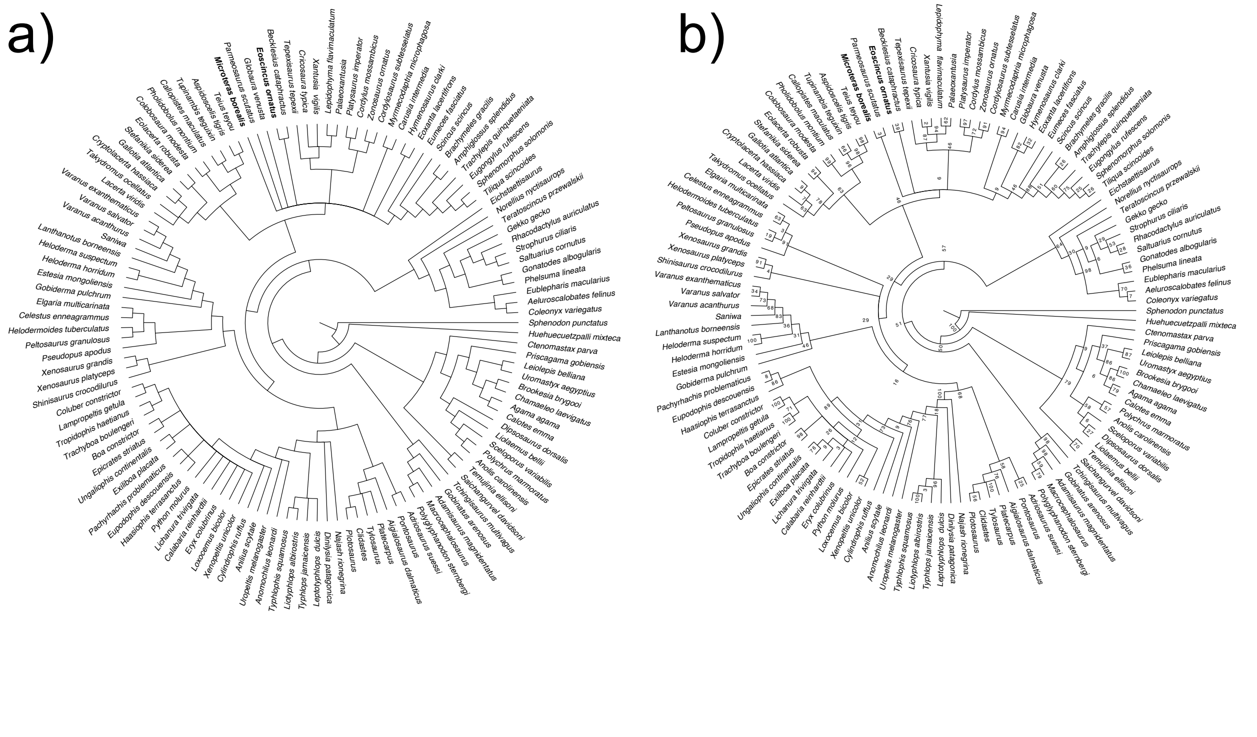

Supplementary Figure 9. Topologies produced in the unconstrained parsimony analysis. (A) strict consensus topology, and (B) resampled tree with bootstrap supports.

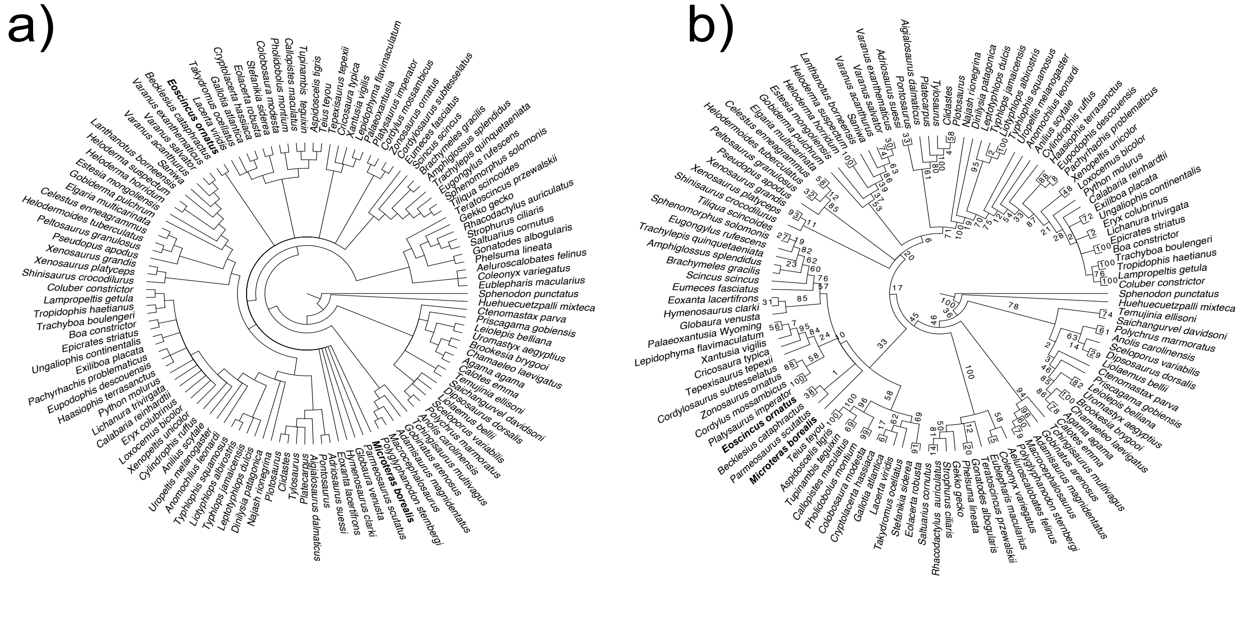

Supplementary Figure 10. Topologies produced in the constrained parsimony analysis. (A) strict consensus topology and (B) resampled tree with bootstrap supports.

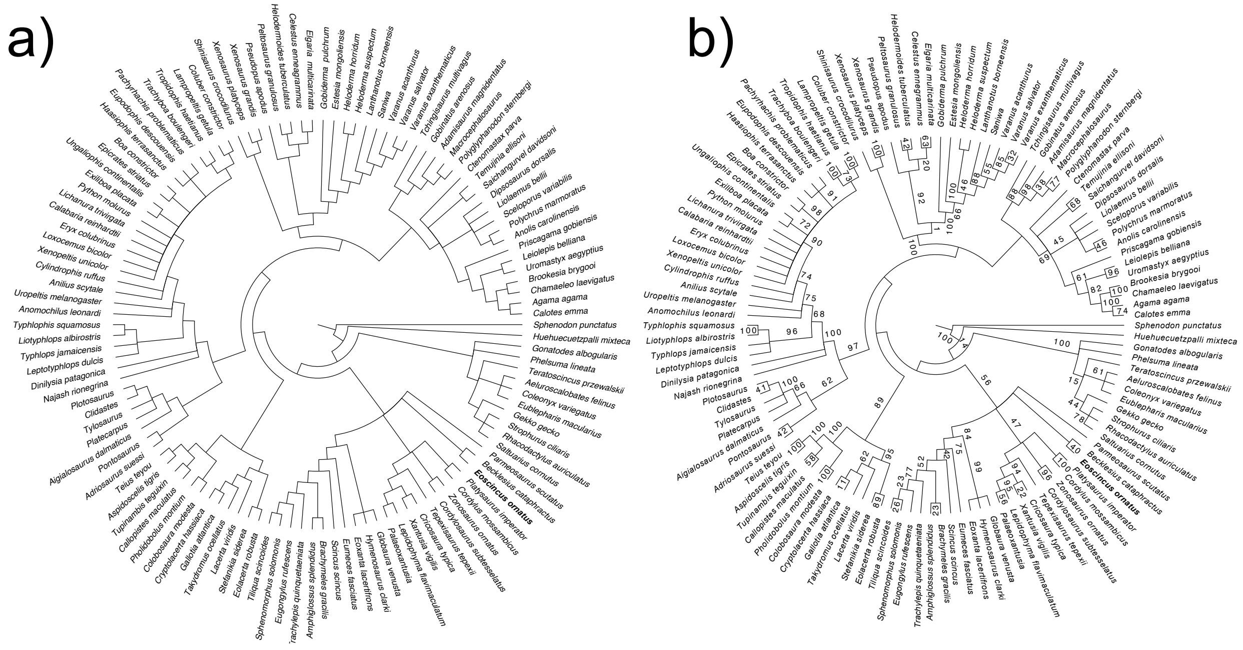

Supplementary Figure 11. Topologies produced in the constrained parsimony analysis. (A) strict consensus topology and (B) resampled tree with bootstrap supports from the analysis including only †*Eoscincus ornatus*.

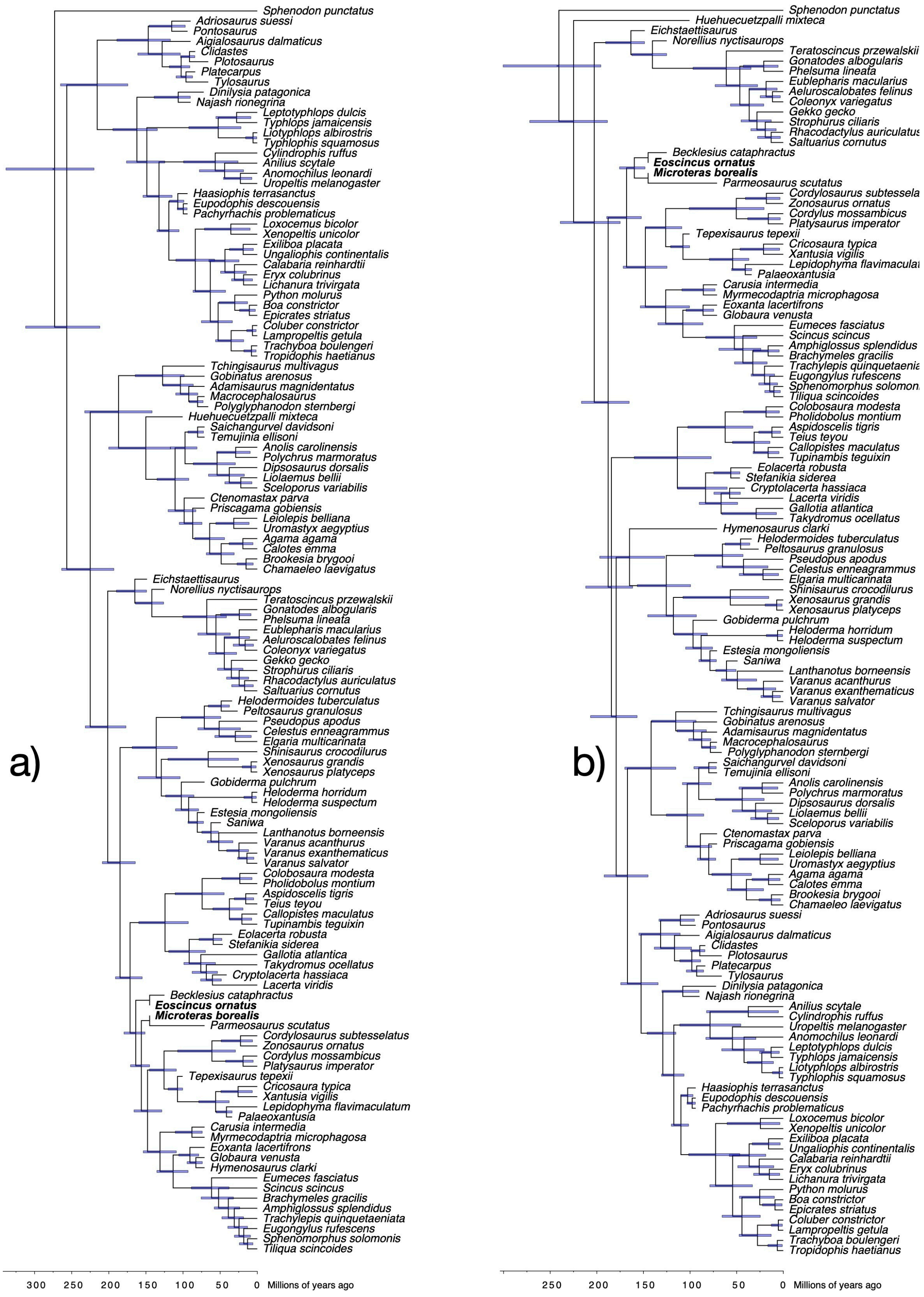

Supplementary Figure 12. Bayesian maximum clade credibility trees. (A) MCC tree using only morphology. (B) MCC tree from analysis with molecular constraints enforced.

IV. Geological Setting.

The holotype of †*Eoscincus ornatus* comes from the DINO 317 quarry in easternmost Utah, which corresponds to the Brushy Basin Member of the Morrison Formation (Late Jurassic; Evans and Chure, 1997, 1998). The Morrison Formation is a widespread unit in western North America notable for producing a massive collection of large-bodied dinosaur fossils (e.g., Foster, 2003; Turner and Peterson, 2004). Numerous fossil plants, arthropods such as insects and ostracods, mollusks such as bivalves and gastropods, and diverse vertebrates including chondrichthyans, actinopterygians, dipnoans, amphibians, and several small-bodied reptiles and mammals have also been described from Morrison Formation exposures (e.g., Ash and Tidwell, 1998; Kirkland, 1998; Gorman et al., 2008; Smith et al., 2011; Lara et al., 2020).

The Morrison Formation was created by the final regression of the transcontinental Sundance Seaway during the beginning of the Sevier orogeny in the Late Jurassic (e.g., Cooley and Schmidt, 1998; Currie, 1998; DeCelles, 2004; Bertog et al., 2014; Christiansen et al., 2015; Maidment and Muxworthy, 2019). The Brushy Basin Member (BBM) is the uppermost unit of the Morrison Formation and is usually dated to the Tithonian Stage of the Late Jurassic (153—145 Ma, Kowallis et al., 1991; Kowallis et al., 2007; Tanner et al., 2014; Trujillo et al., 2014; Christiansen et al., 2015). The BBM represents fluvial-lacustrine ecosystems and includes sites interpreted as point-bars, lags, and oxbow lakes (Turner and Peterson, 2004; Bertog et al., 2014; Tanner et al., 2014). In the region of Dinosaur National Monument, BBM exposures primarily derive from the lower half of the unit and consist of reddish-brown mudstone and thick sandstone interspersed with thinner sandstone, volcanic tuff, and limestone (Tanner et al., 2014). Paleosols in the lower BBM are moderately calcareous and commonly possess carbonate nodules (Tanner et al., 2014).

In the region where †*Eoscincus ornatus* was collected, the BBM has produced material from at least three additional species of squamates representing two or more major squamate backbone clades (e.g., Evans and Chure, 1998, Evans and Chure, 1999). These include DINO 15914, a specimen that was originally attributed to †*Paramacellodus* sp. by Evans and Chure (1998), a relationship not recovered by our preliminary analyses (Meyer et al., in prep). A single dentary, DINO 15915, was interpreted as a record of the Early Cretaceous anguimorph (Conrad, 2008) †*Dorsetisaurus* by Evans and Chure (1999). †*Schilleria utahensis*, represented by a disarticulated partial skull and skeleton (DINO 14720), may be a stem squamate (Evans and

Chure, 1999). Farther from the locality where †*E. ornatus* was collected, the BBM in Colorado has produced the remains of the possible scincoid †*Saurillodon* and the problematic †*Parviraptor*, which has variously been interpreted as an anguimorph (Evans, 1996), a stem gekkotan (Daza et al., 2013), or as a stem snake (Caldwell et al., 2015).

YPM 4718, the holotype of †*Microteras borealis*, was collected from Brushy Basin exposures in Quarry 9 of Como Bluff, Albany County, Wyoming. The specimen includes two closely associated blocks, one holding the braincase and the other holding the maxilla. The squamate fauna from this quarry was preliminarily reported by Prothero and Estes (1980), who noted the presence of "paramacellodids" and a dentary that compared favorably with the Early Cretaceous European species †*Dorsetisaurus purbeckensis*.

V. Extended Description.

<u>Eoscincus ornatus.</u>

†*Eoscincus ornatus* (Fig. S1—S5) was formerly considered a specimen of †*Paramacellodus* cf. *oweni* (Evans and Chure, 1998). Although we agree that referred material of †*Paramacellodus oweni* does bear resemblance to †*E. ornatus*, the holotype of †*P. oweni* (a pterygoid, mandibles, and several vertebrae) possesses a pterygoid with ectopterygoid and palatine processes diverging at a much greater angle (~90° degrees vs. ~60° degrees in †*E. ornatus*; Fig. S3W—X) and more slender dentaries (see Hoffstetter, 1967). Given these morphological differences and the wide geographic and temporal separation between †*P. oweni* and †*E. ornatus*, we propose a new name for the taxon represented by the nearly complete Dinosaur National Monument skull.

***Premaxilla.*—**The premaxillae (Fig. S1, S2A—C) are fused into a single element in †*Eoscincus ornatus*, unlike the paired condition ancestral for Scincidae (e.g., Greer, 1970; Nash and Tanner, 1970; Estes et al., 1988; Hutchinson et al., 1989; Caputo, 2004; Gelnaw, 2011; Gauthier et al., 2012; Paluh and Bauer, 2017; Camaiti et al., 2019; Thorn et al., 2021). The premaxillae are also paired in the putative stem-scincids of the Mongolian Cretaceous, i.e., †Globauridae (*sensu* Gauthier et al., 2012 = †*Globaura venusta* Borsuk-Białynicka, 1988 + †*Eoxanta lacertifrons* Borsuk-Białynicka, 1988 + †*Hymenosaurus clarki* Gao and Norell, 2000) and †Carusiidae (sensu Gauthier et al., 2012 = †*Carusia intermedia* Borsuk-Białynicka, 1988 + †*Myrmecodaptria microphagosa* Gao and Norell, 2000). The Late Jurassic pan-gekkotan (Gauthier et al., 2012; Simões et al., 2017; Simões et al., 2018) or pan-squamate (Conrad, 2015) †*Eichstaettisaurus schroederi* possesses paired premaxillae (Simões et al., 2017). Fused premaxillae are, however,

found in Cordylidae + Xantusiidae, the sister to Scincidae among extant squamates (Gauthier et al., 2012; Burbrink et al., 2020), as well as in the stem-scincoid †*Parmeosaurus scutatus* (Gao and Norell, 2000), the scincoid †*Retinosaurus hkamtiensis* (Čerňanský et al., 2022), and the possible paramacellodid stem-scincoid †*Neokotus sanfranciscanus* (Bittencourt et al., 2020). The premaxillae are also fused throughout Lacertoidea (e.g., Gao and Norell, 2000; Conrad, 2008; Gauthier et al., 2012; Tałanda, 2016; Čerňanský et al., 2017; Hernández Morales et al., 2019; Čerňanský and Syromyatnikova, 2019**;** Cruzado-Caballero et al., 2019). Lacertoidea resolves as the sister-crown to Scincoidea in some morphological trees (e.g., Conrad, 2008; Gauthier et al., 2012) but is the subsequent major divergence from the backbone of Squamata in molecular (e.g., Pyron et al., 2013; Zheng and Wiens, 2016; Burbrink et al., 2020) and combined (Reeder et al., 2015; Simões et al., 2018) phylogenies. The paired premaxillae of Scincidae have been regarded as a trait reversal rather than the retention of the paired condition ancestral for Pan-Lepidosauria (Gauthier et al., 1988), and the data presented in this paper confirm that hypothesis. The maxillary processes are long and taper slightly toward their posterolateral edges, which are squared off and would have been sutured firmly to the maxillae. The internasal process (nasal process of Thorn et al., 2021) is slender and moderately long, extending roughly one quarter of the length of the nasals to the frontals. The triangular posterior end of the internasal process is accommodated by the wedge-shaped premaxillary articulation of the nasals. The internasal process is triangular in cross-section midway along its long axis and bears a distinct ridge on its ventral surface marking where the nasals are closely apposed on the midline. Among Mesozoic pan-scincoids, only †*Parmeosaurus scutatus* shows a similarly elongated internasal process of the premaxilla, although this feature is widely distributed among other Late Cretaceous lizards (Gao and Norell, 2000; Gauthier et al., 2012; Tałanda, 2016). Premaxillary pits are situated along the external margins of the base of the internasal process, and the ethmoidal foramina exit via the incisive notch (both found in †*P. scutatus*; Gao and Norell, 2000; Gauthier et al., 2012). The ventral ectethmoidal foramina are very small. There are nine vertically oriented, pleurodont premaxillary teeth, including a central, midline tooth.

Maxilla.—The maxilla (Fig. S1, Fig. S2D—I) is large bone that comprises the majority of the anterior skull of †*Eoscincus ornatus* in lateral view. The maxilla possesses a long ascending ramus, a feature widely distributed among Jurassic and Cretaceous squamates (e.g., Evans and Chure, 1998; Gao and Norell, 2000; Evans and Wang, 2005; Conrad, 2008; Gauthier et al., 2012;

Daza et al., 2012; Daza et al., 2013; Tałanda, 2016; Simões et al., 2017; Simões et al., 2018; Bittencourt et al., 2020; Čerňanský et al., 2022). An anteroposteriorly long ascending ramus is an apomorphy arising inside squamates in morphological analyses (e.g., Gauthier, 2012), as iguanians retain the relatively narrower ramus seen among rhynchocephalians. However, in molecular analyses, in which Iguania is nested inside Toxicofera, the condition in iguanians must be regarded as an evolutionary reversal. The ascending ramus is heightened relative to most lizards, which is a putative feature of paramacellodids (Evans and Chure, 1998). The ascending rami and the premaxillary flanges form much of the posterior and ventral borders of the large external nares. The combination of a large ascending ramus and an elongated, upturned premaxillary flange was used by Evans and Chure (1998) to assign the taxon we name †*Eoscincus ornatus* to Paramacellodidae. However, the holotype specimen of †*Paramacellodus oweni* does not include a maxilla (Hoffstetter, 1967). Our updated phylogeny of Squamata demonstrates that 'paramacellodid' type maxillae with upturned premaxillary flanges (Evans and Chure, 1998, 1999; Nydam and Cifelli, 2002; Bittencourt et al., 2020) are widely distributed among early pan-scincoids. For example, some squamate maxillae from the Jurassic of England (NHMUK 8104, 8118) are similar to the maxillae of †*Eoscincus ornatus*, but there is currently little direct evidence that these specimens form a clade due to the incompleteness and dissociation of the English material. In †*E. ornatus*, the ascending rami curve medially to contact the nasals and bear moderately developed vermiculate rugosities on their lateral surfaces. Maxillary dermal rugosities are commonly found in scincoids, lacertoids, anguimorphs, and iguanians but not mosasaurs, gekkotans, or snakes, but except in the first three clades, they are seldom present on the ascending ramus of the maxilla (e.g., Gao and Norell, 2000; Folie et al., 2005; Nance, 2007; Gelnaw, 2011; Gauthier et al., 2012; Camaiti et al., 2019; Thorn et al., 2021). A row of four maxillary neurovascular foramina lies just ventral to the rugosities on the lateral surface of the maxilla. These foramina are very large and differentiate †*E. ornatus* from DINO 15914 (the other Morrison specimen referred to †*Paramacellodus* by Evans and Chure, 1998), the Purbeck fossils (Hoffstetter, 1967), Jurassic German squamates such as †*Eichstaettisaurus* and †*Ardeosaurus* (Simões et al., 2016), and Late Cretaceous Mongolian pan-scincoids (Gao and Norell, 2000). The Early Cretaceous species †*Retinosaurus hkamtiensis*, which may be a pan-scincoid and pan-xantusiid, possesses a poorly ornamented maxilla with a long, low ascending ramus (Čerňanský et al., 2022). Adult crown scincids (Greer, 1970; Nash

and Tanner, 1970; Estes et al., 1988; Hutchinson et al., 1989; Caputo, 2004; Gelnaw, 2011; Paluh and Bauer, 2017; Camaiti et al., 2019; Thorn et al., 2021), cordylids (Nance, 2007; Bates and Stanley, 2020), and xantusiids (Gauthier et al., 2012; Smith and Gauthier, 2013) lack similarly large maxillary neurovascular foramina. The posterior margin of the ascending ramus bears articular facets for the prefrontal and lacrimal. The "step-like margin" on the maxillae of †*E. ornatus* described by Evans and Chure (1998) is in fact the result of damage to the posterodorsal edge of the ascending ramus on the left maxilla. The jugal process is long and extends beneath the orbit. The articular facet for the jugal does not reach the level of the prefrontal and lacrimal articulations, demonstrating that †*E. ornatus* possesses the pan-scincoid condition of the incomplete exclusion of the maxilla from the orbital margin (e.g., Gauthier et al., 2012). †*Parmeosaurus scutatus* also shows this apomorphy (Gao and Norell, 2000) shared by all Pan-Scincoidea (Gauthier et al., 2012).

Medially, the maxillae of †*Eoscincus ornatus* possess developed palatine shelves with subtle articular surfaces for the vomer and palatine. The septomaxillary lamina is preserved as a poorly developed platform on the medial surface of the left maxilla (Fig. S2H). In both maxillae, the septomaxillary flange extends anteromedially and is laterally bordered by a web of bone extending to the premaxillary flange. The left palatine and maxilla remain articulated *in situ*, demonstrating the firm sutural connection between these bones in †*E. ornatus* (Fig. 1D, G—H). This articulation occurs anterior to the posterior end of the left vomer (Fig. S1D, S5A). This is a derived condition among squamates aside from iguanians (and *Sphenodon punctatus*), in which the posterior end of the vomer lies at or anterior to the level of the articulation between the palatine and maxilla (Fig. S5B—E; Gauthier et al., 2012). †*Parmeosaurus scutatus* also shows the derived condition (confirmed by C.D.B. from CT scans of referred specimen IGM 3/139) common to other pan-scincoids except for xantusiids and †*Carusia intermedia* (Gauthier et al., 2012). The articular surface for the lateral margin of the nasals formed by the medial edge of the maxillary ascending ramus is vertical, demonstrating that the nasal neither overlapped nor underlapped the maxilla (Fig. S2E—F). The prominent superior alveolar foramen is situated above the palatine shelf midway along the anteroposterior axis of the maxilla. The maxillary tooth count is 19. The maxillary teeth are pleurodont and unicuspid with fine striae (Fig. 1A—B; Fig. S2D—I; Evans and Chure, 1999). Anterior crowns are slightly recurved, whereas mid- and posterior maxillary crowns are more vertically disposed. The maxillary teeth of †*E. ornatus*

display the ‘paramacellodid’ crowns described by Richter (1994), with a distinct, lingually offset, narrow-based cuspule connected by a crest to the labial apex of the cutting edge of the crown. Similar dental morphologies are found in some cordylids (Kosma, 2004), and a wider set of pan-scincoid dental data will be needed to test which of these apomorphic features in tooth-crown morphology are diagnostic of †Paramacellodidae as opposed to all Pan-Scincoidea (see Thorn et al., 2021: fig. 12; and see Comments on Pan-Scincoidea in Extended Taxonomic Notes below).

Jugal.—A portion of the left jugal of †*Eoscincus ornatus* consisting of the main body and bases of the maxillary and postorbital rami is obscured by matrix but observable on CT scans (Fig. S1, S2J—K). The jugal is gently curved, with a horizontal maxillary ramus and a posterodorsally projecting postorbital ramus. The jugal bears three foramina on its lateral surface. A small spur along the posterolateral edge of the bone is identified as the quadratojugal process. The quadratojugal process is said to be absent in †*Parmeosaurus scutatus* (Gao and Norell, 2000)), but CT scans suggest that this part of the jugal may be damaged. This process is also absent in †*Neokotus sanfranciscanus* (fig. 2J in Bittencourt et al., 2020) and †*Retinosaurus hkamtiensis* (Čerňanský et al., 2022). A small quadratojugal process similar to that in †*E. ornatus* is present in extant scincids (Nash and Tanner, 1970; Hutchinson et al., 1989; Caputo, 2004; Gelnaw, 2011; Paluh and Bauer, 2017; Camaiti et al., 2019) except in those with reduced jugals (e.g., *Acontias percivali*; Gauthier et al., 2012). The same is true of the quadratojugal process in cordylids (Nance, 2007; Gauthier et al., 2012; Bates and Stanley, 2020), although it is often more prominently developed in cordyline cordylids (e.g., Estes et al., 1988: Fig. 26) than in scincids. The quadratojugal process is more developed in some fossils attributed to †*Palaeoxantusia* (Gauthier et al., 2012). It is even more prominent in others (e.g., *P. kyrentos*; Shatzinger, 1980), forming a conspicuous subtriangular process as in †*Lepidophyma*. However, the quadratojugal process is absent in the small-bodied extant xantusiid †*Cricosaura typica* and in all *Xantusia* (Gauthier et al., 2012) except for the *Lepidophyma*-sized *X. riversiana* (JAG, pers. obs.). The process is absent in iguanians (Gauthier et al., 2012), mosasaurs (Gauthier et al., 2012), and the stem-squamates †*Megachirella wachtleri* (Simões et al., 2018) and †*Huehuecuetzpalli mixteca* (Reynoso, 1998). Medially, the jugal bears a shallow maxillary articular surface dorsally demarcated by an anteroposteriorly running ridge that disappears at the junction of the postorbital and maxillary rami at the main body of the jugal. This is present in cordylids (e.g.,

Nance, 2007) and lacertoids (e.g., Hernández Morales et al., 2019; Cruzado-Caballero et al., 2019) but reduced in crown scincids (e.g., Gelnaw, 2011; Camaiti et al., 2019) and xantusiids (Gauthier et al., 2012).

***Nasal.*—**The nasals are paired, subrectangular bones in †*Eoscincus ornatus* (Fig. S1, S2L—M). They are shorter than the frontals and form the dorsal border of the external nares. Anteriorly, the nasals diverge from each other at the midline of the skull to accommodate the triangular posterior end of the internasal ramus of the premaxilla. Each nasal extends slightly anterolateral to the narial border. The nasals contact the ascending rami of the maxillae laterally and the nasal process of the prefrontal posterolaterally. The nasals lack ventral projections and nasal foramina. Posteriorly, the triangular frontal processes of the nasals extensively overlap the frontals dorsally. This is the plesiomorphic condition in squamates (Gauthier et al., 2012: Appx. 2, Fig. 32) and is generally found in Jurassic and Cretaceous taxa (e.g., Gao and Norell, 1998; 2000; Gauthier et al., 2012; Simões et al., 2017; Čerňanský et al., 2022).

?*Palpebral.*—A thin, curved bone (Fig. S2N—O) overlying the dorsal surface of the prefrontal *in situ* in the holotype of †*Eoscincus ornatus* was originally identified as the left palpebral by Evans and Chure (1998). However, re-articulation of the skull of †*E. ornatus* demonstrated that this element, if identified correctly, would have formed a massive, wing-like shelf projecting over the anterodorsal corner of the orbit, a morphology otherwise unknown among lepidosaurs. The palpebral is present in the stem scincoid †*Parmeosaurus scutatus* (Gao and Norell, 2000) and in scincids and gerrhosaurine cordylids (Estes et al., 1988), but it is absent cordyline cordylids and in the cordylid sister, Xantusiidae (Gauthier et al., 2012). Among Lacertoidea, a palpebral is present in one basal branch (Lacertidae) but absent in the other (Gymnophthalmidae + Teiidae) (Estes et al., 1988). A palpebral is also absent in Amphisbaenia (Gauthier et al., 2012). A palpebral is, with few exceptions (e.g., *Heloderma*), present in anguimorphs (Maisano et al., 2002), but absent in all snakes (Gauthier et al., 2012). The palpebral is absent in all gekkotans, polyglyphanodonts, mosasaurs, and iguanians, as well in the stem lizard †*Huehuecuetzpalli mixteca*; Estes et al., 1988; Reynoso, 1998; Gauthier et al., 2012). From our examination, we currently regard the precise identification of this bone to be uncertain as we could not readily re-articulate it onto the prefrontal in our digital reconstruction of the skull (Fig. S1).

Prefrontal.**—**The left prefrontal (Fig. S1, Fig. S2P—R) of †*Eoscincus ornatus* is a triradiate bone that forms much of the anterodorsal orbital margin as in extinct (e.g., Gao and Norell, 2000; Čerňanský et al., 2022) and extant (e.g., Greer, 1970; Nash and Tanner, 1970; Nance, 2007; Gauthier et al., 2012; Paluh and Bauer, 2017; Camaiti et al., 2019) scincoids. The prefrontal contacts the maxilla anteriorly and the nasals at the anterior edge of the nasal process. The medial margin of the dorsal surface of the prefrontal forms a broad, wedge-shaped articulation for the frontal. The tapering orbital ramus of the prefrontal forms more than 70% of this articulation, which is otherwise composed by the base of the nasal ramus. The anteroposterior run of the orbital ramus of the prefrontal is nearly half that of the frontal itself. The anteriormost section of the maxillary ramus of the prefrontal is broken, as is the dorsal ramus of the maxilla (Fig. S1C). The ancestral condition, in which the maxilla does not overlap the prefrontal posterodorsally and is indeed excluded from the frontal by a broad nasal-prefrontal contact, is present in Rhynchocephalia and Iguania, whereas the maxilla overlaps the prefrontal to contact the frontal in gekkotans, scincoids, and lacertoids (Gauthier et al., 2012). Because of incomplete preservation of the maxillary ascending ramus, the condition in †*E. ornatus* cannot be determined. The lateral surface of the prefrontal is strongly convex and would have formed the medial border of the lacrimal foramen. Medially, the prefrontal bears an enlarged fossa that would have housed the olfactory chamber in life. The olfactory chamber fossa is enclosed posteriorly and dorsally by a sharp ridge of bone that separates the fossa from the frontal articular facet. In anterior view, the maxillary ramus is offset slightly medially. The orbitonasal margin of the prefrontal slopes ventrolaterally in *Sphenodon punctatus* and Iguania but is vertically to ventromedially sloped in crown scincoids, as well as the stem-scincoid †*Parmeosaurus scutatus,* and in lacertoids (e.g., Gauthier et al., 2012; Paluh and Bauer, 2017). The shape of this bone, and the orbitonasal fenestra it borders, are unclear in the Jurassic stem-gekkotans †*Ardeosaurus digitatellus* and †*Eichstaettisaurus schroederi* because they were flattened during preservation (see Simões et al., 2017). However, it is clear that the prefrontals are expanded in †*A. digitatellus* relative to †*E. ornatus* (Simões et al., 2017).

Frontal.**—** The frontals (Fig. S1, S4A—C) are represented by a fragment from the anterior end of the right element, and about the anterior three quarters of the left, are paired in †*Eoscincus ornatus*, differentiating this skull from the Jurassic squamate †*Ardeosaurus* (Simões et al., 2017). Among Jurassic lizards, the problematic taxon †*Parviraptor estesi* (Evans, 1994; Caldwell et al.,

2015), †*Dorsetisaurus purbeckensis* (Evans et al., 2006; Conrad, 2008), and DINO 15914 (Evans and Chure, 1998; this paper) possess paired frontals. The frontals of †*Eichstaettisaurus* spp. have variously been interpreted as fused (e.g., Simões et al., 2017) and unfused (Gauthier et al., 2012). Simões et al. (2017) also interpreted the frontals of †*Ardeosaurus digitatellus* as fused. The frontals are also fused in the Triassic pan-squamate †*Megachirella wachtleri* (Simões et al., 2018) and the Cretaceous pan-squamate †*Huehuecuetzpalli mixteca* (Reynoso, 1998). Frontal fusion is highly variable among squamates (Gauthier et al., 2012), and both states, paired and fused, are found in extant and extinct scincoids and lacertoids (e.g., Nash and Tanner, 1970; Hutchinson et al., 1989; Caputo, 2004; Folie et al., 2005; Nance, 2007; Čerňanský and Smith, 2018; Čerňanský et al., 2017; Paluh and Bauer, 2017; Camaiti et al., 2019; Čerňanský and Syromyatnikova, 2019; Cruzado-Caballero et al., 2019; Bates and Stanley, 2020). The better-preserved left frontal is subrectangular in shape and has roughly the same mediolateral width along its anteroposterior axis as preserved. The region around the fronto-parietal suture is not preserved, so the degree of posterior expansion to that suture cannot be determined. Nevertheless, there is no indication that the frontals were constricted between the orbits as in iguanians, carusiids, and xenosaurids (Gauthier et al., 2012). Their dorsal surfaces show subtle rugosities, as in †*Parmeosaurus scutatus* (Gao and Norell, 2000), IGM 3/54 (Gao and Norell, 2000), †*Slavoia darevskii* (Tałanda, 2016), and †*Scincoideus grassator* (Folie et al., 2005). Heavier osteodermal crusts overly the frontals in †*Myrmecodaptria microphagosa* and †*Carusia intermedia* (Gao and Norell, 1998, 2000), lacertids (e.g., Muller et al., 2011; Čerňanský and Smith, 2018; Čerňanský et al., 2017; Čerňanský and Syromyatnikova, 2019; Cruzado-Caballero et al., 2019), cordylids (Nance, 2007; Bates and Stanley, 2020), and scincids (Nash and Tanner, 1970; Hutchinson et al., 1989; Caputo, 2004; Gelnaw, 2011; Gauthier et al., 2012; Paluh and Bauer, 2017; Camaiti et al., 2019). We cannot determine the size and shape of the fronto-parietal scales, but there appears to have been a large frontal scute overlying the frontals in life.

The frontals are ventrally concave and the subolfactory processes are broken away, as revealed by hollows or cancellous bone sandwiched between compact bone marking the margins of the subolfactory processes ventrally (Fig. S4B). As a consequence, it cannot be determined if †*Eoscincus borealis* can be distinguished from the frontals of lacertoids in this regard (including Amphisbaenia; Kearney, 2003; Kearney et al., 2005; Müller et al., 2011; Bolet et al., 2014; Čerňanský et al., 2017; Čerňanský and Smith, 2018; Čerňanský and Syromyatnikova, 2019;

Cruzado-Caballero et al., 2019), or from those of scincoids (Gauthier et al., 2012; Camaiti et al., 2019), gekkotans (Estes et al., 1988; Gauthier et al., 2012), anguimorphs, mosasaurs, and snakes (Estes et al., 1988; Gauthier et al., 2012). In these taxa, the frontal subolfactory processes are enlarged, and in some cases contact each other on the ventral midline (Gauthier et al., 2012). Poorly developed subolfactory processes are retained in Rhynchocephalia and Iguania (Estes et al., 1988), and in the stem squamates †*Megachirella wachtleri* and †*Huehuecuetzpalli mixteca* Gauthier et al., 2012).

Epipterygoid.—A long, slender fragmentary element (Fig. S4D—E) originally identified as the hyoid by Evans and Chure (1998) is considered here to be the ventral third of the left epipterygoid based on its position *in situ* just posterior to the bones bordering the orbit and medial to the mandible (Fig. 1A—B) and by its straight shaft. Because only a fragment of this bone is preserved, all that can be said is that the epipterygoid of †*Eoscincus ornatus* was long, rod-like, and hollow.

Vomer.—Both vomers of †*Eoscincus ornatus* were preserved in semi-articulation with the rest of the skull (Fig. 1D, Fig. S3A—L; Fig. S5A). The left vomer is excellently preserved and provides the first look at the anterior palate of an early squamate. This is important because of the sensory significance of the vomer in squamates (e.g., Schwenk, 1993, 1995; Rieppel et al., 2008; Brykczynska et al., 2013). The vomers in †*E. ornatus* are unfused. This is the plesiomorphic squamate condition (Rieppel et al., 2008; Gauthier et al., 2012) and differentiates the palate of this lizard from the fully fused vomers of xantusiids (Gauthier et al., 2012) and lygosomine scincids, including *Eremiascincus richardsonii* (Gelnaw, 2011), *Trachylepis* spp. (Gauthier et al., 2012; Paluh and Bauer, 2017), *Sphenomorphus solomonis*, *Eugongylus rufescens*, and *Tiliqua scincoides* (Gauthier et al., 2012). The crown scincids *Acontias percevali, Brachymeles gracilis*, and *Eumeces fasciatus* possess unfused vomers, whereas those in *Amphiglossus splendidus* and *Feylinia polylepis* are fused anteriorly (Gauthier et al., 2012). Cordylids possess paired vomers, as does the Cretaceous pan-scincoid †*Parmeosaurus scutatus* (Gauthier et al., 2012) and the tentative pan-xantusiid †*Retinosaurus hkamtiensis* (Čerňanský et al., 2022). The condition in †*Tepexisaurus tepexii* (Reynoso and Callison, 2000) is unknown. The anterior end of the vomer is bifurcated into two small processes: the inferior and superior premaxillary processes. The inferior premaxillary processes forms the medial boundary of the external vomeronasal fenestra, which is reduced in †*E. ornatus* relative to crown scincids (Fig.

S5B; e.g., Nash and Tanner, 1970; Hutchinson et al., 1989; Caputo, 2004; Paluh and Bauer, 2017; Camaiti et al., 2019), globaurids (*sensu* Gauthier et al., 2012), cordylids (e.g., Nance, 2007), †*Carusia intermedia* (Gao and Norell, 1998, 2000), lacertoids (Fig. S5D; Čerňanský et al., 2017; Čerňanský and Smith, 2018; Čerňanský and Syromyatnikova, 2019), and anguimorphs and snakes (Gauthier et al., 2012). Surprisingly, external vomeronasal fenestra in †*E. ornatus* is also less developed than those in iguanians and gekkotans (Fig. S5F—G) and most closely matches the extremely reduced condition in xantusiids (Fig. S5C; Gauthier et al., 2012). The inferior and superior premaxillary processes run approximately parallel to each other and are oriented anteromedially. These processes are similar in size and connected by a thin wing of bone. A small process appearing in the region bordered anteriorly by the premaxillary processes and posteriorly by the vomerine fossa is identified as Jacobson's process. This process supports the ventral margin of the cartilaginous concha of Jacobson's organ in extant lizards (e.g., Klembara et al., 2017). The dorsal opening of the vomerine foramen is placed at the anterior border of the vomerine fossa just posterior to Jacobson's process and adjacent to the septomaxillary process. In anguimorphs, the dorsal vomerine foramen is placed within the vomerine fossa (e.g., Klembara et al., 2017; Ledesma and Scarpetta, 2018). Posterior to the dorsal vomerine foramen, the vomerine fossa appears as a deep excavation comparable to the condition in extant scincids (e.g., Paluh and Bauer, 2017; Camaiti et al., 2019) and cordylids (e.g., Nance, 2007) but not xantusiids (Gauthier et al., 2012). The posterior margin of the vomer is concave and terminates in a short palatine process. The left vomer and palatine are still in partial articulation *in situ* and show that the vomerine process of the palatine is overlapped dorsally by the posterior end of the vomer. The posterior end of the dorsal surface of the vomer bears a subtle, v-shaped depression to contact the vomerine process of the palatine in a tongue-in-groove articulation. The medial wall of the vomerine fossa is deeper than the lateral wall and is undercut by a subtle ventral crest.

There are at least three vomerine foramina on the ventral surface. One opens anteriorly below the premaxillary process, whereas the others are placed along the ventral surface of the vomer just posterior to the external vomeronasal fenestra. These foramina are separated by raised, anteroposteriorly oriented ridges that resemble the structures found in anguimorphs (Gauthier et al., 2012; such as *Pseudopus apodus* (Fig. S5D; Klembara et al., 2017), *Elgaria panamintina* (Ledesma and Scarpetta, 2018), and pan-varanoids (e.g., Rieppel, 1980; Rieppel

and Grande, 2007; Norell et al., 2008). The vomerine foramina would have housed the medial branch of the palatine ramus of cranial nerve VII (Klembara et al., 2017).

Posteriorly, the ventral margin of the vomer forms a raised region that bears at least two identifiable rows of vomerine teeth. This raised platform may be homologous with the small, paired bumps found at the posterior terminus of the ventral surface of the vomer in some extant scincids such as *Eumeces* (*=Plestiodon*) *fasciatus* (Fig. S5B) and *Trachylepis* (Paluh and Bauer, 2017) but absent in others (e.g., *Eremiascincus richardsonii*; Gelnaw, 2011). The vomerine teeth are the largest in the palatal tooth row and closely resemble the premaxillary, maxillary, and dentary teeth in morphology. The presence of vomerine teeth in †*Eoscincus ornatus* represents the first published occurrence of multiple vomerine tooth rows in a squamate (Matsumoto and Evans, 2017). Vomerine teeth are ancestral for lepidosaurs and are lost within both rhynchocephalians and squamates (Evans, 1980; Mahler and Kearney, 2006; Gauthier et al., 2012; Matsumoto and Evans, 2017). Small, recurved teeth are known for the serpentiform anguimorph *Pseudopus apodus*, but their presence and size are intraspecifically variable (Klembara et al., 2017; Matsumoto and Evans, 2017; C.D.B. and J.A.G. pers. obs.) and they are absent in other extant anguine anguids. Vomerine teeth are also reported for a Middle Jurassic Chinese squamate (see Dong et al., 2019), although this unnamed skeleton has yet to be described. Single rows of vomerine teeth are present in some stem-varanoids, including †*Eosaniwa koehni* (Rieppel et al., 2007) and †*Paranecrosaurus fiesti* (Smith and Habersetzer, 2021). Glyptosaurine stem anguids (according to Gauthier et al., 2012) have toothless vomers (e.g., †*Peltosaurus granulosus*) like other anguids (apart from some *P. apodus*). Moreover, the Early Eocene glyptosaurine †*Melanosaurus maximus* has a long, narrow, dense patch of teeth on the vomer (Gilmore, 1928: plate 24; we consider patches to represent vomerine teeth which are not distributed in discrete antero-posterior lineations). Finally, a few specimens of *Sphenodon punctatus* apparently still possess one or two small teeth on the vomer (Evans, 2006). The presence of vomerine tooth rows in the stem-scincoid †*E. ornatus* suggests that vomerine teeth had a more complex history within squamates than is currently appreciated, with several independent evolutionary losses and/or reversals, regardless of whether one prefers molecular (e.g., Burbrink et al., 2020) or morphological (e.g., Gauthier et al., 2012) trees. The vomerine tooth rows in †*Eoscincus ornatus* enclose a sub-ovoid region at the posterior end of the ventral surface of the vomer that is comparable to the condition in the Early Jurassic rhynchocephalian

†*Gephyrosaurus bridensis* (Evans, 1980; Conrad, 2008). However, the absence of vomerine teeth along the anterior portion of the ventral surface of the vomer indicates that the configuration of vomerine teeth in †*E. ornatus* is unique among amniotes with multiple rows (see Matsumoto and Evans, 2017, for a review of amniote palatal teeth).

Palatine.—Only the left palatine (Fig. S1, Fig. S3M—Q) was preserved in †*Eoscincus ornatus*. This bone is triradiate and shows that †*E. ornatus* lacked a bony secondary palate. Extant lygosomine scincids are characterized by the presence of a bony secondary palate in which the palatine borders the choanal duct dorsally, laterally, and ventrally (Greer, 1970; Rieppel et al., 2008; Gauthier et al., 2012). Basal scincids and globaurid stem-scincids only show varying degrees of palatine underlap of the choana, more so in the former and less so in the latter and are only considered to have an incipient degree of development of a secondary palate (Gauthier et al., 2012). Gerrhosaurines show the expansion of the palatine to dorsally border part of the choanal duct (e.g., Nance, 2007). Some gymnophthalmids have a fully developed secondary palates, but it is formed by the maxilla and vomer (Presch, 1980; see also *Cricosaura typica*; Savage, 1963), and they possess only modestly developed palatine subchoanal processes (Gauthier et al., 2012). Diploglossine (Hernández Morales et al., 2019) and gerrhonotine (Ledesma and Scarpetta, 2018) anguids show an incipient development of the palatine subchoanal process (see also *Anniella pulchra*; Gauthier et al., 2012). The secondary palate is formed by extensions of ectochoanal cartilage rather than the palatines in xantusiids (Savage, 1963; Rieppel et al., 2008), in which distinct processes of the palatine underlapping the choanal duct are absent. †*Carusia intermedia* (Gao and Norell, 1998), lacertids (e.g., Čerňanský et al., 2017; Čerňanský and Syromyatnikova, 2019), teiids (Gauthier et al., 2012), and other non-scincid squamates (Estes et al., 1988) lack any indication of secondary palates.

Anteriorly, the broad vomerine process of the palatine in †*Eoscincus ornatus* projects from the medial side of the palatine and is clasped by the palatine process of the vomer (Fig. 1D). The lateral margin of the anterior palatine is developed into articular processes and surfaces contacting the maxilla and jugal. The palatine articulates with the maxilla anterior to the posterior termination of the vomer in the palate (Fig. 1D; Gauthier et al., 2012). The jugal articulation is located on the posterior ramus, which is posteriorly offset from the maxillary articulation and forms the lateral border of a small infraorbital foramen. This foramen is contained entirely within the palatine, as in other scincids, †*Parmeosaurus scutatus*, lacertids,

anguimorphs, and snakes but unlike other squamates and *Sphenodon punctatus* (Gauthier et al., 2012). The anterior end of the palatine bears a small crest for the orbitonasal membrane in anterior view and articulated with the prefrontal anterodorsally.

The deepened choanal fossa is placed between the vomerine process and maxillary articulation on the ventral surface of the palatine. As in scincoids (e.g., Nash and Tanner, 1970; Nance, 2007; Rieppel et al., 2008; Gauthier et al., 2012; Paluh and Bauer, 2017; Camaiti et al., 2019), lacertoids (e.g., Čerňanský et al., 2017; Čerňanský and Syromyatnikova, 2019), and anguids such as *Pseudopus apodus* (Klembara et al., 2017) and *Elgaria panamintina* (Ledesma and Scarpetta, 2018), the choanal fossa runs for at least half the length of the palatine (Gauthier et al., 2012). The choanal fossa is separated from the pterygoid articular surface on the medial side of the palatine by a small region of bone. Laterally, the choanal fossa is bordered by a raised region on the ventral surface of the palatine that represent the border of the suborbital fenestra. CT cross-sections of the skull show that the palatine bears a row of very small teeth on the medial side of the posterior end of its ventral surface. Palatine teeth occur in many anguimorphs, including Cretaceous stem taxa (Gauthier et al., 2012), most snakes (except scolecophidians, uropeltids, and *Calabaria reinhardtii*; Gauthier et al., 2012), and the stem scincoid †*Parmeosaurus scutatus* (Gao & Norell, 2000). Like vomerine teeth, palatine teeth are ancestral for Amniota, Reptilia, Sauria, and Lepidosauria (e.g., Mahler and Kearney, 2007; Gauthier et al., 1988; Matsumoto and Evans, 2017). But they have been lost in all extant amniotes (Matsumoto and Evans, 2017) apart from *Sphenodon punctatus* and its extant sister Squamata (e.g., Gauthier et al., 2012). The placement of †*Eoscincus ornatus* at the base of Pan-Scincoidea supports the hypothesis that palatal teeth were independently lost multiple times in squamates. Based on morphological (Gauthier et al., 2012), molecular (e.g., Pyron et al., 2013; Zheng and Weins, 2016; Burbrink et al., 2020), and combined (Reeder et al., 2015; Simões et al., 2018) analyses, palatine teeth were independently lost in polyglyphanodonts, mosasaurs, all crown and some stem gekkotans (e.g., †*Norellius nyctisaurops* Conrad and Daza, 2015), dibamids, many iguanian clades (e.g., pan-acrodonts), lacertoids (including amphisbaenians), crown-scincoids (Gauthier et al., 2012), in several anguimorph clades (e.g., Xenosauridae, *Heloderma*, *Varanus*), and at least three times in snakes. The loss of palatine teeth must have occurred early in scincoid evolution, as they are present in the Late Cretaceous stem-scincoid †*Parmeosaurus scutatus* (Gao and Norell, 2000) but are absent in the Early Cretaceous stem-xantusiid (Gauthier et al., 2012)

†*Tepexisaurus tepexii* (Reynoso and Callison, 2000). All crown scincids lack palatine teeth, as do the successively more remote stem scincids of the Late Cretaceous (Gauthier et al., 2012), the globaurids (*sensu* Gauthier et al., 2012) and carusiids (e.g., †*Carusia intermedia*; Gao and Norell, 1998, 2000).

Ectopterygoid.—The left ectopterygoid of †*Eoscincus ornatus* is preserved in articulation with the left pterygoid (Fig. 1D, Fig. S1; Fig. S3S—W) and formed much of the lateral and posterior borders of the suborbital fenestra. The ectopterygoid does not bifurcate anteriorly to form two maxillary processes as in anguimorphs (Klembara et al., 2017; Ledesma and Scarpetta, 2018). Instead, it retains the single maxillary process found in pan-scincoids (Nash and Tanner, 1970; Hutchinson et al., 1989; Gao and Norell, 1998, 2000; Reynoso and Callison, 2000; Caputo, 2004; Nance, 2007; Paluh and Bauer, 2017; Camaiti et al., 2019). In many lacertoids, the maxillary process of the ectopterygoid shows some degree of bifurcation (e.g., *Ptychoglossus vallensis*; Hernández Morales et al., 2019) distinguishing this group from †*E. ornatus*, scincids (e.g., Hutchinson et al., 1989; Gelnaw, 2011; Paluh and Bauer, 2017; Camaiti et al., 2019), globaurids, xantusiids, cordylids, and †*Carusia intermedia* (Gauthier et al., 2012). Based on the structure of this element in †*E. ornatus* in anterior view (Fig. S3U), the ectopterygoid appears to have possessed the apomorphic longitudinal groove that clasped the maxilla as in all lacertoids (including amphisbaenians), scincoids save for pan-scincids, all anguimorphs, and the stem snake †*Dinilysia patagonica* (Gauthier et al., 2012). The ectopterygoid smoothly curves medially halfway along its anteroposterior run as in other squamates aside from iguanians, which retain the sharper angle between these rami seen in rhynchocephalians (Gauthier, 2012). Posteriorly, the ectopterygoid bifurcates into the dorsal and ventral pterygoid processes. The medial half of the anterior portion of the pterygoid is clasped by the long pterygoid processes of the ectopterygoid. The dorsal pterygoid process of the ectopterygoid is approximately twice as long as the ventral process. However, the ventral pterygoid process is dorsoventrally twice as deep as the dorsal pterygoid process.

Pterygoid.—The anterior third of the left pterygoid was preserved in articulation with the left ectopterygoid (Fig. 1D, Fig. S1, Fig. S3X—Y). The anterior portion of the pterygoid bifurcates into two distinct processes: the palatine process and the ectopterygoid process. The palatine process is broader and flatter than the ectopterygoid process. In cordylids, the palatine process can widen to three times the mediolateral width of the ectopterygoid process (see fig. 8 in Nance,

2007; Gates and Stanley, 2020); the palatine process of the pterygoid †*Eoscincus ornatus* is more comparable to the slender processes found in many scincids (Nash and Tanner, 1970; Hutchinson et al., 1989; Paluh and Bauer, 2017) and xantusiids (Gauthier et al., 2012: Appx. 2, fig. 271). It is impossible to determine the anterior extent of the palatine process relative to the ectopterygoid process, as the anteriormost part of the former is not preserved. However, the ectopterygoid process is much more developed in †*E. ornatus* than in †*Parmeosaurus scutatus,* wherein the ectopterygoid process is less than one fourth the length of the palatine process (Dong et al., 2018). Dorsally, the posterior margin of the ectopterygoid process is bordered by a ridge identifiable as the transverse process.

The palatine and ectopterygoid processes are separated by a deep, U-shaped suborbital incisure (=the posterior boundary of the suborbital fenestra). The development of the suborbital incisure is variable in anguimorphs (e.g., Gao and Norell, 2000; Klembara et al., 2017; Ledesma and Scarpetta, 2018). In lacertoids (e.g., Čerňanský et al., 2017; Čerňanský and Syromyatnikova, 2019; Hernández Morales et al., 2019), the suborbital incisure is widened relative to the condition in †*Eoscincus ornatus*, scincids (Nash and Tanner, 1970; Hutchinson et al., 1989; Rieppel et al., 2008; Paluh and Bauer, 2017), and gerrhosaurines (Nance, 2007). The suborbital incisure is poorly developed in †*Carusia intermedia* (Gao and Norell, 2000) and widened in †*Parmeosaurus scutatus* (Dong et al., 2018).

The ventral surface of the palatine process of the pterygoid bears numerous protrusions that are identifiable as small pterygoid teeth. Pterygoid teeth are present in †*Parmeosaurus scutatus* (Dong et al., 2018), †*Carusia intermedia* (Gao and Norell, 1998), and in several species of extant scincoids (e.g., Mahler and Kearney, 2007; Matsumoto and Evans, 2017). According to Mahler and Kearney (2007; see also Matsumoto and Evans, 2017), pterygoid teeth are also present in many non-acrodontan iguanians, in all mosasaurs, and in most snakes (except scolecophidians, uropeltids, and *Calabaria reinhardtii*), as well as in many anguimorphs (except *Xenosaurus*, *Anniella,* and *Varanus*). Pterygoid teeth were lost in gekkotans, acrodont iguanians, and several times in lacertoids (including in amphisbaenians as well as the amphisbaenian-like Cretaceous lizard †*Slavoia darevskii*; Tałanda, 2016), in all pan-xantusiids (Gauthier et al., 2012), and in †*Retinosaurus hkamtiensis* (Čerňanský et al., 2022). While still present in one Cretaceous carusiid (*sensu* Gauthier et al., 2012) (i.e., †*Carusia intermedia*; Gao and Norell, 1998), they are notably reduced. †*Myrmecodaptria microphagosa* lacks palatal teeth entirely

(Gao and Norell, 2000). Likewise, although pterygoid teeth are present in some globaurid stem-scincids from the Late Cretaceous (e.g., †*Globaura venusta*, †*Hymenosaurus clarki*), they are absent in others (e.g., †*Eoxanta lacertifrons*) (Gauthier et al., 2012).

First Ceratobranchial.—A fragment of bone we tentatively identify as the first ceratobranchial (Fig. S3Y—Z) was preserved near the displaced left pterygoid and ectopterygoid. This bone is long, slender, and slightly dorsoventrally bowed posteriorly.

Dentary and Splenial.— Besides their posterior portions, both dentaries were well preserved in †*Eoscincus ornatus* (Fig. 1, Fig. S1, Fig. S4F—H). The dentaries are robust, and each possess 23 tooth positions. The dentary bears five mental foramina on the lateral surface that increase in size posteriorly. Mental foramina on the dentary are more numerous in the Paleogene scincoid †*Scincoideus haininensis* (8 in the holotype dentary; Folie et al., 2005: fig. 1) and in the putative fossil scincoids from the Maastrichtian of western North America (Longrich et al., 2012). However, †*E. ornatus* and †*Parmeosaurus scutatus,* carusiids, scincids, and lacertoids share similar counts (e.g., Nash and Tanner, 1970; Gao and Norell, 1998, 2000; Muller et al., 2011; Čerňanský and Smith, 2018; Čerňanský et al., 2017; Paluh and Bauer, 2017; Camaiti et al., 2019; Čerňanský and Syromyatnikova, 2019; Hernández Morales et al., 2019; Thorn et al., 2021). The dentary symphyses are medially curved such that they contact each other on the midline. The dorsal half of the anterior end of each dentary extends farther anteriorly than the ventral margin, and the dentaries deepen posteriorly. The Meckelian canal is open, contrasting with at least some degree of restriction of this canal in several iguanians (Gauthier et al., 2012). The splenial is large, tapers anteriorly, and is punctured by the anterior inferior alveolar and anterior mylohyoid foramina. This bone runs for approximately three-fourths of the length of the dentary tooth row and is in continuous contact with the dentary. This contrasts with the condition in other pan-scincoids wherein the splenial loses contact with the dentary anterior to the anterior inferior alveolar foramen. The elongated anterior inferior alveolar foramen is placed slightly anterodorsal to the smaller and more oval anterior mylohyoid foramen. In †*Parmeosaurus scutatus*, these foramina are widely separated (Gao and Norell, 2000).

The posterior margin of the dentary in †*Eoscincus ornatus* is poorly preserved on both sides. Nevertheless, the dentary preserves the classic scincoid synapomorphy of a prominent coronoid process that projects dorsally onto the anterolateral surface of the coronoid bone (e.g., Gauthier et al., 2012). The dentary teeth are pleurodont, vertically implanted, and unicuspid

(Figure 2B, contrasting with the tricuspid pleurodont teeth present in †*Parmeosaurus scutatus* (Gao and Norell, 2000), †*Scincoideus haininensis* (Folie et al., 2005), and some scincids, cordylids, and xantusiids (e.g., Kosma, 2004; Gauthier et al., 2012). As noted, many scincids lack the condition shared among paramacellodids and some cordylids wherein two dominant striae run up the lingual surface of the crown and meet at the lingual cusp apex (Richter, 1994; Kosma, 2004; though see Thorn et al., 2021). Nutrient foramina are set on the midline at the tooth bases, consistent with vertical replacement, rather than posteriorly set as in the interdental replacement of anguimorphs, mosasaurs, and snakes (McDowell and Bogert, 1954). The six anteriormost teeth are slightly procumbent.

Coronoid.—Only the left coronoid was preserved in †*Eoscincus ornatus* (Fig. S1, S4I—L). As in most squamates except for mosasaurs, dibamids, amphisbaenians, and snakes (aside from the stem snake †*Dinilysia patagonica*; Gauthier et al., 2012), the coronoid is a large, triradiate bone that sits atop the surangular and forms the entirety of the coronoid eminence. Anteriorly, the dentary anterolaterally overlaps the coronoid. The posteroventral portion of the medial surface of the coronoid articulates via a facet on the surangular and prearticular portions of the fused compound bone (see below). The portion of the coronoid that articulated with the splenial is not preserved, although a fragment of the dentary process of the coronoid may be preserved in semi-articulation with the left dentary (Fig. S4H).

Angular.—Although no identifiable portions of the angular were preserved, the bone appeared to be free as there are traces of its sutures on the surangular (Fig. S4G, I). The suture outline indicates that the angular was slightly visible in lateral view and formed a portion of the ventral margin of the mandible between the posteroventral termination of the dentary and the anterior end of the prearticular part of the compound bone.

Fused postdentary elements.—In †*Eoscincus ornatus*, the prearticular, articular, and surangular are fused together to jointly comprise the posterior third of the mandible (Fig. S4I—L). This ossified set of elements is referred to as the compound bone by some authors (e.g., Paluh and Bauer, 2017). The complete fusion of these bones is present throughout Scincoidea (Gauthier et al., 2012). In fact, fusion is so common among full-grown squamates (*sensu* Petermann and Gauthier, 2020), with younger individuals having separate elements, that the presence of a compound bone in †*E. ornatus* indicates that the type specimen represents a fully adult individual. That being said, coossification apparently occurs much earlier in ontogeny among

snakes, dibamids, and amphisbaenians (e.g., Kearney, 2003; Kearney et al., 2005; Bolet et al., 2014), as a discrete surangular has never been reported in these taxa (e.g., Greer, 1985; Lee, 1998).Laterally, the surangular forms the majority of the compound bone. A subtly developed surangular crest is present approximately halfway along the dorsoventral axis of the lateral surface of the compound bone. Medially, the surangular bears a deep adductor fossa. A small circular hole in the bone located at the posterior corner of the adductor fossa may be the posterior surangular foramen, although this identification is tentative because the bone surface of the prearticular just posterior to the adductor fossa is broken. Posterior to the surangular, the fused articular and prearticular form the base and body of the retroarticular process. The retroarticular process, which is formed by the prearticular in squamates (Gauthier et al., 1988), is poorly preserved. Beyond its presence, we were unable to determine details of its anatomy.

Microteras borealis.

Maxilla.—The right maxilla (Fig. S6A—D) of †*Microteras borealis* is very similar to that of †*Eoscincus ornatus* and other stem scincoids, including †*Parmeosaurus scutatus* (Gao and Norell, 2000). The bone comprised the majority of the anterior external skull and bore an anteroposteriorly broad ascending (facial) ramus of which only the base remains. The premaxillary process is proportionally shorter than in †*E. ornatus* and forms a gentle arc with the anterior margin of the ascending ramus of the maxilla to border the external naris ventrally and posteriorly. Unlike †*E. ornatus,* but similar to other Mesozoic pan-scincoids such as †*Parmeosaurus scutatus* and the potential early pan-scincid carusiids (*sensu* Gauthier et al., 2012 = †*Carusia intermedia* and †*Myrmecodaptria microphagosa* of Gao and Norell, 1998, 2000), the mental foramina on the lateral surface of the maxilla are small. Five mental foramina in total are observable. The jugal ramus is long and slender. The articular surface for the jugal on the maxilla is widely separated from the articular surface for the prefrontal as in scincoids (e.g., Gauthier et al., 2012). Medially, the maxilla bears a straightened palatine shelf and a large superior alveolar foramen. The premaxillary process is prominently bifid and forms a squared-off outline at its base, as seen, for example, in scincoids such as *Eumeces* (=*Plestiodon*) *laticeps* and *Cordylosaurus subtessellatus.* Based on other pan-scincoids, the lateral flange presumably overlapped the premaxilla, and the medial flange contacted the septomaxilla. These flanges are broadly parallel in orientation, contrasting with the strongly medially offset septomaxillary flange of †*Eoscincus ornatus*. The articulations for the septomaxilla, vomer, and palatine are not

apparent. The maxilla bears 15 teeth as preserved, though the empty alveoli confirm that 20+ teeth were present. This contrasts with the 19 tooth positions found in the maxillae of †*Eoscincus ornatus*. These teeth are homodont and tricuspid contrasting with the slightly recurved, unicuspid dentition of †*E. ornatus*. The maxillary crowns in *M. borealis* bear prominent apices and are deflected lingually. CT scan-slices are insufficiently fine to determine if the tooth crowns are striated, but prominent striations do not appear upon examination of the specimen under a light microscope.

***Braincase.*—**The braincase of †*Microteras borealis* (Fig. S6E—H) is nearly complete, lacking only the basipterygoid processes and the anterior portion of the orbitotemporal region, and part of the supraoccipital. The internal surface is also excellently preserved, facilitating the creation of an endocast (Fig. S8). Sutures are not visible, indicating that the sphenoid, basioccipital, supraoccipital, prootic, and otooccipital are fused as in skeletally mature adults of extant squamates (e.g., Gauthier et al., 2012; Paluh and Bauer, 2017; Petermann and Gauthier, 2020).

The sphenoid is presumably formed from the fused parasphenoid and basisphenoid and would be fused to the prootic dorsally and basioccipital posteriorly as in extant squamates (e.g., Gauthier et al., 2012). Although the ventral surface of the braincase is incomplete, we infer †*Microteras borealis* possessed a cultriform process based on the presence of a raised region of bone in the center of the ventral surface. In †*Parmeosaurus scutatus*, a similar feature is continuous with the cultriform process anteriorly (Gao and Norell, 2000; Dong et al., 2018). The posterior section of the jugular vein recess is visible as a small, deep sulcus oriented parallel to the anteroposterior axis of the braincase.

Although the extensive fusion of the braincase in †*Microteras borealis* makes an assessment of the composition of the occipital condyle difficult, we presume the basioccipital formed most of its ventral portion. The basioccipital would presumedly be fused anteriorly with the sphenoid and laterally with the otooccipitals. The basal tubera are probably placed at the posterolateral corners of the basioccipital, as in †*Parmeosaurus scutatus* (Dong et al., 2018), †*Retinosaurus hkamtiensis* (Čerňanský et al., 2022), and extant scincids (Nash and Tanner, 1970; Gelnaw, 2011; Paluh and Bauer, 2017), cordylids (Nance, 2007; Bates and Stanley, 2020), and xantusiids (Gauthier et al., 2012: Appx. 2, Fig. 32). The basal tubera are well developed in †*E. ornatus*. However, there appears to be an allometric relation among squamates in this site of subcervical muscle attachment that has yet to be fully explored. For example, smaller gekkotans

(e.g., *Coleonyx variegatus*; Fig. S8) have reduced basal tubera, but they are prominent in larger species (*Gecko gecko*) (Daza et al., 2013). The same is true of scincids (smaller *Acontias percivali* vs. larger *Tiliqua scincoides*), xantusiids (smaller *Xantusia vigilis* vs. larger *X. riversiana*), and cordylids (smaller *Cordylosaurus subtessallatus* vs. larger *Smaug mossambicus*). Reduction of basal tubera has figured in debates about the relationships of major clades of serpentiform squamates: dibamids, amphisbaenians, and snakes. Basal tubera are reduced in dibamids (e.g., Greer, 1985), but then they have tiny heads. Most amphisbaenians also have smaller heads and reduced basal tubera (e.g., Kearney, 2003; Kearney et al., 2005; Bolet et al., 2014), though they are poorly developed even in some large-headed species (e.g., *Amphisbeania fuliginosa* and *A. alba*). However, that is not the case for the rhineurid sister to all other crown amphisbaenians, for although they are reduced in the smaller *Rhineura floridana* they are still prominent in the larger †*Spathorhynchus fossorium.* Only snakes appear to have reduced basal tubera regardless of head size (Rieppel and Zaher, 2000).

The dorsal surface of the preserved portion of the braincase in †*Microteras borealis* is formed by the supraoccipital, although no clear sutures remain between this bone and other fused parts of the braincase. This element is partially preserved in the main section of this element in the †*M. borealis* holotype YPM 4718, but additional fragments may represent the missing portion (Fig. S7I). It fuses with the prootics anterolaterally and the otooccipitals ventrolaterally. Posteriorly, the supraoccipital would form the dorsal margin of the foramen magnum, although that region is not preserved.

The prootic is completely preserved where it joins the rest of the braincase. The outline of the prootic is difficult to discern due to the absence of sutures throughout the braincase, but we infer that this bone fused with adjacent braincase elements in a manner similar to *Parmeosaurus scutatus* (Dong et al., 2018) and extant scincoids (Nance, 2007; Gelnaw, 2011; Paluh and Bauer, 2017), as in squamates ancestrally (Gauthier et al., 1988). The prootic houses a large portion of the osseous labyrinth, including the vestibule and anterior and horizontal semicircular canals. The alar process of the prootic is largely broken away, but enough of its base anterodorsal to the anterior semicircular canal is preserved to determine that it was similar in development to those of extant scincoids (e.g., Nance, 2007; Gelnaw, 2011; Paluh and Bauer, 2017) and †*Parmeosaurus scutatus* (Dong et al., 2018). The development of the alar process in squamates is an important character in debates over the placement of Iguania, which lacks this feature as in

Sphenodon punctatus and other saurians (Gauthier, 1982; Gauthier et al., 1988). A prominent alar process of the prootic is present in all other squamates aside from scolecophidian snakes, and it is much reduced in dibamids (e.g., *Anelytropsis papillosus*; state 2 in Fig. 312 in Gauthier et al., 2012) and inalethinophidian snakes (Gauthier et al., 2012; Reeder et al., 2015). Gekkotans possess a prominent alar process, though it is pointed, rather than rounded, at its anterior apex (e.g., Gauthier, 1982; Gauthier et al., 2012; Villa et al., 2018). The anterior semicircular canal and its ampulla are placed just posterior to the alar process. The former feature curves posteroventrally. The anterior semicircular canal is undercut by a widened prootic incisure, which itself delimits the intersection of the developed prootic crest and the main body of the braincase. The horizontal semicircular canal and its associated ampulla are located posterior to the anterior semicircular canal and ventrally bounded by the prootic crest. The medial surface of the prootic bears at two exit foramina for cranial nerve VII along the anterior margin of the auditory recess. The exit foramen for cranial nerve VIII is located at the posterior end of this feature. The endolymphatic foramen could not be identified.

The otooccipitals—formed by fusion of the exoccipital and opisthotic in late embryos in squamates (e.g., Estes et al., 1988)—are located posterior to the prootics and form most of the lateral margins of the sub-ovoid foramen magnum. The anterior section of the otooccipital houses the posterior portion of the horizontal semicircular canal, which is bordered posteriorly by the paroccipital process and by the oval fenestra posteroventrally. The interfenestral crest in †*Microteras borealis* is widened relative to the condition in extant scincids (Gelnaw, 2011; Paluh and Bauer, 2017), lacertoids (Čerňanský et al., 2017; Hernández Morales et al., 2019), and †*Parmeosaurus scutatus* (Dong et al., 2018). Instead, the development of the interfenestral strut compares favorably with cordylids (Gauthier et al., 2012; Bates and Stanley, 2020) and anguimorphs (Bever et al., 2005; Klembara et al., 2017; Ledesma and Scarpetta, 2018). The lateral aperture of the recessus scalae tympani (LARST) appears to be placed in the anteroventral corner of the occipital recess, which is bounded posteriorly by the tuberal crest. A single foramen for the vagus nerve (cranial nerve X) lies dorsolateral to the three foramina for the hypoglossals (cranial nerve XII) exiting the braincase from the posterior surface of the otooccipital. Medially, the vagus foramen (= jugular foramen of other reptiles) appears as a small vertical slit, the remnant of the upper part of the embryonic metotic fissure.

Hindbrain and Labyrinth.— Both the left and right inner osseous labyrinths and most of the medulla are preserved and remain uncrushed, enabling a detailed examination of the inner ear and brain anatomy of †*Microteras borealis* (Fig. S7). The medulla is subequal in anteroposterior length and mediolateral width, contrasting with the elongated hindbrains of snakes (Allemand et al., 2017; Triviño et al., 2018; Macrì et al., 2019), and comparing favorably to that of other lizards (including the serpentiform anguid *Pseudopus apodus*; Macrì et al., 2019).

The inner ear is composed of the semicircular canals and their ampullae, the spherical sacculus, and the endosseus cochlear duct (Fig. S7). The semicircular canals are thin, contrasting with the condition in some arboreal squamates (Palci et al., 2017). The volume bounded by the three canals is filled by the spherical sacculus (vestibule of Yi and Norell, 2015; Paluh and Bauer, 2017). This is enlarged as in fossorial or semi-fossorial extant members of Scincidae (Paluh and Bauer, 2017), Serpentes (Yi and Norell, 2015), and other extant burrowing tetrapods (e.g., Evans, 2016). Aquatic tetrapods also have large, spherical sacculi, although this condition is paired with widened semicircular canals (Palci et al., 2017). The anterior ampulla is located just anterior to the lateral ampulla and represents the anterior extent of the inner ear. The ampullae are all similar in volume. The anterior and posterior semicircular canals are arched dorsally and more widely separated from the spherical sacculus than the horizontal semicircular canal. Medially, the anterior and posterior semicircular canals form a thick region identifiable as the common crus. The endosseus cochlear duct is positioned ventral to the spherical sacculus and semicircular canals and is ventrally elongated.

VI. Extended Phylogenetic Methods.

Sampling.— In order to rigorously test the phylogenetic relationships of the new squamates, we included them in the dataset of Longrich et al. (2012), which is an updated version of Gauthier et al. (2012). We added several recently published early lacertids that would be expected to show features found in early scincoids if the sister relationship between Pan-Lacertoidea and Pan-Scincoidea (viz., a monophyletic Scincomorpha) found in morphological trees since Camp (1923) is accurate. We added the Čerňanský and Smith (2018) codings of the crown lacertid †*Gallotia atlantica* and the Paleogene stem lacertids †*Eolacerta robusta* and †*Stefanikia siderea* to this revised version of the Gauthier et al. (2012) dataset and consulted published CT scans and osteologies of these taxa (Čerňanský and Smith, 2018; Čerňanský and Syromyatnikova, 2019) to code the 12 characters added by Longrich et al. (2012). Additionally, we included

†*Cryptolacerta hassiaca* based on published codings for this species in Longrich et al. (2015) and CT scans (Müller et al., 2011). To test relationships of 'paramacellodids' among squamates, we included the best-known species referred to this group of fossils, the partial skull and skeleton of *Becklesius cataphractus* from the Early Cretaceous (Barremian) of Spain based on the literature (Richter, 1994). We did not include any of the Purbeck Formation 'paramacellodid-grade' lizards †*Paramacellodus oweni* and †*Pseudosaurillus becklesi* (Hoffstetter, 1967) or the fragmentary Cretaceous North American lizards †*Atokasaurus metarsiodon* (Nydam and Cifelli, 2002) and †*Sciroseps pawhuskai* (Saurez et al., 2021) because these species are known from isolated maxillae and dentaries that would artificially inflate uncertainty in our phylogenetic estimations. Similarly, we excluded †*Neokotus sanfransicanus* from the Early Cretaceous of South America, which has been allied with †Paramacellodidae (Bittencourt et al., 2022), as we were not able to personally examine this taxon or resolve it within Pan-Scincoidea in preliminary runs of our dataset, in large part due to missing data. We also could not personally examine the enigmatic Late Jurassic Asian taxon †*Sharovisaurus karatauensis*, which is known from a complete but poorly described skeleton that resembles pan-scincoids (Hecht and Hecht, 1984).

***Protocol.*—**The phylogenetic analysis was run without a molecular scaffold in the parsimony program TNT v. 1.5 (Goloboff and Catalano, 2016). The most completely known rhynchocephalian, *Sphenodon punctatus*, was specified as the outgroup. An initial Wagner search with default parameters for ratchet, tree fuse, drift, and sectorial search was conducted with space for 1000 replicates and followed by a round of traditional bisection-reconnection (TBR) branch swapping more than 100000 replicates to explore tree islands more fully. We also ran an additional analysis using molecular constraints for extant taxa based on a recently published phylogenetic analysis of Squamata using genome-scale sequence data (Burbrink et al., 2020). In our approach, we binned extant taxa into major clades recognized by both molecular and morphological analyses (Iguania, Gekkota, Lacertoidea, Serpentes, and Scincoidea) and constrained the relationships among them according to the molecular topology presented in Burbrink et al. (2020). We did not constrain relationships within any of those clades because of uncertainty surrounding some of them in molecular vs. morphological trees.

We also used small samples of the most complete extinct iguanians and snakes and pruned some problematic serpentiform taxa from the Longrich et al. (2012) dataset. The complete dataset used for this study is in the supplementary data.

VII. Extended Phylogenetic Results.

Three total phylogenetic analyses were run to assess the position of the new species of pan-scincoids. These included the following: runs with both taxa included with constraints enforced or unenforced, and a run with only †*Eoscincus ornatus* with constraints enforced. This taxon deletion/insertion experiment was conducted to assess whether polytomies at the base of Pan-Scincoidea were caused by character conflict or simply from missing codings due to non-preservation in either of the newly described fossils. We did not run a constrained analysis that only included the poorly known †*M. borealis* since it was clear that the unstable position of this species was driving the production of the polytomy in the constrained analysis including both new taxa. All analyses except the iteration that included both species and used a molecular constraint placed on †*M. borealis* and †*E. ornatus* within Pan-Scincoidea (Fig. S9—11). Constrained analyses generally followed the results of unconstrained analyses, and both †*E. ornatus* and †*M. borealis* were supported as pan-scincoids in strict consensus and different maximally parsimonious topologies when only one of them was included in the analysis (Fig. S11—12).

One key result was the identification of a clade containing the reasonably well-known Early Cretaceous Spanish species †*Becklesius cataphractus* and the Late Jurassic North American †*Eoscincus ornatus* in every single parsimony and Bayesian analysis conducted (Fig. S9-12), constrained or not. This clade is classically called the †Paramacellodidae (Estes, 1983) after a set of referred elements named †*Paramacellodus oweni* from the European Early Cretaceous Purbeck Formation of Great Britain (Hoffstetter, 1967; Richer, 1994). Although most were not included in this analysis, paramacellodids are currently thought to include the Early Cretaceous Spanish species attributed to †*Paramacellodus* (†*P. marocensis* and †*P. sinuosus*; Richter, 1994), the Early Cretaceous Spanish species †*Becklesius cataphractus* (Richter, 1994), possibly the Late Jurassic †*Sharovisaurus karatauensis* from Kazakhstan (Hecht and Hecht, 1984), the Early Cretaceous North American species †*Atokasaurus metarsiodon* (Nydam and Cifelli, 2002) and †*Sciroceps pawhuskai* (Suarez et al., 2021), and the oldest South American lizard †*Neokotus sanfranciscanus* from the Early Cretaceous of Brazil (Bittencourt et al., 2020) (see Comments in "Nomenclatural notes" below). Despite the fragmentary nature of the holotype specimen, †*P. oweni* shares with many of the aforementioned taxa a distinctive dental morphology described in detail by Richter (1994). Thus, we retain Paramacellodidae as the name

of a basal pan-scincoid clade containing, at the very least, †*Becklesius cataphractus* and †*Eoscincus ornatus*.

Unconstrained Analysis.

Apomorphies shared by †*Eoscincus ornatus* (bold) and †*Microteras borealis* (italicized) within major clades of Pan-Squamata (character numbers listed below correspond to those in the dataset of Gauthier et al., 2012 as modified by Longrich et al., 2012).

295. Epipterygoid:

(0) expanded dorsoventrally and ventrally.

(1) columelliform.

390. Coronoid-surangular articulation:

(0) coronoid restricted to medial aspect of mandible.

(1) coronoid extends onto dorsal surface of surangular.

(2) coronoid arches over dorsal margin of mandible to reach lateral face of surangular.

393. Coronoid, posteromedial process:

(0) absent.

(1) present.

Squamata

1. Premaxilla:

(0) paired.

(1) fused.

Scincomorpha

149. Jugal lateral exposure below orbit:

(0) absent.

(1) partly exposed above orbital margin of maxilla.

(2) entirely exposed above orbital margin of maxilla.

250. Palatine choanal fossa development:

(0) absent.

(1) present anteriorly on palatine.

(2) extending about halfway back on palatine.

(3) fully developed to end of element.

254. Palatine, shape of posterolateral margin at pterygoid suture:

(0) unmodified.

(1) palatine with discrete surface set off from choanal fold, extending along lateral margin from maxillary to pterygoid sutures.

(2) transversely broad palatine at pterygo-palatine suture strongly restricts suborbital fenestra.

399. Surangular adductor fossa on external face of mandible:

(0) shallow and extends ventrally no more than halfway down.

(1) deep and extends ventrally more than halfway down (nearly to angular bone).

Pan-Scincoidea.

149. Jugal lateral exposure below orbit:

(0) absent.

(1) partly exposed above orbital margin of maxilla.

(2) entirely exposed above orbital margin of maxilla.

†Paramacellodidae

7. Premaxilla body anterior ethmoidal foramina exit via:

(0) external naris.

(1) premaxilla notch.

(2) premaxilla body.

(3) between premaxilla and maxilla.

(4) in maxilla.

123. Maxilla suborbital process tip shape at jugal articulation$_{N}$:

(0) suborbital margin slopes smoothly to tip.

(1) with distinct step or V-shaped notch distally at jugal articulation.

255. Palatine teeth:

(0) present.

(1) absent.

Constrained Analysis.

Features uniting †*Eoscincus ornatus* (bold) and †*Microteras boreas* (italicized) with squamate ingroups:

Pan-Squamata

295. Epipterygoid:

(0) expanded dorsoventrally and ventrally.

(1) columelliform.

390. Coronoid-surangular articulation:

(0) coronoid restricted to medial aspect of mandible.

(1) coronoid extends onto dorsal surface of surangular.

(2) coronoid arches over dorsal margin of mandible to reach lateral face of surangular.

393. Coronoid, posteromedial process:

(0) absent.

(1) present.

Squamata

39. Frontal subolfactory process depth:

(0) 25–35%.

(1) 42–53%.

(2) 58–68%.

(3) 75–85%.

(4) more than 89%.

Pan-Scincoidea (*Eoscincus ornatus* only).

399. Surangular adductor fossa on external face of mandible:

(0) shallow and extends ventrally no more than halfway.

(1) deep and extends ventrally more than halfway down (nearly to angular bone).

†Paramacellodidae

144. Jugal anterior extent*:

(0) broadly separated from prefrontal.

(1) reaches level of prefrontal.

*This is a classic synapomorphy of *Pan-Scincoidea* (see Gauthier et al., 2012).

157. Jugal medial ridge:

(0) medial ridge weak, jugal lateral to ectopterygoid at base in dorsal view.

(1) medial ridge pronounced, base of medial ridge projects behind ectopterygoid base in dorsal view.

421. Dentary tooth count:

(0) 0.

(1) 4–9.

(2) 10–20.

(3) 21–35.

(4) 36 or more.

434. Cusps on posterior teeth:

(0) unicuspid.

(1) bicuspid.

(2) tricuspid.

572. Dermal skull bone ornamentation:

(0) smooth.

(1) lightly rugose about frontoparietal suture.

(2) present over dorsum.

(3) present on jugal postorbital bar.

XI. Appendix 2: Extended Taxonomic Notes.

We follow most nomenclatural rules of *PhyloCode* (de Queiroz and Cantino, 2020), and define the taxon names of focus here in terms of common-ancestry relationships as follows.

Pan-Scincoidea C. D. Brownstein, D. Meyer, M. Fabbri, B.A.S. Bhullar, and J. A. Gauthier, new clade name

Definition: The total clade of the crown clade Scincoidea. This is a crown-based total-clade definition. Abbreviated definition: total ∇ of Scincoidea.

Etymology: Derived from combining the Greek *pantos* (all), a prefix signifying reference to a total clade (pan-monophylum), plus Scincoidea, the name of its corresponding crown clade (see Scincoidea below for the etymology); thus, "Pan-Scincoidea" refers to "the total clade of Scincoidea."

Reference Phylogeny: Fig. 4A, in which specifiers for crown Scincoidea (see below) are represented on this tree by Scincidae (representing *Scincus scincus*) and Cordylidae (representing *Cordylus cordylus* plus *Gerrhosaurus flavigularis*), and stem scincoids by †*Becklesius cataphractus,* †*Eoscincus ornatus,* †*Microteras borealis,* and †*Parmeosaurus scutatus.*

Composition: Extant Scincoidea (see below) and all extinct taxa descended from its last common ancestor, as well as stem scincoids that diverged prior to the origin of the crown. Potential stem scincoids from the Late Jurassic and Early Cretaceous, most notably species often referred to Paramacellodidae Estes 1983, include †*Paramacellodus oweni,* †*Paramacellodus marocensis,* †*Paramacellodus sinuosus,* †*Becklesius cataphractus,* †*Becklesius hoffstetteri,* †*Cuencasaurus estesi,* and, according to Evans and Chure (1998), †*Mimobecklesisaurus gansuensis* (Li, 1985), and an intact, if only preliminarily described, skeleton of a species from the Jurassic of Kazakhstan, †*Sharovisaurus karatauensis* (Hecht and Hecht, 1984). Additional fragmentary remains of stem scincoids include †*Atokasaurus metarsiodon* (Nydam and Cifelli, 2002) †*Neokotus sanfranciscanus* (Bittencourt et al., 2020; see Comments below), and †*Sciroceps pawhuskai* (Suarez et al., 2021). Potential stem scincoids have been reviewed by Hoffstetter (1967), Seiffert (1973), Estes (1983), Richter (1994), Evans and Chure (1998; 1999), Gao and Norell (2000), Folie et al. (2005), Conrad (2008), Gauthier et al. (2012), Simões et al. (2018), Tałanda (2018), Alifanov (2019), and Bittencourt et al. (2020).

Diagnostic Apomorphies: As illustrated in Gauthier et al. (2012): Character 149(0) jugal not exposed below orbit; Character 254(1) palatine lateral margin set off from choanal fold by a flat, subparallel-sided surface between maxilla and pterygoid; Character 367(2) dentary coronoid process broadly overlaps anterolateral ramus of coronoid bone; Character 399(1) prominent surangular fossa for jaw muscle insertion extends ventrally more than half-way down external face of mandible.

Synonyms: Cordyloidea, approximate synonym. See also synonymy of Scincoidea below.

Comments: Aside from the serpentiform *Chamaesaura* (originally *Lacerta*) *anguina* Linnaeus 1758, few Cordylidae (= Cordylinae + Gerrhosaurinae) were known to pre-Darwinian biologists.

Fitzinger (1826), for example, coined the name Cordyloidea for a "family" containing only *Cordylus varus* (= *C. cordylus*) and *Chamaesaura anguina*; gerrhosaurines had yet to be recognized (e.g., *Gerrhosaurus flavigularis* Wiegmann 1828). To complicate matters, Fitzinger (1826: p.18) also included an Australian skink, *Trachydosurus* (= *Tiliqua rugosa*) and a South American gymnophthalmid (*Leposoma*) in his Cordyloidea. Neither of these anomalous assignments were, however, repeated among his "cordyloids" on page 50 of that same publication, where he included only *Cordylus* and *Chamaesaura*. Fitzinger (1843) later separated *Chamaesaura* (Tribe Chamaesauri) from *Cordylus* (Tribe Ptychopleurae) within his Section Cyclosauri, to which he added some gerrhosaurine cordylids (*Zonurus*, *Gerrhosaurus*), as well as some anguids (*Gerrhonotus*) and scincids (*Chalcides*). Indeed, until the landmark study of McDowell and Bogert (1954), few herpetologists considered *Gerrhosaurus* to be particularly closely related to *Cordylus* (e.g., Camp, 1923). Large-scale and explicitly phylogenetic analyses of morphology later corroborated McDowell and Bogert's insight (e.g., Estes et al., 1988; Lang, 1991; Evans, 2003; Gauthier et al., 2012; but see Conrad, 2008, and Simões et al., 2018, for an alternative view), as have all phylogenetic analyses of DNA-sequence data from Frost et al. (2001) to Burbrink et al. (2020).

"Open" circumscription permitted by traditional nomenclatural systems promotes imprecision and thereby confusion by failing to precisely and consistently distinguish crown- from stem-members of the same total clade (e.g., Squamata, Squamata-1, and Squamata-3 of Simões et al., 2018). Indeed, neontologists often associate taxon names with crown clades (e.g., Cordyloidea *sensu* Burbrink et al., 2020) that paleontologists often prefer to extend further down into the total clade (e.g., Cordyloidea *sensu* Estes, 1983) (see de Queiroz and Gauthier, 1990, 1992; Rowe and Gauthier, 1992). In no case, however, has Scincidae ever been included in Cordyloidea.

A Google Scholar search (accessed June 2021) yielded ~2,100 results for Cordylidae, ~183 for Cordyliformes, and only ~25 for Cordyloidea. The first of these names has been used for two nested clades. Prior to McDowell and Bogert (1954), it was often used for a less inclusive clade containing just *Cordylus* and its nearest relatives (= "Zonuridae" of Gray, 1837—and the influential work of Boulenger, 1885—was not based on the type genus, *Cordylus*, and was subsequently replaced by Mertens' "Cordylidae" in 1937). Thereafter, Cordylidae has also been used for a more inclusive clade containing both the "*Cordylus*" and "*Gerrhosaurus*"

subclades. Thus, the former clade was referred to as Cordylinae by Romer (1956) and the latter as Gerrhosaurinae by Estes (1962). The name Cordylidae Mertens 1937 (*non* Gray 1837) was first defined phylogenetically by Estes et al. (1988). Lang (1991) identified and named putative subclades within each of these clades but preferred to preserve the familiar names Cordylidae and Gerrhosauridae, so he proposed an objective synonym (Cordyliformes) for the clade Estes et al. (1988) had already tied to Cordylidae. Although the former has priority, neither taxon-name definition conforms to *PhyloCode*, which requires individual species, rather than more inclusive taxa, as specifiers (de Queiroz and Cantino, 2020). The name Cordyloidea has seldom been used in the past 195 years and, not surprisingly, has never been defined phylogenetically. Given these circumstances, we follow the proposal of Estes et al. (1988), effectively emending their definition by substituting the species *Cordylus cordylus* for Cordylinae and *Gerrhosaurus flavigularis* for Gerrhosaurinae.

We follow Estes et al. (1988) in using the name Scincoidea for the crown (see below) and, in the interests of developing a comprehensive nomenclatural system (de Queiroz and Cantino, 2020), accordingly propose Pan-Scincoidea for its corresponding total clade. It could, however, be argued that Cordyloidea would be more appropriate for this total clade (despite the fact that Scincidae has never been included within Cordyloidea). That is because Mesozoic lizards known as paramacellodids have been loosely allied to extant *Cordylus* and *Gerrhosaurus* since Hoffstetter (1967; see also Seiffert, 1973, and Estes, 1983). Mainly because at least some of them possess rectangular, 'compound' body osteoderms, that-is-to-say, a mosaic pattern of separate scale-shaped ossifications joined together within each epidermal scale (at least ventrally on the body) that are prone to disarticulate upon maceration (e.g., Richter, 1994). The "cordyloid" connection depends mainly on the morphology of the body osteoderms associated with †*Paramacellodus oweni* (Hoffstetter, 1967) and †*Becklesius cataphractus* (Richter, 1994). The entire body and skull of the intact skeleton of the Late Jurassic species †*Sharovisaurus karatauensis* are completely encased in osteoderms (Hecht and Hecht, 1984). However, it is unclear if any are 'compound' in structure, and the tooth crowns have yet to be described in detail. According to Gauthier et al. (2012), a potential stem species from the Late Cretaceous of Mongolia, †*Parmeosaurus scutatus* (Gao and Norell, 2000), is inferred to have compound body osteoderms investing the ventral body scales, at least some caudal scales, and there appears to be a mosaic of osteoderms within the cephalic scales overlying the skull roof and between the lower

jaws. At least one compound osteoderm is associated with another potential pan-cordylid from the Late Cretaceous of Madagascar, †*Konkasaurus mahalana* (Krause et al., 2003). But most of its body osteoderms are single (non-compound), as is commonly the case in cordylines (except for compound osteoderms between the lower jaws), as well as on the dorsum in many gerrhosaurines (Gauthier et al., 2012). Nevertheless, the basal crown gerrhosaurine †*Cordylosaurus subtesselatus* (Stanley, 2013) is completely enclosed in compound body osteoderms, both dorsally and ventrally (Gauthier et al., 2012). Scincidae also possesses compound osteoderms (e.g., Greer, 1970), not only covering the entire body, but also the skull (though the latter may fuse indistinguishably in adults; Gauthier et al., 2012). But scincids are conspicuous among scincoids for having thin, smooth, cycloid body scales, a form otherwise approached only on the throat in gerrhosaurines. That may explain why paleontologists have often focused on the rectangular osteoderms shared by paramacellodids and cordylids. However, that morphology appears to represent the ancestral condition within pan-scincoids, with the thin cycloid scales of scincids being derived from that (Gauthier et al., 2012). Molecular (e.g., Vicario et al., 2003; Vidal and Hedges, 2005) and morphological (e.g., Gauthier et al., 2012) studies have concluded that Xantusiidae and Cordylidae are sisters, with Scincidae as sister to that clade. If body osteoderms arose in paramacellodids prior to the origin of crown Scincoidea, then two crown clades that dwell within confined spaces, the basal cordyline *Platysaurus* and the cordylid sister, Xantusiidae, must have lost their body osteoderms secondarily and independently of one another. It may be noteworthy in this regard that the degree of osteoderm development, and their distribution on the body, correlate with habitat preferences among Cordylinae (Stanley, 2013).

†Paramacellodidae has been reported nearly world-wide from the Middle Jurassic to Early Cretaceous (e.g., Evans and Chure, 1999; Bittencourt et al., 2020). In our view, the name "Paramacellodidae" is perhaps best reserved for those taxa with diagnostic tooth crowns bearing a distinct, lingually offset, narrow-based cuspule connected by a crest to the labial apex of the cutting edge of the crown (i.e., Richter, 1994, fig. 2; Evans and Chure, 1999, fig. 1). Bittencourt et al. (2020) proposed some additional potential apomorphies of paramacellodid teeth, including labiolingual expansion at the tooth bases and a moderately developed concavity on the lingual face of the tooth crowns. At this point, we only note that lingually expanded tooth bases are also present in some extant scincids (e.g., *Sphenomorphus solomonis*) and cordylines (e.g., *Smaug*

mossambicus), although admittedly not to the degree seen in Early Cretaceous †*Atokasaurus metarsiodon* (Nydam and Cifelli, 2002). Slightly offset lingual cusps are also present in some scincoids, including Late Cretaceous to Early Paleogene stem xantusiids known as Contogeniidae (Nydam and Fitzpatrick, 2009), the Paleogene xantusiid †*Palaeoxantusia fera* (pers. obs.), and the Neogene cordyline †*Palaeocordylus bohemicus* (Čerňanský, 2012). However, these cusps are neither as offset nor as narrow-based, nor are they connected to the apical cutting edge by a crest as in paramacellodids *sensu stricto*. Interestingly, a moderately developed medial concavity at the tooth crown, together with cutting edges arising slightly lingually before ascending along the rostral and caudal edges to the apex of the crown, and a slight lingual inflection of the dentary tooth crowns, as well as medially striated tooth crowns, all appear to be generally distributed among scincoids (e.g., Thorn et al., 2021: Fig 12), and not just in paramacellodids. Tooth crowns are known to reflect dietary preferences (e.g., Hotton, 1955), and tooth-bearing bones attributed to †Paramacellodidae display the typical range of morphologies, with accessory cusps on more posterior teeth varying from strong, to weak, to absent, with their apices varying from being more bluntly pointed with distinct shoulders, to more obtusely pointed, to sharply pointed (e.g., Richter, 1994). These and other features associated with paramacellodid dentitions require more thorough study of tooth morphology in fossil and Recent scincoids. Lest †Paramacellodidae becomes a waste basket for mid-Mesozoic stem scincoids, however, greater care should be taken before referring isolated tooth-bearing bones to †Paramacellodidae on the basis of features that could be ancestral for all Pan-Scincoidea.

Gauthier et al. (2012) used "*Paramacellodus*" for a composite taxon based primarily on the stem-scincoid here named †*Eoscincus ornatus*. But in a few instances, anatomical features not preserved in †*E. ornatus* were coded based on an additional specimen, DINO 15914, that Evans and Chure (1998) referred to †*Paramacellodus* sp. cf. †*P. oweni*. However, closer study suggests that DINO 15914 may not be a stem scincoid (Meyer et al., in prep.). According to Evans and Chure (1999 fig. 1D), †*E. ornatus* possesses tooth crowns like those here attributed to †Paramacellodidae. However, it does not appear to have separate osteoderms in the skin, as it is sufficiently intact that at least a few body osteoderms might reasonably be expected to have been preserved if present. Gauthier et al. (2012) scored this species as "unknown" for this character, but we here consider body osteoderms as being "absent" to investigate the consequences of these

alternatives state assignments on tree topology: there were none. It remains possible, however, that the body osteoderms of †*E. ornatus* were just not as developed as in, for example, †*P. oweni*. Given their compound nature, such body osteoderms could have disintegrated (e.g., as in †*Yabeinosaurus*; Evans et al., 2005) and/or lost, as is often the case in fossils of crown Scincidae (pers. obs., JAG), and is apparently the case in stem scincids such as Globauridae (Gauthier et al., 2012).

Scincoidea L. I. Fitzinger 1826 [C. D. Brownstein, D. Meyer, M. Fabbri, B.A.S. Bhullar, & J. A. Gauthier], converted clade name

Definition: The least inclusive crown clade containing *Scincus* (originally *Lacerta*) *scincus* Linnaeus 1758, *Cordylus* (originally *Lacerta*) *cordylus* Linnaeus 1758, and *Gerrhosaurus flavigularis* Wiegmann 1828, but not *Lacerta agilis* Linnaeus 1758 (Lacertidae), or *Anguis fragilis* Linnaeus 1758, or *Gekko* (originally *Lacerta*) *gecko* Linnaeus 1758. This is a minimum-crown-clade definition with external specifiers. Abbreviated definition: min crown ∇ *Scincus scincus* Linnaeus 1758 & *Cordylus cordylus* Linnaeus 1758 & *Gerrhosaurus flavigularis* Wiegmann 1828 ~ *Lacerta agilis* Linnaeus 1758 & *Anguis fragilis* Linnaeus 1758 & *Gekko gecko* Linnaeus 1758.

Etymology: Derived from *scincus* (Latin), adopted from *scincos* (Ancient Greek; see also the Arabic *askincor* and Middle French *scinc*), sometimes misread as "*stincus*", especially prior to the 18th century (hence, *Lacerta stincus* in Linnaeus, 1758) plus -*oidea* (Ancient Greek) meaning "similar to".

Reference Phylogeny: Fig. 4A, in which *Scincus scincus* is represented by Scincidae, and *Cordylus cordylus* plus *Gerrhosaurus flavigularis* are represented by Cordylidae.

Composition: Scincoidea by definition includes all 1,852 extant species that the *Reptile Data Base* (http://www.reptile-database.org/) assigns to the taxon names Scincidae (1,746 spp.), Cordylidae (68 spp.), and Gerrhosauridae (38 spp.)(Reptile database accessed July 26, 2022). It

also includes all extinct members of those crown clades plus their extinct stem members, such as the stem scincids †*Scincoideus haininensis* (Folie et al., 2005) and †Globauridae (Gauthier et al., 2012) †*Yabeinosaurus tenuis* (Tałanda, 2018), and the pan-cordylid †*Konkasaurus mahalana* (Krause et al., 2003). Four additional major crown clades of Squamata might also be scincoids, depending on the phylogenetic hypothesis favored (see Comments below): Xantusiidae (34 spp.), Dibamidae (24 spp.), Amphisbaenia (194 spp.), and Serpentes (3,691 spp.).

Diagnostic Apomorphies: As illustrated in Gauthier et al. (2012): Character 95(1) parietal postparietal projection on midline; Character 108(1) parietal epipterygoid process present; Character 123(1) maxilla suborbital process caudal tip with distinct step or V-shaped notch at jugal articulation; Character 170(2) supratemporal entirely lateral to parietal supratemporal process; Character 369(1) dentary angular process extends posterior to coronoid apex on lateral face of mandible; Character 375(2) splenial anterior extent 2/3 or less of dentary tooth-row length.

Synonyms: Cordyloidea sensu Estes (1983), approximate and partial synonym. See discussion under Pan-Scincoidea above.

Comments: *Lacerta stincus* (sic) Linnaeus 1758—a common misreading prior to the 18th century later emended to *Lacerta scincus* by Shaw and Nodder (1812) - is the first-listed taxon assigned by the nominate author (Fitzinger, 1826) to his Scincoidea (as *Scincus officinalis* Laurenti 1768*,* known as the "pharmaceutical skink" since antiquity for its reputed medicinal properties, e.g., *Naturalis Historia* by Pliny the Elder, translated by Bostock and Riley, 1855). Nearly all 22 species that Fitzinger (1826) included in his Scincoidea are now regarded as members of Scincidae Gray 1825, aside from *Pygodactylus gronovii,* a serpentiform diploglossine anguid now known as *Ophiodes striatus*. Fitzinger also referred Linnaeus's (1758) *Anguis meleagris* to his Anguinoidea, although it is now thought be a serpentiform skink, *Acontias meleagris* (although often in its own "family" owing to its profound modifications for fossoriality). Similarly, taxa currently referred to Scincidae were parceled among two separate groups by Oppel (1811): Scincoides and Chalcidici. Both taxa contain not only skinks, but also distantly allied lizards, such as serpentiform anguids *Pseudopus* (Sheltopusik) and *Anguis* in the

former case, and a skink plus some serpentiform amphisbaenians and another serpentiform anguid (*Ophisaurus*) in the latter. Such complications illustrate how long-bodied, limb-reduced, snake-like, (more or less) fossorial lizards have muddled ideas about squamate phylogeny and taxonomy over the centuries, at least in morphological analyses.

Google Scholar (accessed in June, 2021), lists about 19,400 "results" when searching for Scincidae, 2,340 for Scincoides, and 540 for Scincoidea. The most widely used name Scincidae Gray 1825 is probably best reserved for the crown with which has long been associated, even if imprecisely (viz., did that name refer to the crown, particular apomorphies, or to the total clade?). Scincoides Opell 1811 is the next most common name in the literature that could be associated with this clade. But in virtually every instance, that name has been used for a single species, *Tiliqua scincoides*. Following Estes et al. (1988), we therefore elect to convert the name Scincoidea for this clade, but we attribute it to Fitzinger (1826) rather than Oppel (1811). We note that both Scincoides Oppel 1811 and Scincoidea Fitzinger 1826 would now refer to polyphyletic taxa if conceptualized with reference to taxa referred to them by their nominate authors. We cannot be sure of the intent of either Oppel or Fitzinger; did they mean to apply these names to a particular collection of taxa or to particular anatomical traits? As Joyce et al. (2004) observed, we can only be sure of how they spelled the taxon names they coined, not how they conceptualized the taxa to which they applied those names (especially in the pre-Darwian era).

Estes et al. (1988) were the first to propose a phylogenetic definition for the taxon name Scincoidea: "[t]he most recent common ancestor of Scincidae and Cordylidae and all of it descendants" (p. 217). This definition is unsuitable on several counts. First, *PhyloCode* (de Queiroz and Cantino, 2020) requires species as specifiers; we accordingly recommend substituting individual species to represent the more inclusive taxon names used by Estes et al. (1988): *Scincus scincus* for Scincidae, and *Cordylus cordylus* (Cordylinae) plus *Gerrhosaurus flavigularis* (Gerrhosaurinae) for Cordylidae as used by those authors (some have used Cordyliformes Lang 1991 for the clade composed of Cordylidae plus Gerrhosauridae, e.g., Gauthier et al., 2012; others have used the name Cordyloidea to approximate this clade, e.g., Burbrink et al., 2020; see Comments in Pan-Scincoidea above). Second, Oppel (1811) appears to use the Latin (and Old French) vernacular term, viz., "scincoides", for his taxon, whereas *PhyloCode* prefers conversion of formally Latinized, more obviously non-vernacular taxon

names, such as Scincoidea. Finally, we prefer the slightly modified version of the definition proposed by Conrad (2008), as it buffers the definition of Scincoidea to ensure that Scincidae, Cordylinae and Gerrhosaurinae will always be included within it, following Estes et al. (1988). Conrad's version, which includes external specifiers that cannot, by definition, be included in Scincoidea, also leaves open the possibility that taxa of more-or-less uncertain placement, including Xantusiidae, Dibamidae, Amphisbaenia, and Serpentes, might eventually be included in Scincoidea should they root within the clade bracketed by the last common ancestor of *S. scincus*, *C. cordylus* and *G. flavigularis*. At present, only Xantusiidae would be regarded as a member of Scincoidea based on comprehensive morphological (e.g., Gauthier et al., 2012) and molecular (e.g., Burbrink et al., 2020) phylogenetic analyses.

Supplementary References.

- Alifanov, V. R. 2019. Lizards of the families Eoxantidae*,* Ardeosauridae*,* Globauridae*,* and Paramacellodidae (Scincomorpha) from the Aptian-Albian of Mongolia*.* Paleontological Journal. 53(1): 74–88.
- Allemand, R, Boistel, R, Daghfous, G, Blanchet, Z, Cornette, R, Bardet, N, Vincent, P, and Houssaye, A. 2017. Comparative morphology of snake (Squamata) endocasts: evidence of phylogenetic and ecological signals. *Journal of Anatomy* **231**(6):849-868.
- Ash SR, and Tidwell WD. 1998. Plant megafossils from the Brushy Basin Member of the Morrison Formation near Montezuma creek Trading Post, southeastern Utah. *Modern Geology* **22**: 321.
- Bates MF, and Stanley EL. 2020. A taxonomic revision of the south-eastern dragon lizards of the *Smaug warreni* (Boulenger) species complex in southern Africa, with the description of a new species (Squamata: Cordylidae). *PeerJ* **8**:e8526.
- Bell, CJ, Mead JI, and Swift SL. 2009. Cranial osteology of *Moloch horridus* (Reptilia: Squamata: Agamidae). *Records of the Western Australian Museum* **25**:201-237.
- Bertog J, Jeffery DL, Coode K, Hester WB, Robinson RR, Bishop J, and Electronica P. 2014. Taphonomic patterns of a dinosaur accumulation in a lacustrine delta system in the Jurassic Morrison Formation, San Rafael Swell, Utah, USA. *Palaeontologia Electronica* **17**(3):1-19.

- Bever GS, Bell CJ, and Maisano JA. 2005. The ossified braincase and cephalic osteoderms of *Shinisaurus crocodilurus* (Squamata, Shinisauridae). *Palaeontologia Electronica* **8**(1):1-36.
- Bittencourt JS, Simões TR, Caldwell MW, and Langer MC. 2020. Discovery of the oldest South American fossil lizard illustrates the cosmopolitanism of early South American squamates. *Communications Biology* **3**(1):1-11.
- Bolet A, Delfino M, Fortuny J, Almécija S, Robles JM, and Alba DM. 2014. An amphisbaenian skull from the European Miocene and the evolution of Mediterranean worm lizards. *PLoS ONE* **9**(6):e98082.
- Borsuk-Białynicka, M. 1985. Carolinidae, a new family of xenosaurid-like lizards from the Upper Cretaceous of Mongolia. *Acta Palaeontologica Polonica* **30**(3-4):151-176.
- Borsuk-Białynicka, M. 1988. *Globaura venusta* gen. et sp. n. and *Eoxanta lacertifrons* gen. et sp. n.—non-teiid lacertoids from the Late Cretaceous of Mongolia. *Acta Palaeontologica Polonica* **33**:211–248.
- Bostock, J., and H. T. Riley. 1855. The Natural History, Pliny the Elder. Book VIII: The Nature of the Terrestrial Animals (chapter 38). Taylor and Francis, London.
- Boulenger, G. A. 1885. Catalogue of the lizards in the British Museum (Natural History), Volume 2. Trustees of the British Museum, London.
- Brykczynska U, Tzika AC, Rodriguez I, and Milinkovitch MC. 2013. Contrasted evolution of the vomeronasal receptor repertoires in mammals and squamate reptiles. *Genome Biology and Evolution* **5**(2):389-401.
- Burbrink FT, Grazziotin FG, Pyron RA, Cundall D, Donnellan S, Irish F, Keogh JS, Kraus F, Murphy RW, Noonan B, and Raxworthy CJ. 2020. Interrogating genomic-scale data for Squamata (lizards, snakes, and amphisbaenians) shows no support for key traditional morphological relationships. *Systematic Biology* **69**(3):502-520.
- Caldwell MW, Nydam RL, Palci A, and Apesteguía S. 2015. The oldest known snakes from the Middle Jurassic-Lower Cretaceous provide insights on snake evolution. *Nature Communications* **6**(1):1-11.
- Camaiti M, Villa A, Wencker LC, Bauer AM, Stanley EL, and Delfino M. 2019. Descriptive osteology and patterns of limb loss of the European limbless skink *Ophiomorus punctatissimus* (Squamata, Scincidae). *Journal of Anatomy* **235**(2):313-345.

- Caputo V. 2004. The cranial osteology and dentition in the scincid lizards of the genus *Chalcides* (Reptilia, Scincidae). *Italian Journal of Zoology* **71**(2):35-45.
- Čerňanský A, and Syromyatnikova EV. 2019. The first Miocene fossils of *Lacerta cf. trilineata* (Squamata, Lacertidae) with a comparative study of the main cranial osteological differences in green lizards and their relatives. *PloS ONE* **14**(8):e0216191.
- Čerňanský A, Bolet A, Müller J, Rage JC, Augé M, and Herrel A. 2017. A new exceptionally preserved specimen of *Dracaenosaurus* (Squamata, Lacertidae) from the Oligocene of France as revealed by micro-computed tomography. *Journal of Vertebrate Paleontology* **37**(6):e1384738.
- Čerňanský, A., Stanley, E.L., Daza, J.D., Bolet, A., Arias, J.S., Bauer, A.M., Vidal-García, M., Bevitt, J.J., Peretti, A.M., Aung, N.N. and Evans, S.E. 2022. A new Early Cretaceous lizard in Myanmar amber with exceptionally preserved integument. *Scientific Reports* **12**(1):1-12.
- Čerňansky, A, and Smith, KT. 2018. Eolacertidae: a new extinct clade of lizards from the Palaeogene; with comments on the origin of the dominant European reptile group– Lacertidae. *Historical Biology* **30**(7):994-1014.
- Čerňanský, A. 2012. The oldest known European Neogene girdled lizard fauna (Squamata, Cordylidae), with comments on Early Miocene immigration of African taxa. Geodiversitas 34 (4): 837-848.
- Christiansen EH, Kowallis BJ, Dorais MJ, Hart GL, Mills CN, Pickard M, Parks E. 2015. The record of volcanism in the Brushy Basin Member of the Morrison Formation: Implications for the Late Jurassic of western North America. *Geological Society of America Special Paper* **513**:399-439.
- Conrad JL and Norell MA. 2010. Cranial Autapomorphies in Two Species of *Iguana* (Iguanidae: Squamata). *Journal of Herpetology* **44**(2):307-312.
- Conrad JL, and Daza JD. 2015. Naming and rediagnosing the Cretaceous gekkonomorph (Reptilia, Squamata) from Öösh (Övörkhangai, Mongolia). *Journal of Vertebrate Paleontology* **35**(5):e980891.
- Conrad JL. 2008. Phylogeny and systematics of Squamata (Reptilia) based on morphology. *Bulletin of the American Museum of Natural History* **310**:1-182.

- Cooley JT, and Schmidt JG. 1998, An anastomosed fluvial system in the Morrison Formation (Upper Jurassic) of southwest Montana. *Modern Geology* **22**:171-208.
- Cruzado-Caballero P, Ruiz CC, Bolet A, Colmenero JR, De la Nuez J, Casillas R, Llacer S, Bernardini F, and Fortuny J. 2019. First nearly complete skull of *Gallotia auaritae* (lower-middle Pleistocene, Squamata, Gallotiinae) and a morphological phylogenetic analysis of the genus Gallotia. *Scientific Reports* **9**(1):1-14.
- Currie BS. 1998. Upper Jurassic–Lower Cretaceous Morrison and Cedar Mountain Formations, NE Utah–NW Colorado: relationships between nonmarine deposition and early Cordilleran foreland-basin development: *Journal of Sedimentary Research* **68**:632-652.
- Daza JD, Alifanov VR, and Bauer AM. 2012. A redescription and phylogenetic reinterpretation of the fossil lizard *Hoburogekko suchanovi* Alifanov, 1989 (Squamata, Gekkota), from the Early Cretaceous of Mongolia. *Journal of Vertebrate Paleontology* **32**(6):1303-1312.
- Daza JD, Bauer AM, and Snively E. 2013. *Gobekko cretacicus* (Reptilia: Squamata) and its bearing on the interpretation of gekkotan affinities. *Zoological Journal of the Linnean Society* 167(3):430-448.
- de Queiroz, K. and J. Gauthier, 1990. Phylogeny as a central principle in taxonomy: phylogenetic definitions of taxon names. Systematic Zoology, 39(4):307-322.
- de Queiroz, K. and J. Gauthier, 1992. Phylogenetic taxonomy. Annual Review of Ecology and Systematics, 23:449-480.
- de Queiroz, K., and P. D. Cantino. 2020. International Code of Phylogenetic Nomenclature (PhyloCode). CRC Press, Boca Raton, FL.
- DeCelles PG. 2004. Late Jurassic to Eocene evolution of the Cordilleran thrust belt and foreland basin system, western U.S.A. *American Journal of Science* **304**:105-168.
- DeMar Jr, DG *et al.* 2017. A new Late Cretaceous iguanomorph from North America and the origin of New World Pleurodonta (Squamata, Iguania). *Proc. Roy. Soc. B* **284**(1847): 20161902.
- Dong L, Xu X, Wang Y, and Evans SE. 2018. The lizard genera *Bainguis* and *Parmeosaurus* from the Upper Cretaceous of China and Mongolia. *Cretaceous Research* **85**:95-108.

- Estes, R. 1983. Sauria terrestria, Amphisbaenia. Handbuch der Paläoherpetologie. Teil 10A. Gustav Fischer, Stuttgart.
- Estes, R. 1962. A fossil gerrhosaur from the Miocene of Kenya (Reptilia: Cordylidae). Breviora 158:1-10.
- R. Estes. 1969. A scincoid lizard from the Cretaceous and Paleocene of Montana.
- *Breviora* **331**, 1-9.
- Estes, R., K. de Quieroz, and J. Gauthier. 1988. Phylogenetic relationships within Squamata. Pp. 119–281 *in* Phylogenetic Relationships of the Lizard Families, R. Estes and G. Pregill (eds.). Stanford University Press, Stanford, CA.
- Evans SE, and Chure DC. 1998. Paramacellodid lizard skulls from the Jurassic Morrison Formation at Dinosaur National Monument, Utah. *Journal of Vertebrate Paleontology* **18**(1):99-114.
- Evans SE, and Chure DJ. 1999. Upper Jurassic lizards from the Morrison Formation of Dinosaur National Monument. In: Gillette DD, ed. Vertebrate Paleontology in Utah. *Miscellaneous Publications of the Utah Geological Survey* **99**(1):151–159.
- Evans SE, Raia P, and Barbera C. 2006. The lower Cretaceous lizard genus *Chometokadmon* from Italy. *Cretaceous Research* 27(5):673-683.
- Evans SE, Wang Y, and Li C. 2005. The Early Cretaceous lizard genus *Yabeinosaurus* from China: resolving an enigma. *Journal of Systematic Palaeontology* **3**(4):319-335.
- Evans SE. 1980. The skull of a new eosuchian reptile from the Lower Jurassic of South Wales. *Zoological journal of the Linnean Society* **70**(3):203-264.
- Evans SE. 1994. A new anguimorph lizard from the Jurassic and Lower Cretaceous of England. *Palaeontology* **37**(1):33-49.
- Evans, S. E. 2003. At the feet of the dinosaurs: the early history and radiation of lizards.
- Evans, S. E., and D. C. Chure, 1999. Upper Jurassic lizards from the Morrison Formation of Dinosaur National Monument, Utah. Vertebrate Paleontology in Utah. Utah Geological Survey Misc. Pubs.:151-159.
- Evans, S. E., and D. C. Chure. 1998. Paramacellodid lizard skull from the Jurassic Morrison Formation at Dinosaur National Monument, Utah. Journal of Vertebrate Paleontology 18:99–114.

- Evans, S. E., Y. Wang, and C. Li, 2005. The Early Cretaceous lizard *Yabeinosaurus* from China: resolving an enigma. Journal of Systematic Palaeontology 3:319–335.
- Evans, SE. 2016. The lepidosaurian ear: variations on a theme. In: *Evolution of the Vertebrate Ear.* Springer: Cham. pp. 245-284.
- Fitzinger, L. 1843. Systema reptilium. Fasiculus Primus, *Amblyglossae*. Braumüller et Seidel, Vindobonae.
- Fitzinger, L. I. 1826. Neue Classification der Reptilien nach ihren natürlichen Verwandtschaften. Nebst einer Verwandtschafts-Tafel und einem Verzeichnisse der Reptilien-Sammlung des K. K. Zoologischen Muesum's zu Wien. J. G. Heubner, Vienna.
- Folie A, Sigé B, and Smith T. 2005. A new scincomorph lizard from the Palaeocene of Belgium and the origin of Scincoidea in Europe. *Naturwissenschaften* **92**(11):542-546.
- Foster JR. 2003. Paleoecological Analysis of the Vertebrate Fauna of the Morrison Formation (Upper Jurassic), Rocky Mountain Region, USA. *Bulletin of the New Mexico Museum of Natural History and Science* **23**(23):1-95.
- Frost, D. R., D. Janies, P. le F. N. Mouton, T. A. Titus. 2001. A molecular perspective on the phylogeny of the girdled lizards (Cordylidae, Squamata). American Museum Novitates 3310:1-10.
- Gao K, and Norell MA. 1998. Taxonomic revision of *Carusia* (Reptilia, Squamata) from the late Cretaceous of the Gobi Desert and phylogenetic relationships of anguimorphan lizards. *American Museum Novitates* **3230**:1—51.
- Gao K, and Norell, MA. 2000. Taxonomic composition and systematics of Late Cretaceous lizard assemblages from Ukhaa Tolgod and adjacent localities, Mongolian Gobi Desert. *Bulletin of the American Museum of Natural History* **249**:1-118.
- Gauthier J, Estes R, and de Queiroz K. 1988. A phylogenetic analysis of Lepidosauromorpha. *Phylogenetic Relationships Of The Lizard Families.* pp. 15-98.
- Gauthier J. 1982. Fossil xenosaurid and anguid lizards from the early Eocene of Wyoming, and a revision of the Anguioidea. *University of Wyoming Contributions to Geology* 21(1):7-54.
- Gauthier JA, Kearney M, Maisano JA, Rieppel O, and Behlke AD. 2012. Assembling the squamate tree of life: perspectives from the phenotype and the fossil record. *Bulletin of the Peabody Museum of Natural History* **53**(1):3-308.

- Gelnaw WB. 2011. On The cranial osteology of *Eremiascincus* and its use for identification. Ms. Thesis, East Tennessee State University.
- Gilmore CW. 1928. Fossil lizards of North America. *National Academy of Sciences Memoirs* 22(3):1-210.
- Goloboff P, Catalano S. 2016. TNT version 1.5, including full implementation of phylogenetic morphometrics. *Cladistics* **32**(3):221-238.
- Gorman II MA, Miller IM, Pardo JD, and Small BJ. 2008. Plants, fish, turtles, and insects from the Morrison Formation: A Late Jurassic ecosystem near Cañon City, Colorado. *Roaming the Rocky Mountains and Environs: Geological Field Trips* 10:295-311.
- Gray, J. E. 1825. A synopsis of the genera of Reptiles and *Amphibia*, with a description of some new species. Annals of Philosophy 10:193–217.
- Gray, J. E. 1837. General arrangement of the Reptilia. Proceedings of the Zoological Society of London 1837(5):131-132.
- Greer AE. 1970. A subfamilial classification of scincid lizards. *Bulletin of the of the Museum of Comparative Zoology* **139**:151– 183.
- Greer, A. 1985. The relationships of the lizard genera *Anelytropsis* and *Dibamus,* J. Herpetol. **19**(1):116-156.
- Hecht MK, Hecht BM. 1984. A new lizard from Jurassic deposits of Middle Asia. *Palaeontological Journal* **18**(3):133–136.
- Hernández Morales C, Peloso PLV, García WB, and Daza JD. 2019. Skull morphology of the lizard *Ptychoglossus vallensis* (Squamata: Alopoglossidae) with comments on the variation within Gymnophthalmoidea. *The Anatomical Record* **302**(7):1074-1092.
- Hoffstetter R. 1967. Coup d'œil sur les sauriens (= Lacertiliens) des couches de Purbeck (Jurassique Supérieur d'Angleterre). *Colloques Internationaux du Centre National de la Recherche Scientifique* 163:349-371.
- Hoffstetter, R. 1967. Coup d'œil sur les Sauriens (=Lacertiliens) des couches de Purbeck.
- Hotton, N., 1955. A survey of adaptive relationships of dentition to diet in the North American Iguanidae. American Midland Naturalist 55(1):88-114**.**

- Hutchinson MN, Robertson P, and Rawlinson PA. 1989. Redescription and ecology of the endemic Tasmanian scincid lizards *Leiolopisma microlepidotum* and *L. pretiosum*. *Papers and Proceedings of the Royal Society of Tasmania* **123**:257-274.
- Joyce, W. G., J. F. Parham, J. A. Gauthier 2004. Developing a protocol for the conversion of rank-based taxon names to phylogenetically defined clade names, as exemplified by turtles. Journal of Paleontology 78(5):989-1013.
- Kearney M, Maisano JA, and Rowe T. 2005. Cranial anatomy of the extinct amphisbaenian *Rhineura hatcherii* (Squamata, Amphisbaenia) based on high-resolution X-ray computed tomography. *Journal of Morphology* **264**(1):1-33.
- Kearney M. 2003. Systematics of the Amphisbaenia (Lepidosauria: Squamata) based on morphological evidence from recent and fossil forms. *Herpetological Monographs* **17**(1):1-74.
- Kirkland JI. 1998. Morrison fishes. *Modern Geology* **22**(1):1-4.
- Klembara J, Dobiašová K, Hain M, and Yaryhin O. 2017. Skull anatomy and ontogeny of legless lizard *Pseudopus apodus* (Pallas, 1775): heterochronic influences on form. *Anatomical Record* **300**(3):460-502.
- Kowallis BJ, Britt BB, Greenhalgh BW, and Sprinkel DA. 2007. New U-Pb zircon ages from an ash bed in the Brushy Basin Member of the Morrison Formation near Hanksville, Utah. In: *Utah Geological Association Central Utah: Diverse Geology of a Dynamic Landscape*. pp. 75-80.
- Kowallis BJ, Christiansen EH, and Deino AL. 1991. Age of the Brushy Basin Member of the Morrison Formation, Colorado Plateau, western USA. *Cretaceous Research* **12**(5):483-493.
- Kraus, D. W., S. E. Eavns, and K. Q. Gao. 2003. First definitive record of Mesozoic lizards from Madagascar. Journal of Vertebrate Paleontology 23:842-856.
- Lang, M. 1991. Generic relationships within Cordyliformes (Reptilia: Squamata). Bulletin de l'Institut Royal des Sciences Naturelles de Belgique 61:121–188.
- Lara MB, Foster JR, Kirkland JI, and Howells TF. 2020. First fossil true water bugs (Heteroptera, Nepomorpha) from Upper Jurassic strata of North America (Morrison Formation, southeastern Utah). *Historical Biology*, 1-9.

- Laurenti, J. N. 1768. Specimen medicum, exhibens synopsin reptilium emendatam cum experimentis circa venena et antidota reptilium austriacorum. Joan. Thom. Nob. De Trattnern, Viennae.
- Ledesma DT, and Scarpetta SG. 2018. The skull of the gerrhonotine lizard *Elgaria panamintina* (Squamata: Anguidae). *Plos ONE* **13**(6):e0199584.
- Lee, M. S. Y. 1998. Convergent evolution and character correlation in burrowing reptiles: towards a resolution of squamate phylogeny. Biol. J. Linnean Soc. **65**:369–453.
- Li, J-L. 1985. A new lizard from the Late Jurassic of Subei, Gansu. Vertebrata PalAsiatica 23:13-18.
- Linnaeus, C. 1758. Systema naturae per regna tria naturae, secundum classes, ordines, genera,species, cum characteribus, differentiis, synonymis, locis. 10th Ed. Laurentii Salvii, Holmiae.
- Longrich NR, Bhullar BAS, and Gauthier JA. 2012. Mass extinction of lizards and snakes at the Cretaceous–Paleogene boundary. *Proceedings of the National Academy of Sciences*, **109**(52): 21396-21401.
- Longrich, NR, Vinther, J, Pyron, RA, Pisani D, and Gauthier, JA. 2015. Biogeography of worm lizards (Amphisbaenia) driven by end-Cretaceous mass extinction. *Proceedings of the Royal Society B: Biological Sciences* **282**(1806):20143034.
- Macrì, S, Savriama, Y, Khan, I, and Di-Poï, N. 2019. Comparative analysis of squamate brains unveils multi-level variation in cerebellar architecture associated with locomotor specialization. *Nature Communications* **10**(1):1-16.
- Mahler DL, and Kearney M. 2006. The palatal dentition in squamate reptiles: morphology, development, attachment, and replacement. *Fieldiana Zoology* **108**:1-61.
- Maidment SCR, and Muxworthy A. 2019. A chronostratigraphic framework for the Upper Jurassic Morrison Formation, western USA. *Journal of Sedimentary Research* **89**(10):1017-1038.
- Maisano JA, Bell CJ, Gauthier JA, Rowe T. 2002. The osteoderms and palpebral in *Lanthanotus borneensis* (Squamata: Anguimorpha). *Journal of Herpetology* **36**: 678– 682.
- Matsumoto R, and Evans SE. 2017. The palatal dentition of tetrapods and its functional significance. *Journal of Anatomy* **230**(1):47-65.

- McDowell, S. B., Jr, and C. M. Bogert. 1954. The systematic position of *Lanthanotus borneensis*, and the affinities of the anguinomorphan lizards. Bulletin of the American Museum of Natural History 105:1–142.
- Mertens, R., 1937. Reptilien und Amphibien aus dem südlichen Inner-Afrika. Abhandlungen der Senckenbergischen Naturforschenden Gesellschaft (Frankfurt) 435:1–23.
- Müller J, Hipsley CA, Head JJ, Kardjilov N, Hilger A, Wuttke M, and Reisz RR. 2011. Eocene lizard from Germany reveals amphisbaenian origins. *Nature* **473**(7347):364-367.
- Nance HA. 2007. Cranial osteology of the African gerrhosaurid *Angolosaurus skoogi* (Squamata; Gerrhosauridae). *African Journal of Herpetology* **56**(1):39-75.
- Nash DF, and Tanner WW. 1970. A comparative study of the head and thoracic osteology and myology of the skinks *Eumeces gilberti* Van Denburgh and *Eumeces skiltonianus* (Baird and Girard). *Brigham Young University Science Bulletin, Biological Series* **12**(2):1-32.
- Norell MA, Gao K-Q, and Conrad J. 2008. A new platynotan lizard (Diapsida: Squamata) from the Late Cretaceous Gobi Desert (Ömnögov), Mongolia. *American Museum Novitates* **3605**:1-22.
- Nydam RL, and Cifelli RL. 2002. Lizards from the Lower Cretaceous (Aptian–Albian) Antlers and Cloverly Formations. *Journal of Vertebrate Paleontology* **22**(2):286-298.
- Nydam, R. L., and B. M Fitzpatrick, 2009. The occurrence of *Contogenys*-like lizards in the Late Cretaceous and early Tertiary of the Western Interior of the U.S.A.
- Oppel, M. 1811. Die Ordnungen, Familien und Gattungen der Reptilien als Prodrom einer Naturgeschichte derselben. J. Lindauer, Munich.
- Palci, A, Hutchinson, MN, Caldwell, MW, and Lee, MS. 2017. The morphology of the inner ear of squamate reptiles and its bearing on the origin of snakes. *Royal Society Open Science* **4**(8):170685.
- Paluh DJ, and Bauer AM. 2017. Comparative skull anatomy of terrestrial and crevice-dwelling *Trachylepis* skinks (Squamata: Scincidae) with a survey of resources in scincid cranial osteology. *PLoS ONE* **12**(9):e0184414.
- Petermann H, and Gauthier JA. 2020. Skeletochronology Reconciles Differences in Growth Strategies and Longevity in the Common Chuckwalla (*Sauromalus ater*) with

Implications for Squamate Life-History Studies. *Ichthyology and Herpetology* **108**(1):72-82.

- Presch W. 1980. Evolutionary history of the South American mitomicroteiid lizards (Teiidae: Gymnophthalminae). *Copeia* 1:36-56.
- Prothero DR, and Estes R. 1980. Late Jurassic lizards from Como Bluff, Wyoming and their palaeobiogeographic significance. *Nature* **286**(5772):484-486.
- Pyron RA, Burbrink FT, and Wiens JJ. 2013. A phylogeny and revised classification of Squamata, including 4161 species of lizards and snakes. *BMC Evolutionary Biology* **13**(1):1-54.
- Reeder TW, Townsend TM, Mulcahy DG, Noonan BP, Wood Jr PL, Sites Jr JW, and Wiens JJ. 2015. Integrated analyses resolve conflicts over squamate reptile phylogeny and reveal unexpected placements for fossil taxa. *PloS ONE* **10**(3):e0118199.
- Reynoso V-H, and Callison G. 2000. A new scincomorph lizard from the Early Cretaceous of Puebla, México. *Zoological Journal of the Linnean Society* **130**(2):183-212.
- Reynoso VH. 1998. *Huehuecuetzpalli mixtecus* gen. et sp. nov: a basal squamate (Reptilia) from the Early Cretaceous of Tepexi de Rodríguez, Central México. *Proceedings of the Royal Society B:Biological Sciences* **353**(1367):477-500.
- Richter A. 1994. Lacertilia aus der unteren kreide von Uña und Galve (Spanien) und Anoual (Marokko). *Berl. Geowissen. Abh. Reihe. B. Palaeobiol.* **14**:1–147.
- Rieppel O, and Grande L. 2007. The anatomy of the fossil varanid lizard *Saniwa ensidens* Leidy, 1870, based on a newly discovered complete skeleton. *Journal of Paleontology* **81**(4):643-665.
- Rieppel O, Conrad JL, and Maisano JA. 2007. New morphological data for *Eosaniwa koehni* Haubold, 1977 and a revised phylogenetic analysis. *Journal of Paleontology* **81**(4):760-769.
- Rieppel O, Gauthier JA, and Maisano J. 2008. Comparative morphology of the dermal palate in squamate reptiles, with comments on phylogenetic implications. *Zoological Journal of the Linnean Society* **152**(1):131-152.
- Romer, A. S. 1956. Osteology of the reptiles. University of Chicago Press, Chicago,

- Rowe, T. and J. Gauthier, 1992. Ancestry, paleontology, and the definition of the name Mammalia. Systematic Biology, 41(3): 372-378.
- Savage JM. 1963. Studies on the lizard family Xantusiidae IV. The genera. *Contributions in Science* 71: 1– 38.
- Schatzinger RA. 1980. New species of *Palaeoxantusia* (Reptilia: Sauria) from the Uintan (Eocene) of San Diego Co., California. *Journal of Paleontology* 54:46CM71.
- Schwenk K. 1993. The evolution of chemoreception in squamate reptiles: a phylogenetic approach. *Brain, Behavior, and Evolution* **41**:124–137.
- Schwenk K. 1995. Of tongues and noses: chemoreception in lizards and snakes. *Trends in Ecology and Evolution* **10**:7–12.
- Seiffert, J. 1973. Upper Jurassic lizards from central Portugal. Memores Servicos Geologicos de Portugal (Nova Serie) 22:1–85.
- Shaw, G., and R. P. Nodder. 1812. The Naturalist's Miscellany. Printed for Nodder & Co., London.
- Simões TR, Caldwell MW, Nydam RL, and Jiménez-Huidobro P. 2016. Osteology, phylogeny, and functional morphology of two Jurassic lizard species and the early evolution of scansoriality in geckoes. *Zoological Journal of the Linnean Society* **180**(1):216-241.
- Simões TR, Caldwell MW, Tałanda M, Bernardi M, Palci A, Vernygora O, Bernardini F, Mancini L, and Nydam RL. 2018. The origin of squamates revealed by a Middle Triassic lizard from the Italian Alps. *Nature* **557**(7707):706-709.
- Smith KT, and Habersetzer J. 2021. The anatomy, phylogenetic relationships, and autecology of the carnivorous lizard "*Saniwa*" *feisti* Stritzke, 1983 from the Eocene of Messel, Germany. *Comptes Rendus Palevol* **20**(23): 441-506.
- Stanley, E. L. 2013. Systematics and morphological diversification of the Cordylidae (Squamata). Dissertation, Richard Gilder Graduate School, American Museum of Natural History.
- Suarez, C.A., Frederickson, J., Cifelli, R.L., Pittman, J.G., Nydam, R.L., Hunt-Foster, R.K. and Morgan, K. 2021. A new vertebrate fauna from the Lower Cretaceous Holly Creek Formation of the Trinity Group, southwest Arkansas, USA. *PeerJ* **9**:p.e12242.

- Tałanda, M. 2016. Cretaceous roots of the amphisbaenian lizards. *Zoologica Scripta* **45**(1), 1-8.
- Tałanda, M. 2018. An exceptionally preserved Jurassic skink suggests lizard diversification preceded fragmentation of Pangaea. Paleontology 61(5):659-677.
- Tanner LH, Galli KK, and Lucas SG. 2014. Pedogenic and lacustrine features of the Brushy Basin Member of the Upper Jurassic Morrison Formation in western Colorado: reassessing the paleoclimatic interpretations. *Volumina Jurassica* **12**(2):115-130.
- Thorn KM, Hutchinson MN, Lee MSY, Brown NJ, Camens AB, and Worthy TH. 2021. A new species of *Proegernia* from the Namba Formation in South Australia and the early evolution and environment of Australian egerniine skinks. *Royal Society Open Science* **8**(2):201686.
- Triviño, LN, Albino, AM, Dozo, MT, and Williams, JD. 2018. First natural endocranial cast of a fossil snake (Cretaceous of Patagonia, Argentina). *The Anatomical Record* **301**(1):9-20.
- Trujillo KC, Foster JR, Hunt-Foster RK, and Chamberlain KR. 2014. AU/Pb age for the Mygatt-Moore Quarry, Upper Jurassic Morrison Formation, Mesa County, Colorado. *Volumina Jurassica* **12**(2):107-114.
- Turner CE, and Peterson F. 2004. Reconstruction of the Upper Jurassic Morrison Formation extinct ecosystem—a synthesis. *Sedimentary Geology* **167**(3-4):309-355.
- Uetz, P., P. Freed, R. Aguilar, and J. Hošek (eds.) 2021. The Reptile Database, http://www.reptile-database.org, accessed 2021.
- Vicario, S., A. Caccone, and J. Gauthier, 2003. Xantusiid "Night" lizards: a puzzling phylogenetic problem revisited using Likelihood-based Bayesian methods on mtDNA sequences. Journal of Molecular Phylogenetics and Evolution. 26: 243-261.
- Vidal, N., and S. B. Hedges. 2005. The phylogeny of squamate reptiles (lizards, snakes, and amphisbaenians) inferred from nine nuclear protein-coding genes. C. R. Biologies 328: 1000–1008.
- Villa A, Daza JD, Bauer AM, and Delfino M. 2018. Comparative cranial osteology of European gekkotans (Reptilia, Squamata). *Zoological Journal of the Linnean Society*, **184**(3):857-895.
- Wiegmann, A. F. A. 1828. Beyträge zur Amphibienkunde. Isis von Oken 21(4):364-383

- Yi, H, and Norell, MA. 2015. The burrowing origin of modern snakes. *Science Advances* **1**(10):e1500743.
- Zheng Y, and Wiens JJ. 2016. Combining phylogenomic and supermatrix approaches, and a time-calibrated phylogeny for squamate reptiles (lizards and snakes) based on 52 genes and 4162 species. *Molecular Phylogenetics and Evolution* **94**:537-547.

Supplementary Figure 12.
