# Supplementary material for: Evolutionary origins of the prolonged extant squamate radiation
Source: Nat Commun. 2022 Nov 29;13:7087. doi: 10.1038/s41467-022-34217-5 (PMC9708687; doi:10.1038/s41467-022-34217-5)
Supplement: Supplementary file 2 — Description of Additional Supplementary Files [file 41467_2022_34217_MOESM2_ESM.pdf]

Description of Additional Supplementary Files

File Name: Supplementary Data 1
Description: Supplementary †*Eoscincus ornatus* Segmentation Data.

File Name: Supplementary Data 2
Description: Supplementary †*Microteras borealis* Segmentation Data.

File Name: Supplementary Data 3
Description: Supplementary Parsimony Analysis Input and Output.

File Name: Supplementary Data 4
Description: Supplementary Bayesian Analysis Input and Output.

File Name: Supplementary Data 5
Description: Supplementary Bayesian Analysis With Molecular Constraints Input and Output.

File Name: Supplementary Data 6
Description: Supplementary Figures in High Definition.
