# Supplementary figures and images for: Evolutionary origins of the prolonged extant squamate radiation
Source: Nat Commun. 2022 Nov 29;13:7087. doi: 10.1038/s41467-022-34217-5 (PMC9708687; doi:10.1038/s41467-022-34217-5)

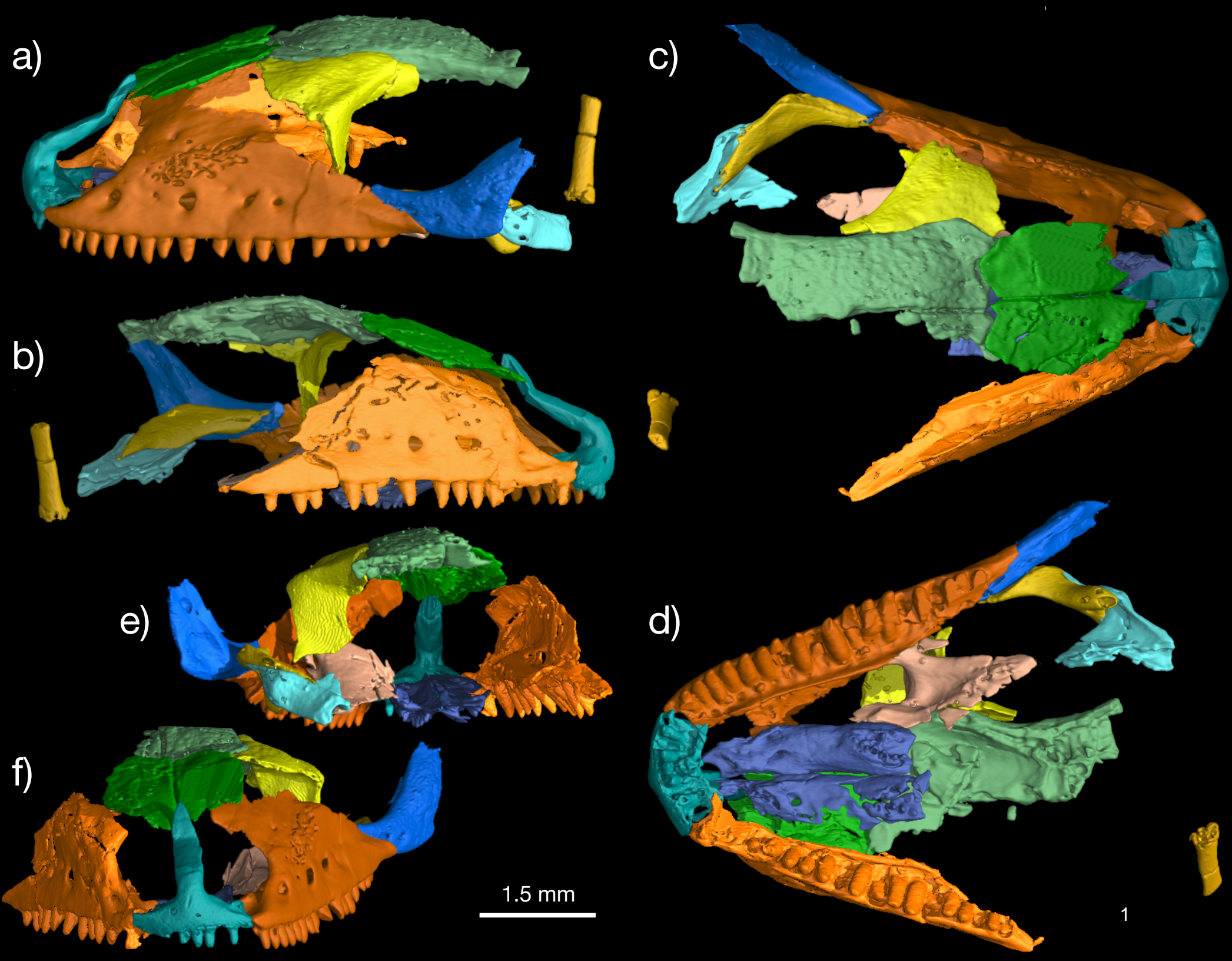

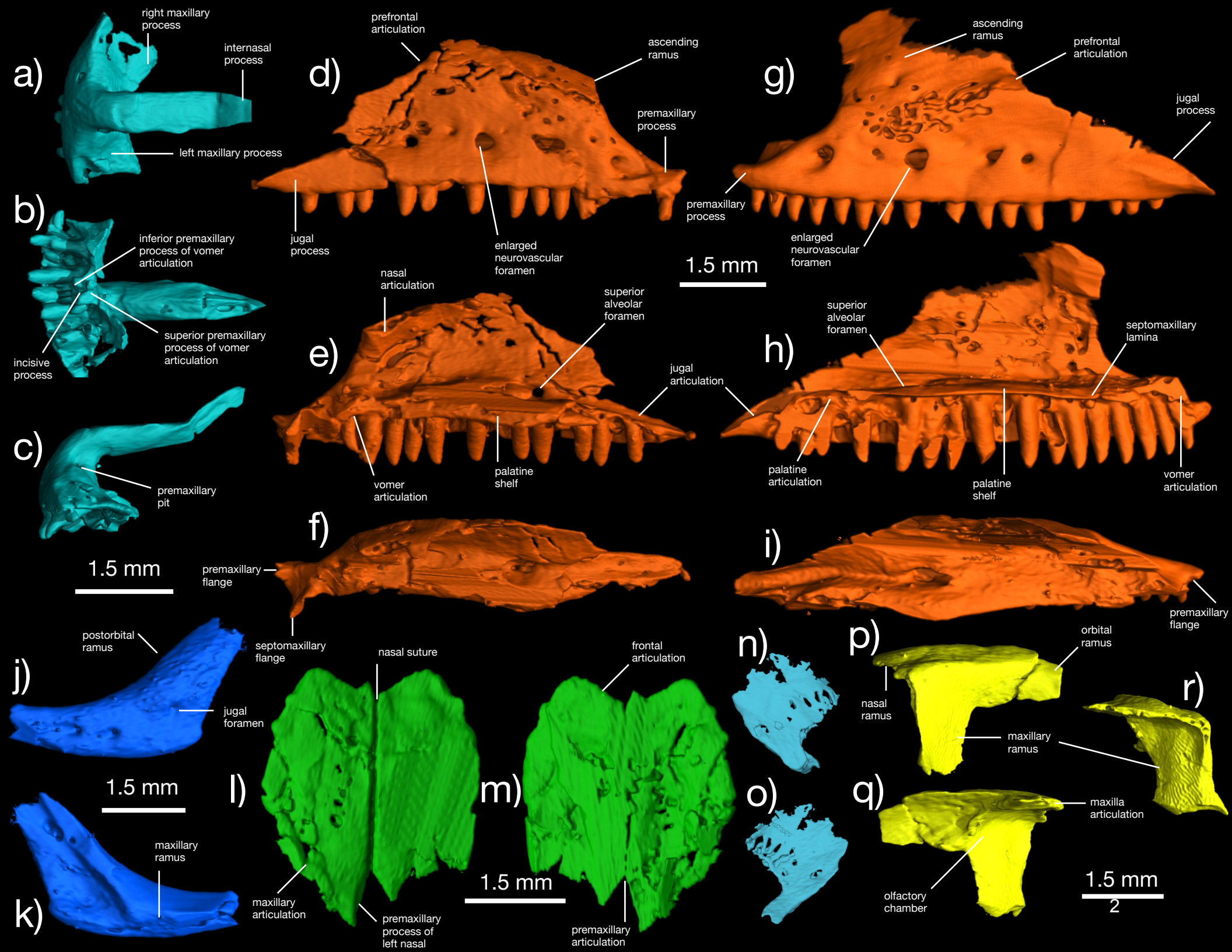

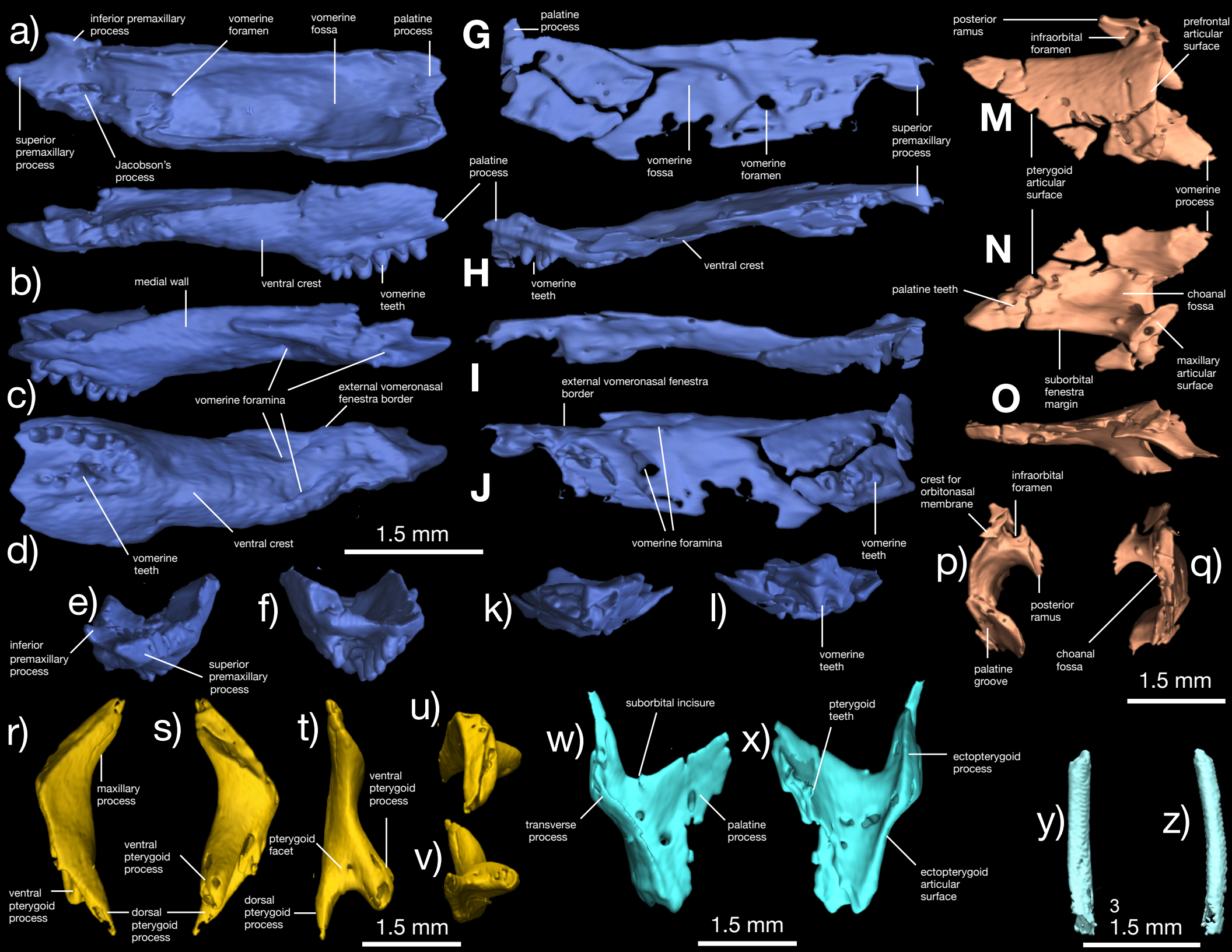

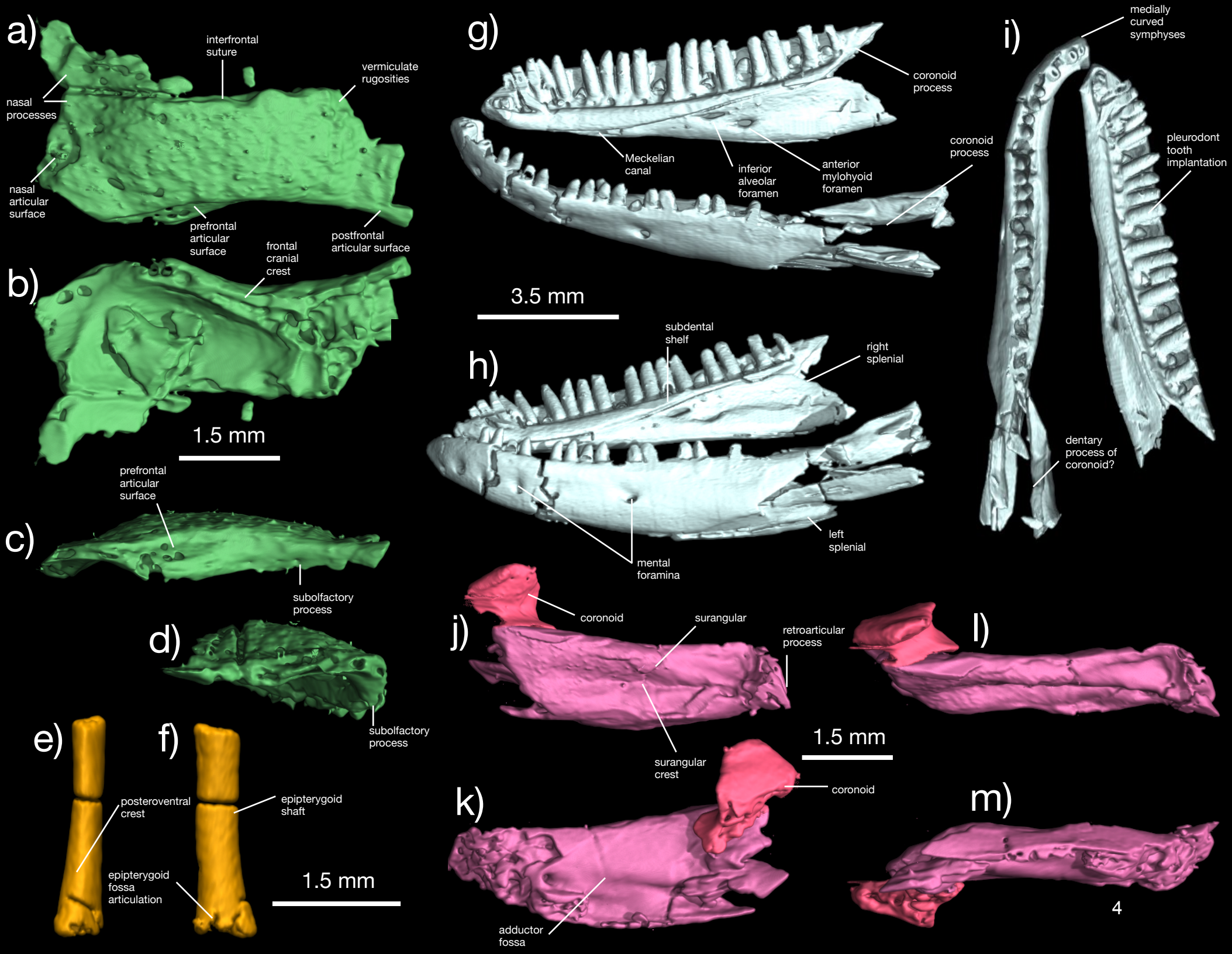

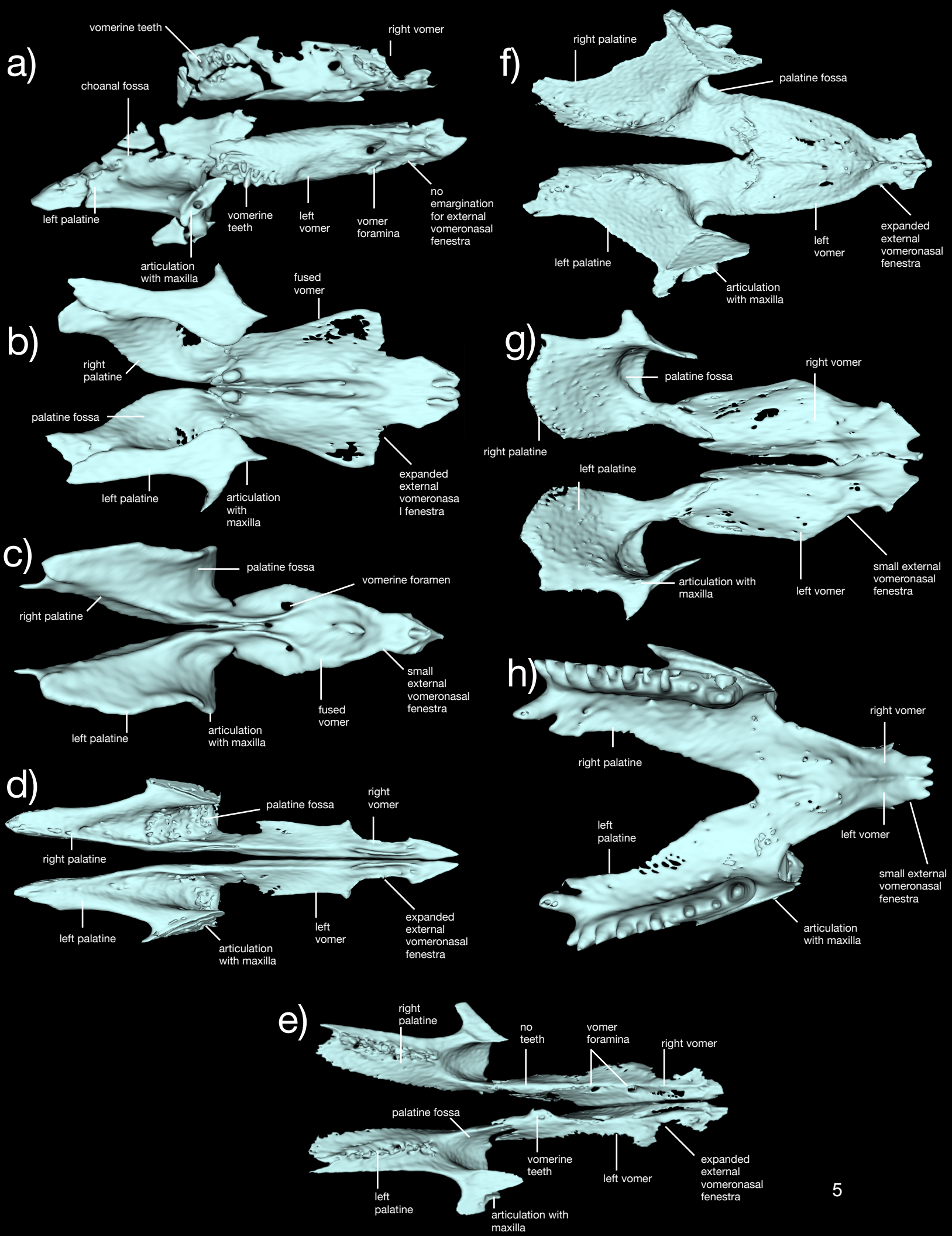

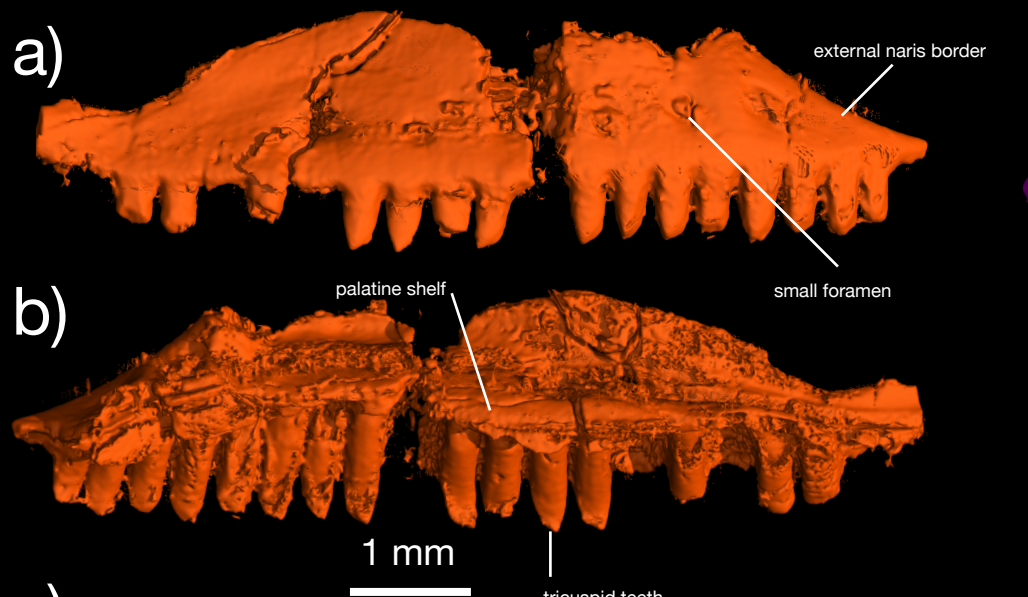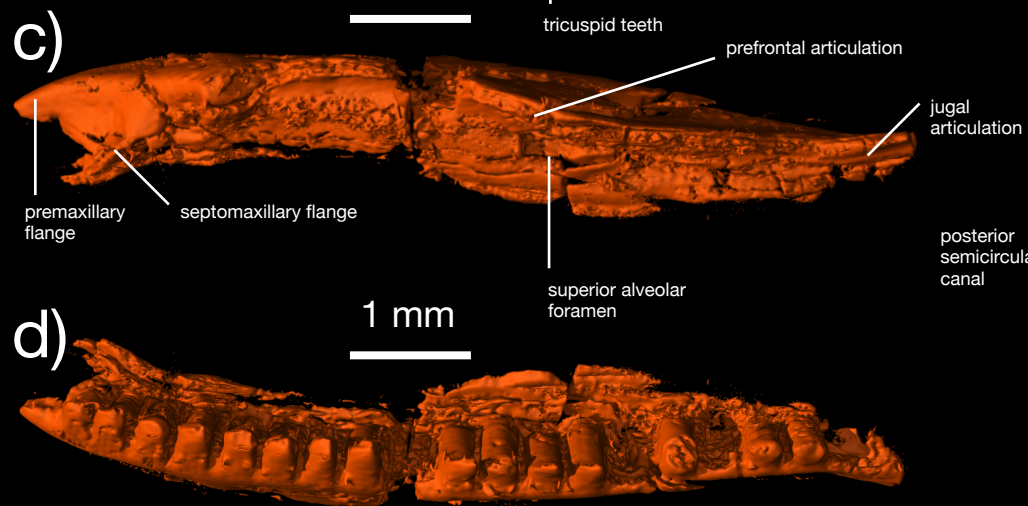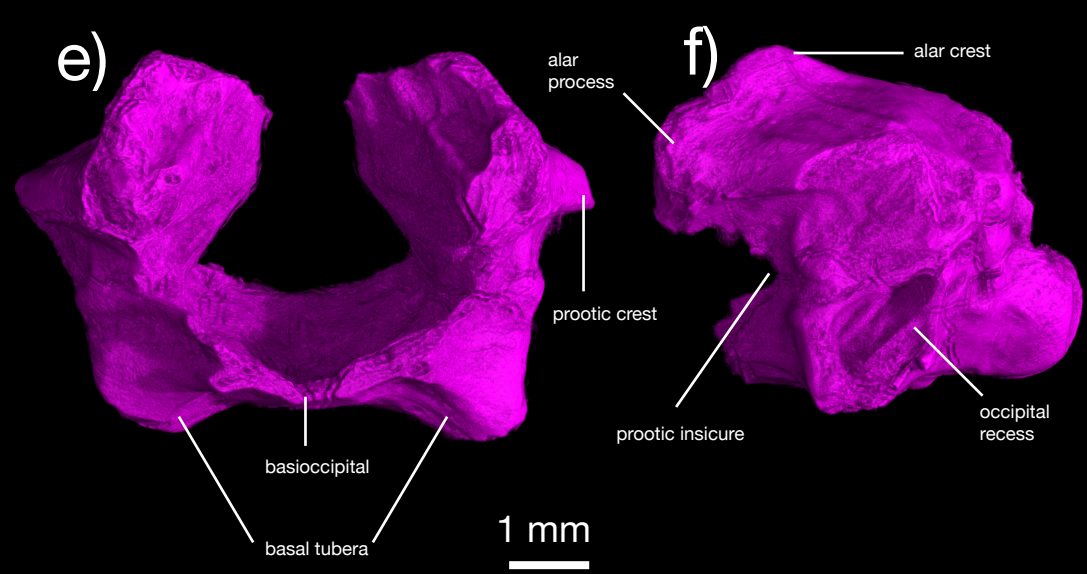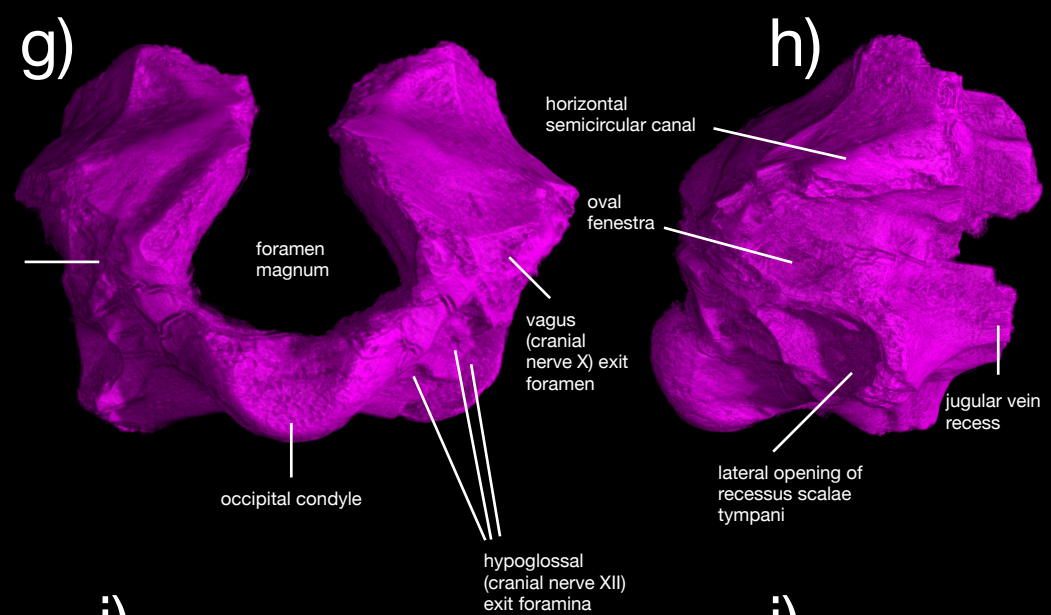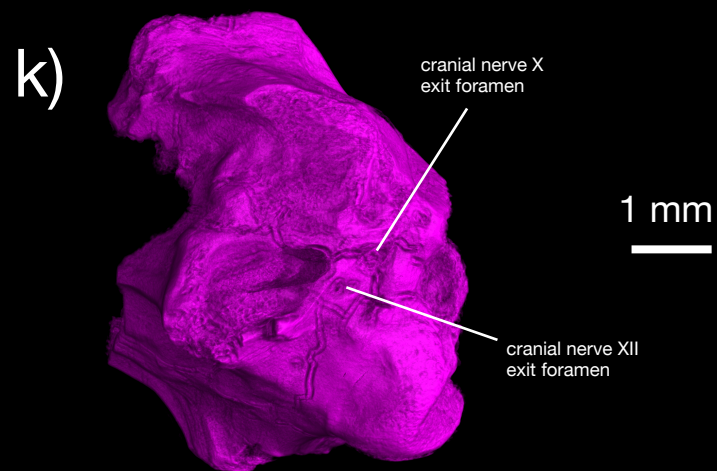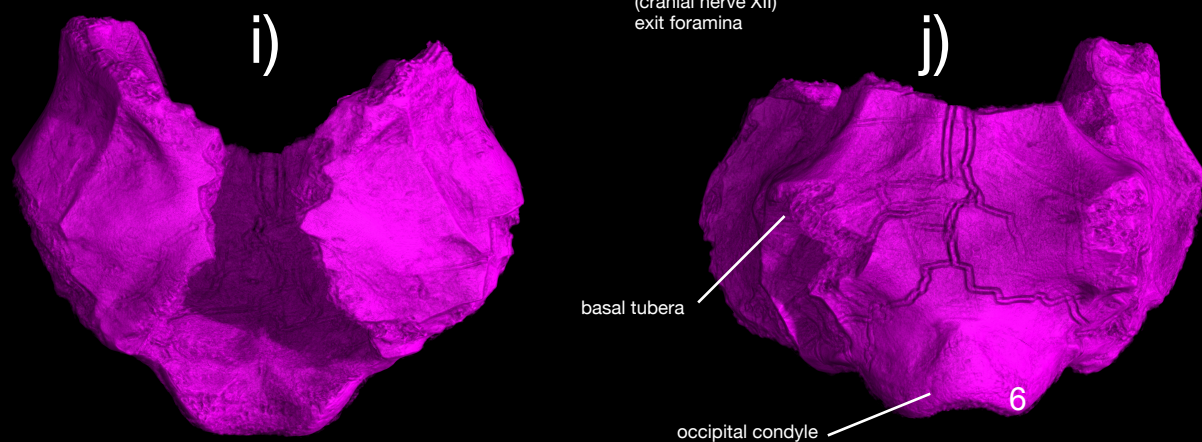

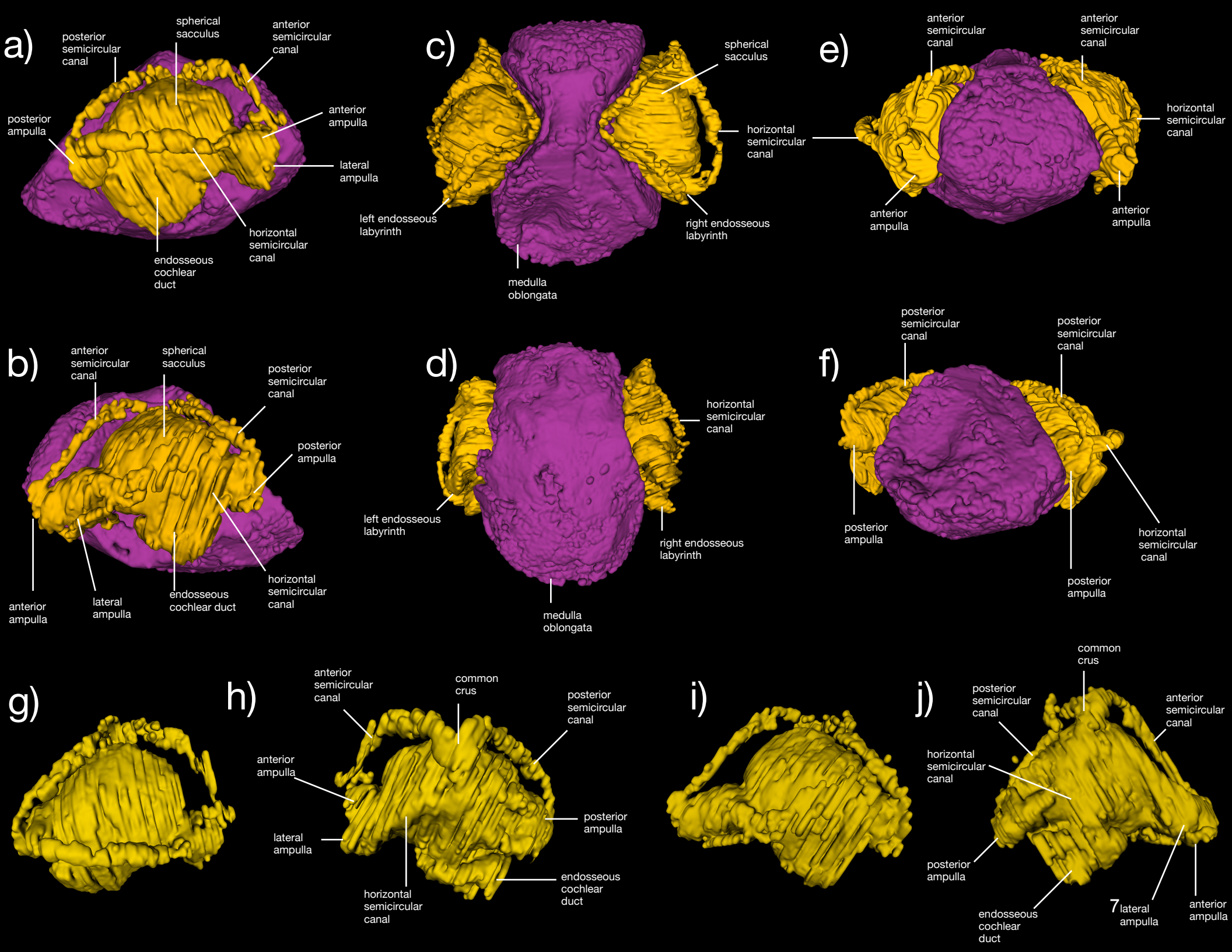

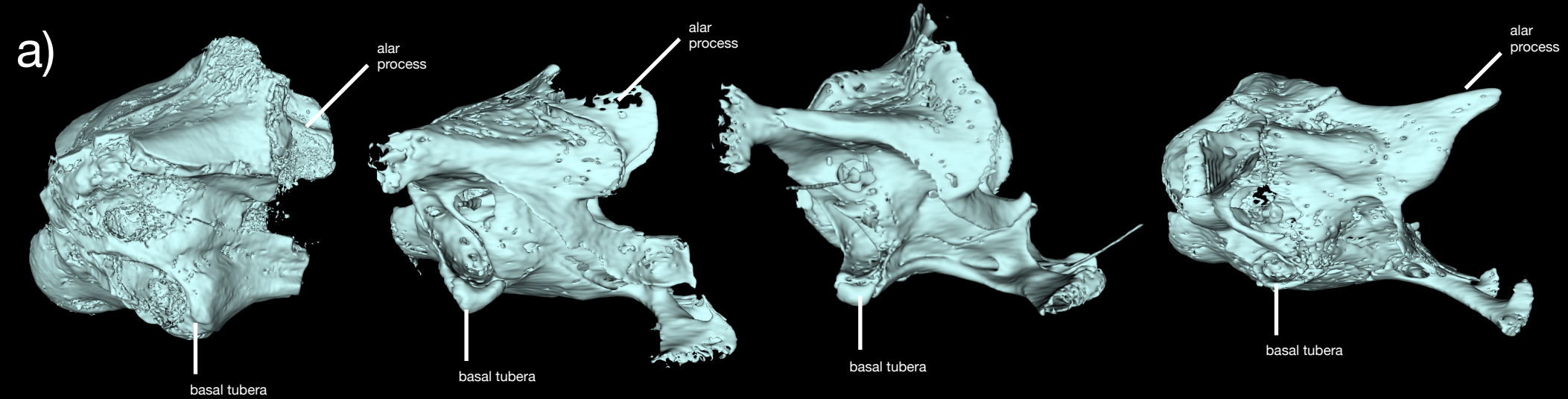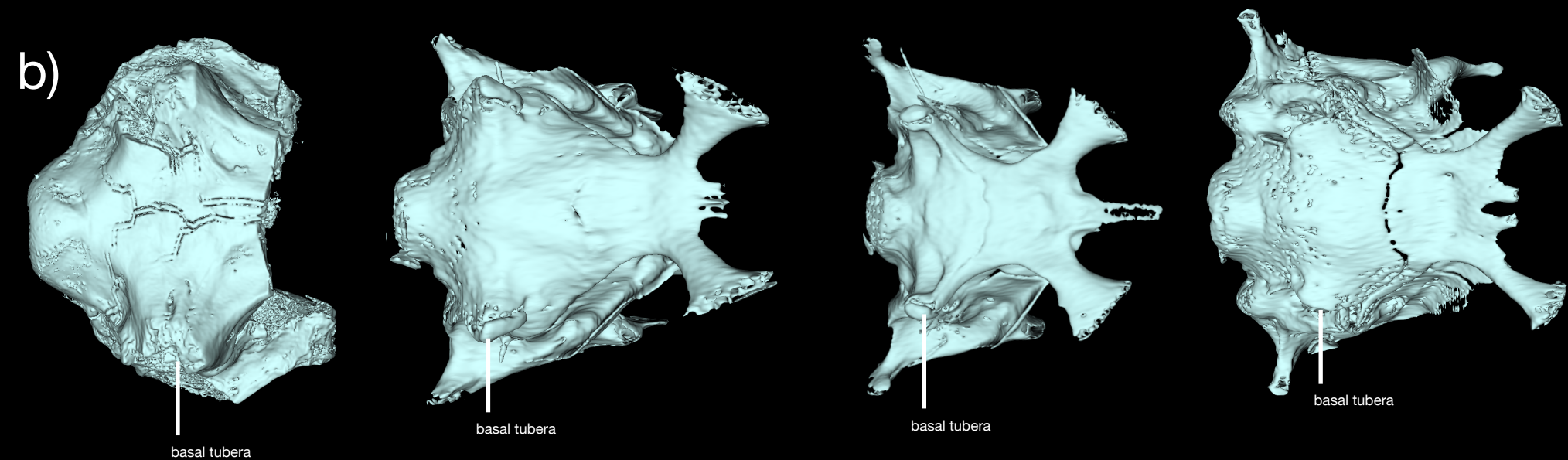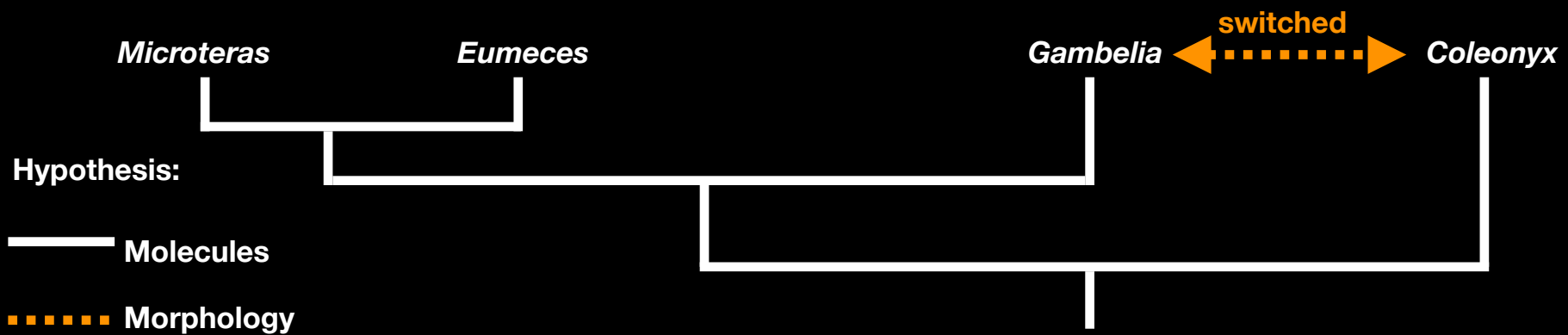

a)

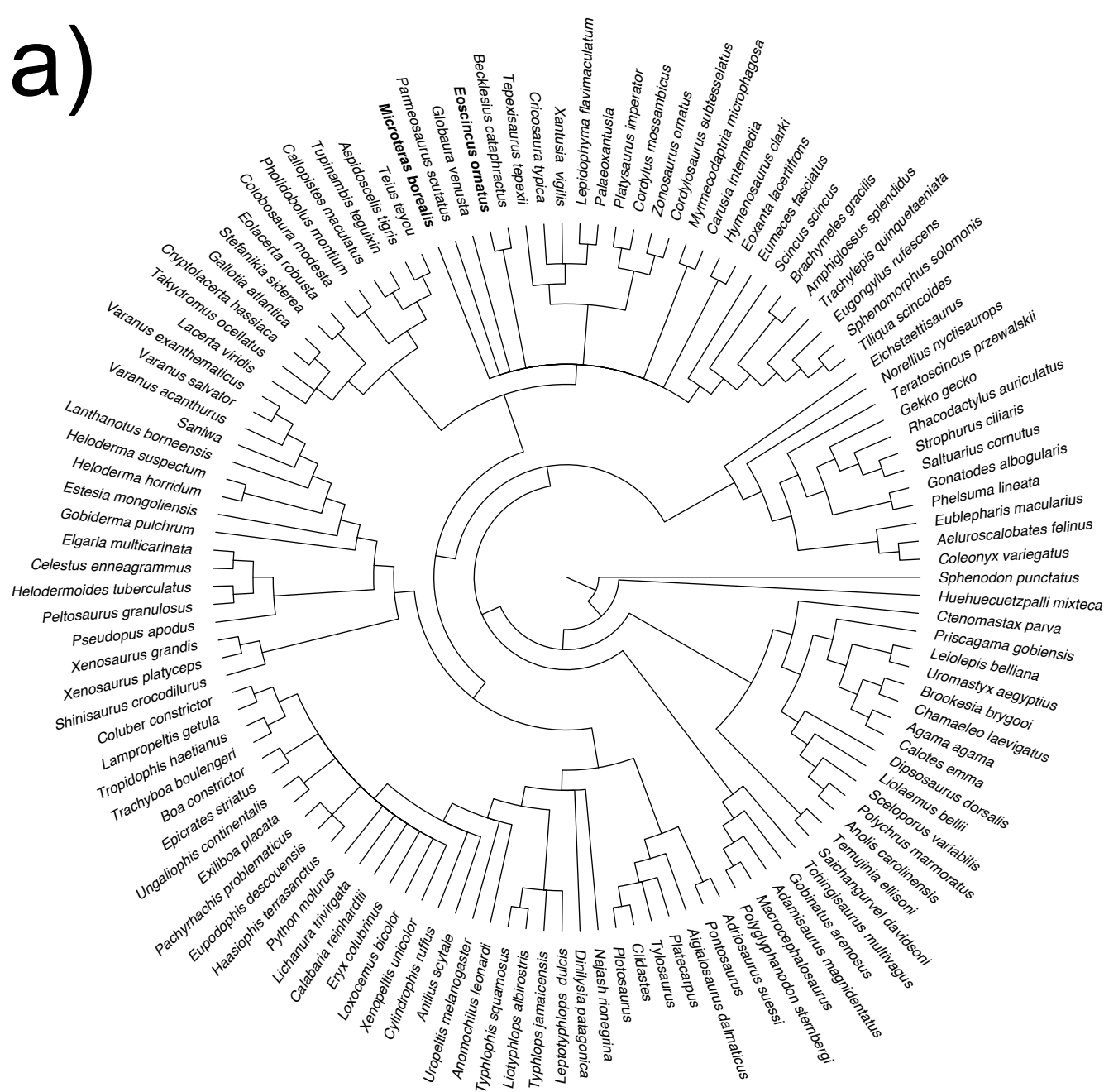

b)

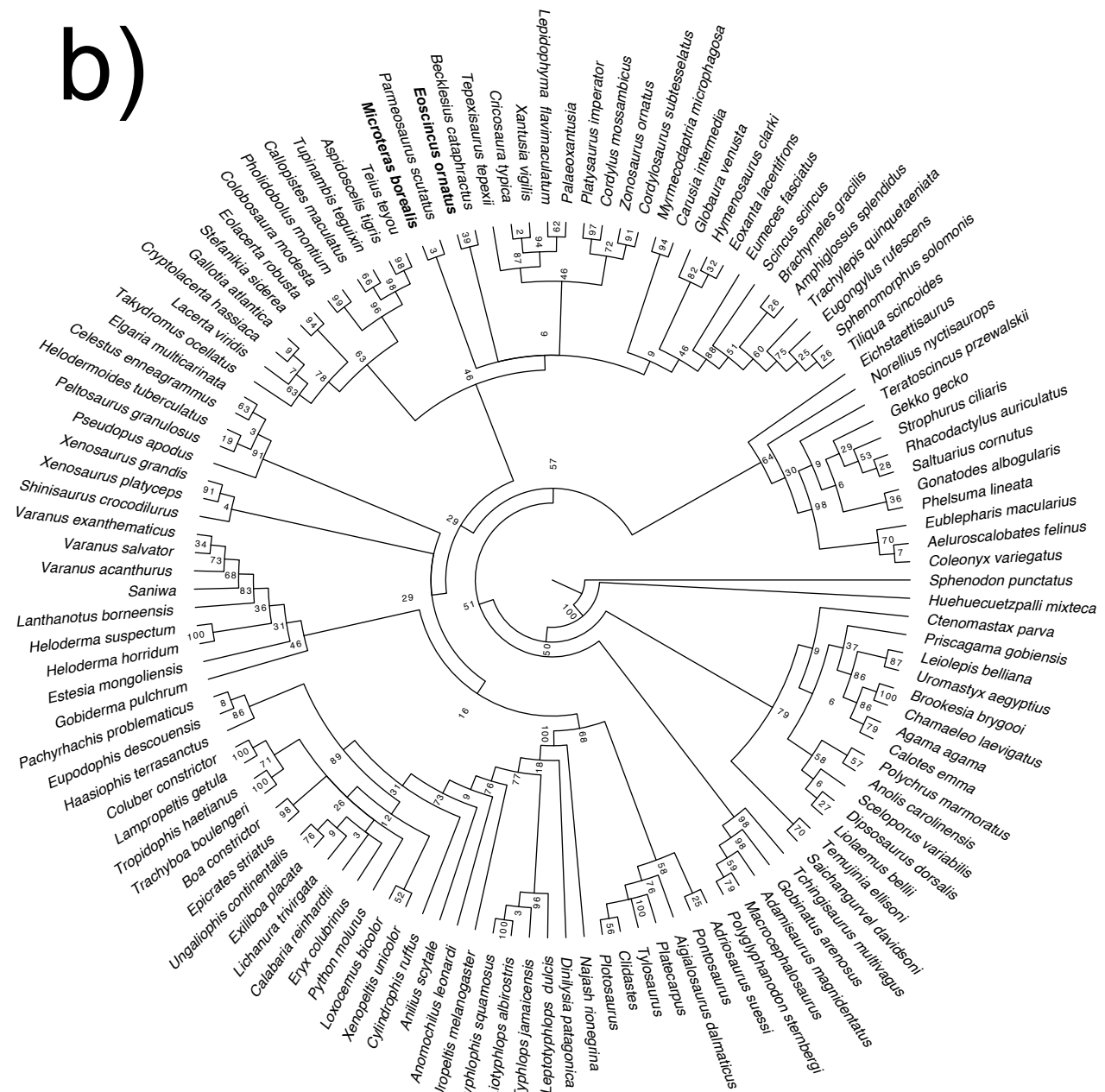

**b)**

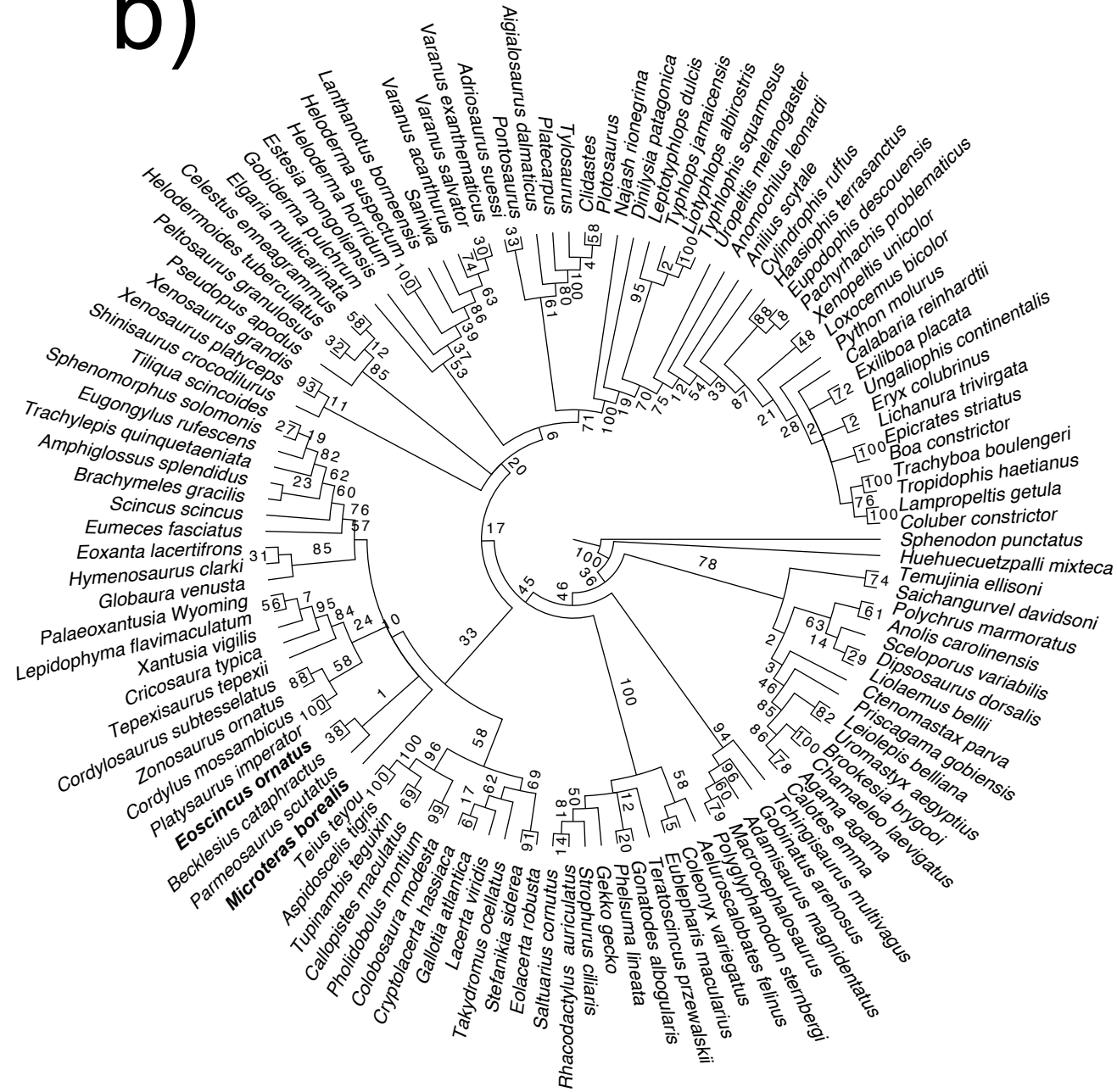

**b)**

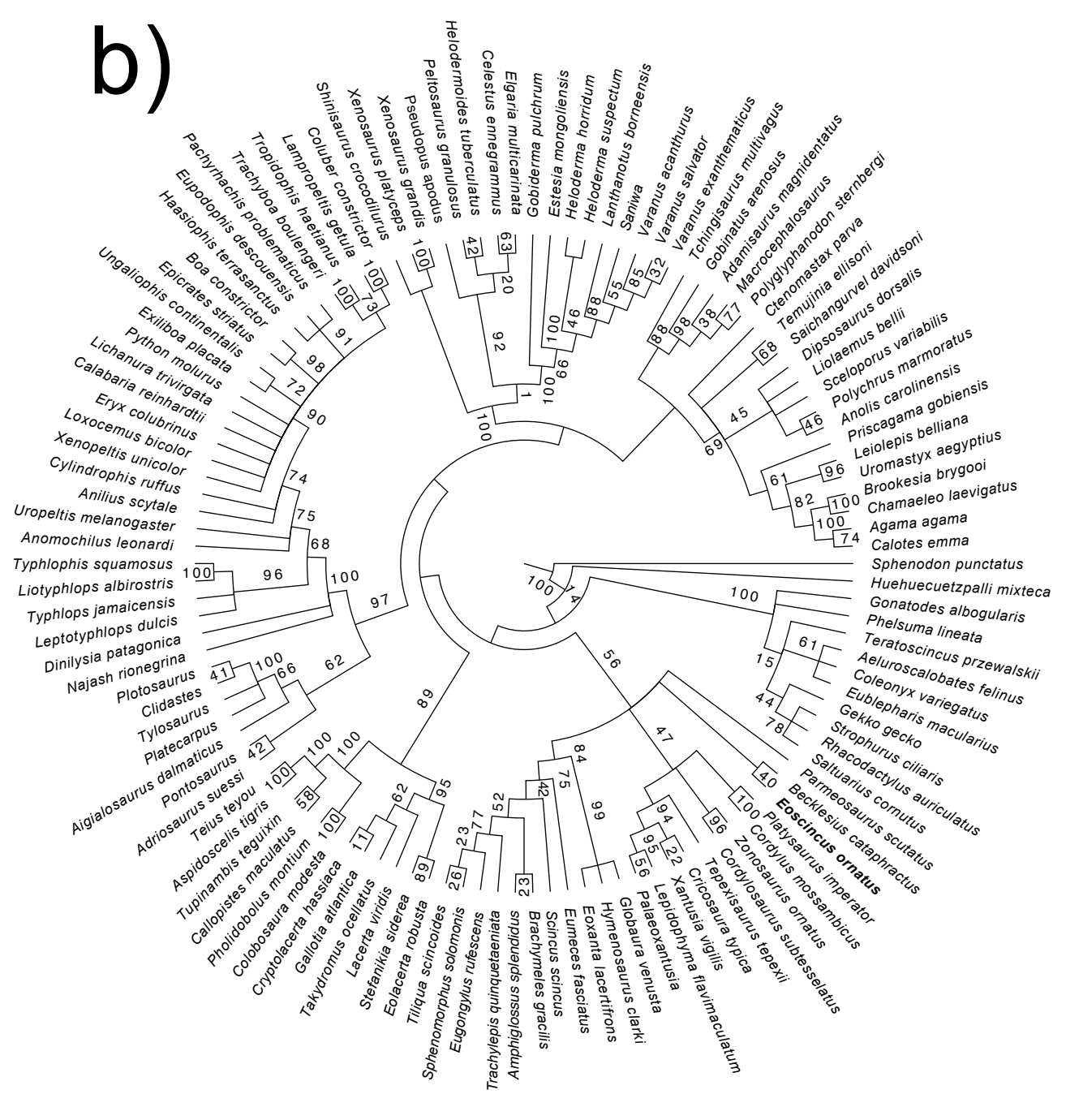

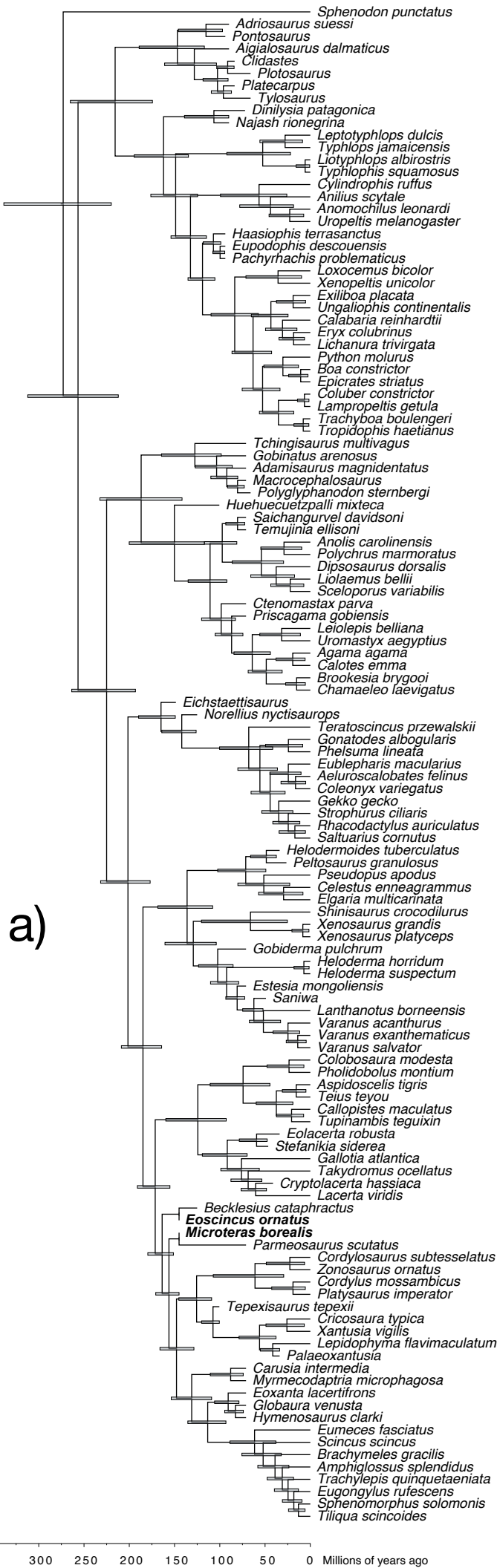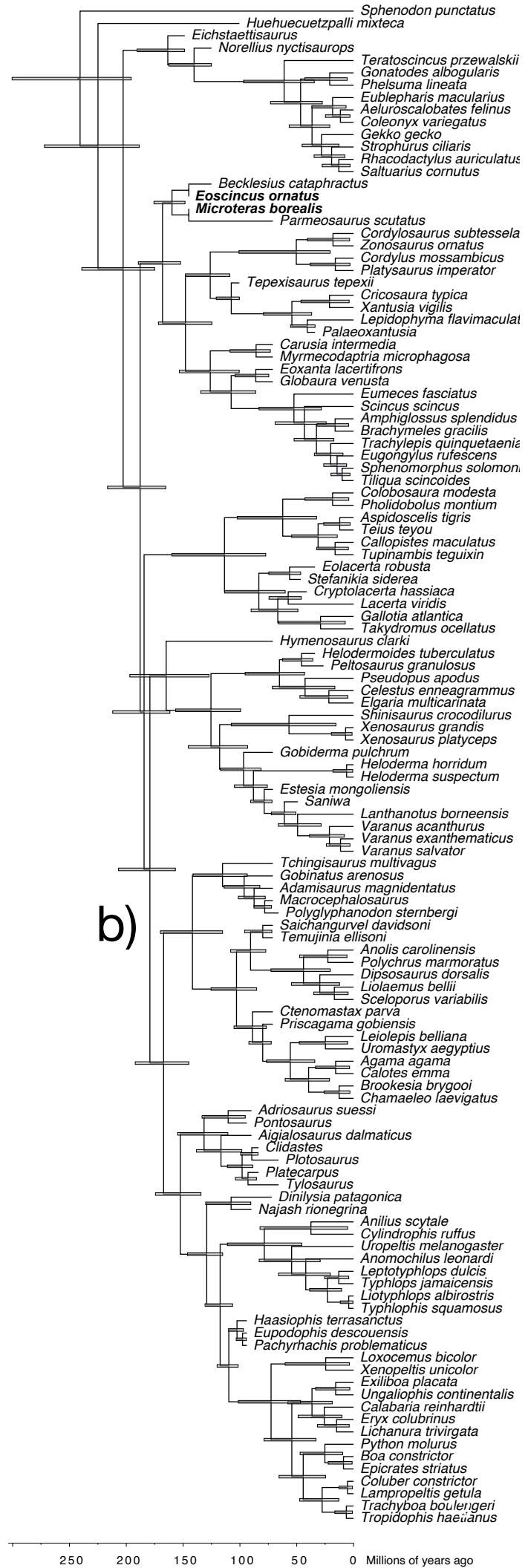

Supplement: Supplementary file 8 — Supplementary Data 6 [file 41467_2022_34217_MOESM8_ESM.pdf]
